# Phage Display Driven Identification and Computational Mapping of Macrocyclic Peptides Targeting RhoA G17V

**DOI:** 10.1021/acs.biochem.6c00058

**Published:** 2026-04-15

**Authors:** Sebin Abraham, Chaoyang Zhu, Lai Hoang Son Le, Yugendar R. Alugubelli, Tatsuki Nonomura, Yun Huang, J. Trae Hampton, Yubin Zhou, Wenshe Ray Liu

**Affiliations:** † Texas A&M Drug Discovery Center and Department of Chemistry, 14736 Texas A&M University, College Station, Texas 77843, United States; ‡ Institute of Biosciences and Technology and Department of Translational Medical Sciences, College of Medicine, Texas A&M University, Houston, Texas 77030, United States; § Department of Biochemistry and Biophysics, Texas A&M University, College Station, Texas 77843, United States; ∥ Department of Cell Biology and Genetics, College of Medicine, Texas A&M University, College Station, Texas 77843, United States; ⊥ Department of Pharmaceutical Sciences, Texas A&M University, College Station, Texas 77843, United States

## Abstract

Mutant RhoA G17V is a clinically significant yet historically undruggable oncogenic GTPase that drives angioimmunoblastic T-cell lymphoma through a neomorphic interaction with the guanine nucleotide exchange factor Vav1. Its rigid GTPase fold, absence of deep binding pockets, and transient protein-protein interfaces have hindered conventional small-molecule approaches, creating a critical need for alternative therapeutic modalities. Here, we report a systematic strategy to target RhoA G17V using macrocyclic peptides. Two complementary phage-displayed cyclic peptide libraries, an AcrK-mediated 10-mer cyclic library and a CAmCBT-cyclized 12-mer library, were subjected to high-stringency biopanning against recombinant RhoA G17V. While the 10-mer library yielded moderate-affinity binders, the 12-mer library enabled the discovery of Z1, a macrocyclic peptide with submicromolar affinity (K_D_ = 136 nM), representing the highest-affinity peptide reported for RhoA G17V to date. Computational docking combined with long-timescale molecular dynamics simulations revealed a stable peptide–protein interaction governed by cooperative hydrophobic and electrostatic interactions. Systematic alanine scanning mutagenesis experimentally validated the predicted binding determinants, confirming the key residues within the macrocycle. Collectively, this work establishes macrocyclic phage display as a powerful and generalizable platform for discovering high-affinity ligands against challenging mutant GTPases and lays a foundation for the development of precision peptide-based therapeutics.

## Graphical abstract

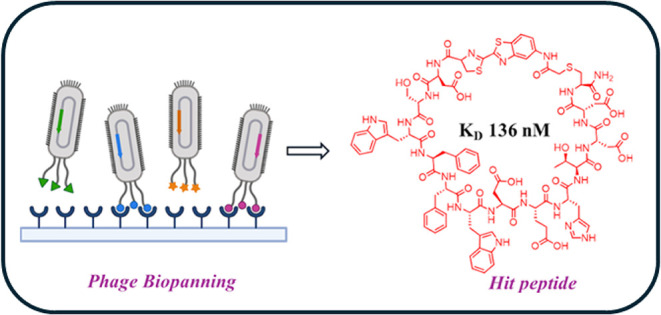

## Introduction

GTPases are a diverse superfamily of molecular switches that control key cellular processes, including signal transduction, intracellular trafficking, cell cycle regulation, cytoskeletal organization, and immune responses.^1–5^ These proteins cycle between an active GTP-bound and inactive GDP-bound state, enabling precise regulation in response to extracellular and intracellular cues. The human GTPase superfamily comprises several subfamilies, Ras, Rho, Rab, Arf, and Ran, each with specialized functions.^4^ The Ras family is a central regulator of cell proliferation and is frequently mutated in cancer,^6^ while Rho GTPases, such as RhoA, RAC1, and CDC42, orchestrate actin cytoskeleton remodeling, cell migration, polarity, and adhesion.^7–12^ Despite their biological importance, GTPases are considered challenging drug targets.^12–14^ Their high-affinity binding to GTP and GDP, coupled with a highly conserved nucleotide-binding pocket, limits opportunities for competitive inhibition.^15,16^ Moreover, their regulation and signaling involve transient, broad protein–protein interfaces, which are poorly suited for disruption by conventional small molecules.^17^ Antibodies, although potent binders, cannot readily cross cell membranes to access intracellular GTPases. These limitations have motivated the search for alternative therapeutic modalities.

Macrocyclic peptides have emerged as promising candidates for targeting GTPases. Their conformational rigidity enhances stability and resistance to proteolysis, improving pharmacokinetic properties.^18–20^ More importantly, their large and adaptable binding surfaces allow high-affinity, high-specificity recognition of flat protein interfaces, including those in GTPase–effector complexes. Cyclic peptides can be engineered to disrupt mutant-specific interactions or selectively modulate GTPase activity.^21,22^ Recent advances in phage and mRNA display technologies enable high-throughput screening of vast cyclic peptide libraries, accelerating the identification of potent binders.^23,24^ Notable successes include the discovery of cyclic peptides against KRAS with optimized affinity and membrane permeability,^25^ and the development of macrocycles that discriminate between nucleotide-bound states of Gαs.^26^

A clinically relevant target for such strategies is the RhoA G17V mutant, recurrently found in nearly 70% of cases of angioimmunoblastic T-cell lymphoma (AITL).^27^ This mutation is thought to abolish canonical nucleotide binding while conferring a neomorphic function via tight association with the guanine nucleotide exchange factor (GEF) VAV1, leading to its subsequent phosphorylation at Tyr174 by Src.^28^ The resulting hyperactivation of T-cell receptor (TCR) signaling drives AITL pathogenesis. Disrupting the G17V–VAV1 interaction is therefore a compelling therapeutic approach.

Here, we report the discovery of a potent cyclic peptide inhibitor of RhoA G17V. Using two complementary cyclic peptide phage libraries (Figure 1), we performed four rounds of alternating biopanning against recombinant RhoA G17V. Several high-affinity ligands were isolated from the selection campaign, among which peptide Z1 demonstrated the strongest interaction, exhibiting nanomolar affinity for the target. Computational docking and molecular dynamics simulations converged on a well-defined binding interface and predicted a set of key contact residues. Systematic alanine-scanning mutagenesis experimentally validated these computational predictions, confirming the critical determinants of Z1’s binding potency. These findings highlight the potential of cyclic peptides to selectively target pathogenic GTPase interactions, offering a new avenue for precision therapeutics in AITL.

**Figure 1:**
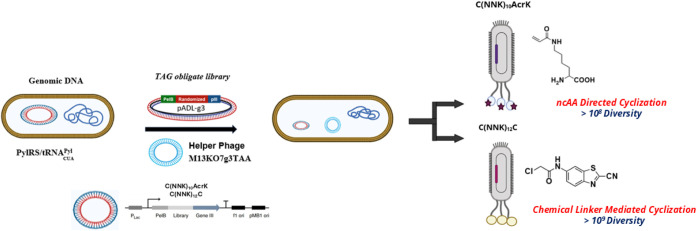
Macrocyclic phage libraries used for identifying peptide binders for RhoA G17V non-canonical amino acid (AcrK) mediated cyclization chemical linker mediated phage cyclization.

## Materials and Methods

Plasmid Construction: The DNA sequence encoding human RhoA (UniProt: P61586, residues 1–193) was amplified using primers GST-RhoA F (5′-GATCTGGTTCCGCGTGGATCCACCGCGAGCAACTCTCCG-3′) and GST-RhoA R (5′-GTCACGATGCGGCCGCTCGAGTTATTCGTGCCATTCGATTTTC-3′) with Phusion high-fidelity DNA polymerase. The PCR product was purified, digested with *Bam*HI and XhoI, and ligated into the corresponding sites of the pGEX expression vector, generating a construct encoding an N-terminal GST fusion, an HRV 3C protease cleavage site, the full-length RhoA coding sequence (with a G17V mutation), and a C-terminal Avi tag. Ligation mixtures were transformed into *Escherichia coli* BL21­(DE3) competent cells containing the BirA plasmid at 1 ng DNA per microliter, recovered in 2×YT medium at 37 °C, and plated on agar containing ampicillin and chloramphenicol. All resulting plasmids were isolated and verified by Sanger sequencing to confirm insert integrity and desired mutations.

The DNA sequence encoding the DH domain of human Vav1 (UniProt: P15498, residues 186–475) was amplified using primers VAV1-F (5′-GGAATTCCATATGGAGCCGGTTAGCATG-3′) and VAV1-R (5′-CCGCTCGAGGGTTTCGTTGTCACGTTT-3′) with Phusion high-fidelity DNA polymerase. The PCR product was purified, digested with NdeI and XhoI, and ligated into the corresponding sites of the pET-28a vector to generate a construct encoding the Vav1 DH domain fused to a C-terminal His6 tag. Ligation reactions were transformed into *Escherichia coli* BL21­(DE3) competent cells, recovered, and plated on kanamycin-containing agar. All resulting plasmids were isolated and verified by Sanger sequencing to confirm insert integrity, correct reading frame, and absence of unintended mutations.

### Protein Expression and Purification

Expression and Purification of Biotinylated GST-G17V (Avi-tagged): *E. coli* BL21­(DE3) cells were cotransformed with pGEX-G17V and pBirA (expressing BirA for in vivo biotinylation of the Avi-tagged G17V protein). A single colony was used to inoculate 10 mL of 2×YT medium supplemented with ampicillin (100 μg/mL) and chloramphenicol (34 μg/mL). The culture was grown overnight at 37 °C with shaking. The overnight culture was used to inoculate 1 L of 2×YT medium containing the same antibiotics and grown at 37 °C to an OD600 of 0.5. Protein expression and biotinylation were induced by the addition of 0.1 mM IPTG and 50 μM biotin, followed by incubation at 16 °C for 18 h. Cells were harvested by centrifugation (6000 rpm, 20 min, 4 °C), and cell pellets were resuspended in lysis buffer (DPBS, pH 7.5, 1 mM TCEP, 0.25 mM PMSF, 50 μM GDP, 5 mM MgCl_2_). Cells were lysed by sonication on ice. The lysate was clarified by centrifugation at 10,000g for 30 min at 4 °C. The clarified supernatant was applied to glutathione (GSH) resin and incubated for 1 h at 4 °C with gentle mixing. After extensive washing with lysis buffer (≥20 column volumes), the resin was treated with HRV 3C protease (0.5 mg/mL final concentration) for 3 h at 4 °C to cleave the N-terminal GST tag. Cleaved G17V protein was eluted from the resin using lysis buffer. Eluted protein fractions were concentrated and subjected to size-exclusion chromatography (SEC) using a Superdex 75 Increase 10/300 GL column pre-equilibrated with DPBS containing 1 mM TCEP. Peak fractions were pooled and analyzed by SDS-PAGE. Purified protein was aliquoted into PCR tubes (20 μL per aliquot), flash-frozen in liquid nitrogen, and stored at −80 °C until use.

### Expression and Purification of Vav1 DH Domain

Plasmid encoding the Vav1 DH domain was transformed into *E. coli* and expressed in 2×YT medium. A 10 mL overnight culture (100 μg/mL ampicillin) was inoculated into 1 L of 2×YT containing ampicillin and grown at 37 °C to OD600 ≈ 0.6. Protein expression was induced with 0.1 mM IPTG, and the culture was incubated at 18 °C for 18 h. Cells were harvested by centrifugation (6000 rpm, 20 min, 4 °C) and resuspended in 40 mL of lysis buffer (20 mM Tris–HCl, pH 7.5, 500 mM NaCl, 1 mM TCEP, 0.25 mM PMSF, 5 mM MgCl_2_). Cells were lysed by sonication on ice, and the lysate was clarified by centrifugation (10,000g, 30 min, 4 °C). The clarified lysate was incubated with Ni-NTA resin for 1 h at 4 °C. The resin was sequentially washed with lysis buffer containing 20 mM, 40 mM, and 80 mM imidazole. Bound protein was eluted using lysis buffer supplemented with 300 mM imidazole. Eluted fractions were concentrated and applied to a Superdex 200 Increase column equilibrated with 20 mM Tris-HCl, pH 7.5, 150 mM NaCl, 1 mM TCEP. Fractions corresponding to monomeric Vav1 DH were pooled and analyzed by SDS-PAGE. Purified protein was aliquoted into 20 μL portions, flash-frozen in liquid nitrogen, and stored at −80 °C until further use.

### Phage Library Expression (Acrk Mediated) and Cyclization

The C-X10-TAG library was transformed into electrocompetent TOP10 *E. coli* cells containing pEVOLCloDF-AcrKS (pEVOL-PylT-BuKRS with a CloDF origin of replication) and M13KO7­(pIII-) (M13KO7 helper phage with a nonsense TAA mutation in gIII). Transformed cells (6.2 × 10^9^ transformants) were added to 1.1 L of fresh 2×YT with ampicillin (100 μg/mL), chloramphenicol (34 μg/mL), and kanamycin (25 μg/mL) and incubated at 37 °C until OD600 = 0.5–0.8, at which point 1 mM IPTG, 5 mM nicotinamide, and 0.2% arabinose were added to induce phage expression. 100 mL of cells were added to a sterile flask to express negative control, while 5 mM of AcrK was added to the remaining 1 L of cells. Phages were expressed at 30 °C for 16 h. Sixteen hours postinduction, the culture was transferred to 50 mL tubes, cells were pelleted (3,750 g, 20 min), and the supernatant was decanted into new tubes. Phages were precipitated by the addition of appropriate amounts of 5× precipitation buffer (20% polyethylene glycol 8000 and 2.5 M NaCl) to afford a 1× solution and incubated at 4 °C for 1.5 h. The solution was centrifuged at 10,000 g for 30 min, the supernatant was discarded, and the pellet was resuspended in binding buffer (10 mM HEPES, 150 mM NaCl, 10 mM MgCl_2_, and 1 mM KCl, pH 7.4; 2 mL per 50 mL tube). The resuspended phages were combined into one tube, phage precipitation was repeated, and phages were ultimately resuspended in 2 mL of binding buffer. Any residual bacteria were pelleted (13,500 g, 20 min), and the supernatant was transferred to a fresh tube. The solutions were incubated at 65 °C for 15 min to kill any remaining bacteria before being stored at 4 °C until further use.

### Phage Quantification

For all experiments, phages were quantified via a colony-forming unit assay. In this assay, serial dilutions of the phage solution were prepared in 2×YT media and 10 μL of each dilution was added to 90 μL of log-phase *E. coli* ER2738. Following the addition of the phage dilutions, the culture was incubated at 37 °C for 45 min and then 10 μL was spotted in triplicate onto agar selection plates containing either 100 μg/mL ampicillin and 10 μg/mL tetracycline, which were incubated at 37 °C overnight. The following day, colonies in each spot were counted, and this number was used to calculate the number of colony-forming units in the solution.

### 10mer Cyclic Library Selection

For the first round of selection, 10 μg of G17V protein was immobilized on 100 μL of streptavidin-coated magnetic beads (50% slurry, Cytiva Sera-Mag). The phage library (∼10^10^ PFU) was dissolved in binding buffer (DPBS, pH 7.5) supplemented with 1 mM TCEP, 0.25 mM PMSF, 50 μM GDP, 5 mM MgCl_2_, 0.05% Tween 20, and 1% BSA, and subsequently incubated with the G17V-coated beads at room temperature for 2 h under gentle rotation to facilitate binding. Following incubation, the beads were washed four times with binding buffer, 3 min per wash, to remove nonspecifically bound phages, with the final wash buffer retained for phage titer determination. Phages that remained bound to G17V were eluted using 100 mM glycine buffer (pH 2.2) and immediately neutralized with 1 M Tris-HCl (pH 8.0). The titer of the eluted phage fraction was determined prior to amplification. Eluted phages (100 μL) were used to infect 40 mL of log-phase *E. coli* ER2738 cells. Infected cultures were plated on ampicillin-containing agar to select positive colonies. After overnight growth at 37 °C, the resulting cultures were harvested, and plasmid DNA encoding the selected phage library was isolated. This plasmid library was subsequently transformed into electrocompetent Top10F cells containing M13KO7g3TAA and pEVOL-AcrKRS to generate the first output cyclic peptide phage library stock. The phage library was then expressed and purified as described previously to serve as input for the second round of selection. The second round followed the same binding and elution procedure as the first round, with the addition of a negative selection step. In this step, phages were preincubated with streptavidin beads lacking immobilized G17V to remove nonspecific binders prior to exposure to the target protein. Washing steps were increased to six cycles to enhance stringency and eliminate weakly bound phages. Subsequent third and fourth rounds of selection were carried out iteratively with increasing stringency. This included reducing the amount of immobilized G17V protein in a stepwise manner (10 μg in the second round, 5 μg in the third, and 2 μg in the fourth) and increasing the number and rigor of washing steps to favor high-affinity binders. After four rounds of selection, the plasmid DNA from the fourth output library was isolated and used as a template to amplify the peptide-encoding sequences. The amplified DNA was subjected to Next-Generation Sequencing (NGS) to characterize the enriched peptide sequences and assess library diversity. Multiple selection strategies were employed to minimize off-target enrichment, including competitive elution with Vav1 in round 4 to favor high-affinity binders and alternate selection on NHS-activated resin to eliminate phages with nonspecific resin interactions.

### Phage Amplification

A small aliquot (10 μL) of the phage elution was removed for quantification of phages. The remaining solution was added to an actively growing culture of *E. coli* ER2738 (OD600 = 0.5–0.6) in 20 mL 2×YT containing 10 μg/mL tetracycline for 45 min at 37 °C with rotation. After 45 min, the cells were pelleted (3750g, 15 min), resuspended in 200 mL of 2×YT containing 100 μg/mL ampicillin and 10 μg/mL tetracycline, and amplified overnight at 37 °C. The following day, phagemids were extracted from the amplified culture using a commercial plasmid purification kit. The remaining culture was then inoculated into 100 mL of fresh 2×YT containing 100 μg/mL ampicillin and 10 μg/mL tetracycline for subsequent phage expression.

### 12mer Library Expression

*E. coli* ER2738 containing the pADL-NNK12-gIII or pADL-NNN5-gIII phagemid library was cultured in 250 mL of 2×YT medium supplemented with 100 μg/mL ampicillin, 10 μg/mL tetracycline, and 1% glycerol at 37 °C with shaking. When the cultures reached an optical density at 600 nm (OD600) of 0.5–0.6, 20 mL of each culture was transferred into a sterile flask and infected with 20 μL of CM13d3 helper phage (Antibody Design Laboratories, San Diego, CA) to achieve a multiplicity of infection (MOI) greater than 5. Infection proceeded at 37 °C under agitation for 45 min to allow efficient phage propagation. Following infection, cells were pelleted by centrifugation at 3,750 g for 15 min and resuspended in 200 mL of fresh 2×YT medium containing 10 μg/mL tetracycline, 100 μg/mL ampicillin, 25 μg/mL kanamycin, and 1 mM IPTG to induce phage protein expression. Cultures were incubated at 30 °C for 18 h to enable efficient phage assembly and secretion. After induction, cultures were distributed into 50 mL centrifuge tubes, pelleted at 3,750 g for 20 min, and the supernatant containing secreted phage particles was transferred to fresh tubes for downstream processing. Phage particles were precipitated by addition of 5× precipitation buffer (20% polyethylene glycol) 8000, 2.5 M NaCl) to achieve a 1× final concentration and incubated at 4 °C for 1.5 h to facilitate complete aggregation. Precipitated phages were pelleted at 10,000 g for 30 min, and the supernatant was carefully discarded. The phage pellet was resuspended in 2 mL of binding buffer (10 mM HEPES, 150 mM NaCl, 10 mM MgCl_2_, 1 mM KCl, pH 7.4 per 50 mL tube), combined into a single tube, and the precipitation procedure was repeated to ensure maximal phage recovery and purity. Residual bacterial cells were removed by centrifugation at 13,500 g for 20 min, and the clarified supernatant was incubated at 65 °C for 15 min to inactivate any remaining bacteria. Purified phages were stored at 4 °C until further use.

### Phage Library Cyclization with CAmCBT

The purified phage library, at a concentration of 10^11^–10^12^ CFU in binding buffer, was subjected to reductive treatment to ensure complete accessibility of cysteine residues for cyclization. To this end, 1 mM tris­(2-carboxyethyl)­phosphine (TCEP) was added, and the mixture was incubated at 37 °C for 30 min. Following reduction, the library was treated with CAmCBT (10 mM stock in DMSO, 1% final DMSO), added to a final concentration of 100 μM, and the reaction was allowed to proceed for 3 h at room temperature under gentle rotation to promote efficient intramolecular cyclization. Residual reducing agent was subsequently neutralized by the addition of 10 mM oxidized glutathione, with a 30 min incubation at room temperature, ensuring complete quenching of free thiols. The resulting library, now containing cyclic CX12C-peptides, retained its full diversity and structural integrity, and was immediately suitable for downstream affinity selection assays without further purification

### 12mer Cyclic Library Selection

Streptavidin-coated magnetic beads (100 μL, 50% slurry; Cytiva Sera-Mag) were washed three times with 1 mL of binding buffer (10 mM HEPES, 150 mM NaCl, 10 mM MgCl_2_, 1 mM KCl, pH 7.4) and resuspended in 100 μL of binding buffer, then split into two aliquots. Biotinylated G17V protein (10 μg) was added to 1 mL of binding buffer in one tube (+ tube), while the other tube received an equal volume of binding buffer only (− tube). The beads/protein mixtures were incubated at room temperature with gentle rocking for 2 h to ensure immobilization. Following immobilization, the supernatant was removed, and the beads were washed three times with 1 mL of binding buffer. Subsequently, 5 × 0.25 mL of blocking buffer (binding buffer supplemented with 5% BSA and 0.5% Tween 20) was added to each tube, and the phage solution was incubated at room temperature with end-overend rotation for 30 min. The blocking buffer was then removed from the – tube, and the purified 12-mer cyclic phage library (∼10^12^ PFU) was incubated with the beads for 30 min at room temperature to perform a negative selection, depleting nonspecific binders. The supernatant from this step was transferred to the + tube and incubated for 30 min at room temperature to allow specific binding to immobilized G17V. After incubation, the supernatant was removed, and the resin was washed with wash buffer (binding buffer +0.1% Tween 20) to remove nonspecifically bound phages, performing 8 × 1 mL washes in the first round and increasing to 10 × 1 mL washes in subsequent rounds to enhance stringency. During each wash, the resin was thoroughly resuspended by pipetting, and to prevent phage adhesion to polypropylene, the resin was transferred to fresh tubes after every other wash. Bound phages were eluted with 100 μL of 50 mM glycine buffer (pH 2.2) for 15 min and immediately neutralized with 50 μL of 1 M Tris-HCl (pH 8.0). The neutralized elution was used directly to infect 40 mL of log-phase *E. coli* ER2738, and positive colonies were selected on ampicillin-containing agar to generate the first output library. Subsequent rounds incorporated negative selection against beads lacking G17V, progressively reduced amounts of immobilized G17V (10 μg, 5 μg, 2 μg, 1 μg), and iterative increases in washing stringency. Parallel selection strategies were employed, alternating streptavidin, NHS, and GSH beads, with GST-G17V immobilized on GSH beads where applicable, following identical binding, washing, elution, and amplification protocols. After four rounds of selection, plasmid DNA from the final output library was isolated and used as a template to amplify peptide-encoding sequences for Next-Generation Sequencing, enabling comprehensive analysis of enriched cyclic peptides.

### Phage Amplification

A small aliquot (10 μL) of the phage elution was removed for quantification of phages. The remaining solution was added to an actively growing culture of *E. coli* ER2738 (OD600 = 0.5–0.6) in 20 mL 2×YT containing 10 μg/mL tetracycline for 45 min at 37 °C with rotation. After 45 min, the cells were pelleted (3750g, 15 min), resuspended in 200 mL of 2×YT containing 100 μg/mL ampicillin and 10 μg/mL tetracycline, and amplified overnight at 37 °C. The following day, phagemids were extracted from the amplified culture using a commercial plasmid purification kit. The remaining culture was then inoculated into 100 mL of fresh 2×YT containing 100 μg/mL ampicillin and 10 μg/mL tetracycline for subsequent phage expression.

### Illumina Sequencing Selected Phage Libraries

A four-step PCR cycle was used to amplify the library region out of the original phagemid library using primers NGS-F1 and NGS-R1, as has been previously described.^46^ The amplicons were purified and extracted from a 3% agarose gel according to a GenCatch gel extraction kit, then indices were attached using a subsequent PCR with NGS-i7 and NGS-i5 primers. To identify the rounds of selection, each round contained a unique combination of i7 or i5 indices. The PCR products were purified using a GenCatch gel extraction kit and submitted to the Genomics and Bioinformatics center at Texas A&M University for sequencing on an Illumina iSeq (4 M Reads, 2 × 150) Sequences were analyzed for enrichment in R, and all scripts are available in the Supporting Information.

### Biolayer Interferometry Assays

BLI experiments were performed using an Octet R8 system (Sartorius) with Streptavidin (SA) biosensors. All experiments were carried out in assay buffer consisting of DPBS (pH 7.5) supplemented with 1 mM TCEP, 0.25 mM PMSF, 50 μM GDP, and 5 mM MgCl_2_. Biotinylated RhoA G17V was immobilized onto SA sensors at 20 μg/mL for 10 min, followed by quenching in blocking buffer (assay buffer +0.1% BSA) for 3 min. Lyophilized peptides Z1, Z2, and Z3 were dissolved directly in assay buffer, whereas RhoA2, RhoA3, RhoA5, Z4, Z5, and Z6 were dissolved in DMSO and diluted in assay buffer to achieve a final DMSO concentration of 3%. Sensors were sequentially dipped into assay buffer for baseline equilibration (5 min), peptide solutions for association (210 s), and back into assay buffer for dissociation (5 min). For multiple sample measurements, SA sensors were regenerated using 10 mM glycine (pH 2.2) for 30 s, followed by equilibration in assay buffer for 60 s. All measurements were conducted at 30 °C. Data were processed in Octet Analysis Studio 13 software by double referencing to ligand-only and protein-only wells and aligning the curves to the start of association. Global kinetic fitting was performed using a 1:1 binding model.

The wild-type protein was expressed (Figure S49) for testing for the selectivity of Z1 peptide using BLI. To assess selectivity against non-Rho GTPases, proteins from two distinct subfamilies-KRAS (Ras subfamily) and RAB7A (Rab subfamily)-were procured from commercial sources: KRAS from Abcam (catalog no. ab309960) and RAB7A from Creative Biomart (catalog no. RAB7A-194H).

### Peptide Synthesis

Initial sequences of peptides (until N-terminal cysteine) were synthesized on a low-loading ProTide rink amide resin (CEM #R002) using an automated Liberty Blue Peptide Synthesizer with an HT12 attachment. All derivatives were standard derivatives for Fmoc peptide synthesis. Fmoc-amino acids were deprotected using 20% piperidine in DMF with a two-step microwave cycle: (1) 75 °C, 15 s (2) 90 °C, 50 s. Residues were then coupled with 0.125 M N,N′-diisopropylcarbodiimide (DIC) and 0.25 M Oxyma using CEM’s standard microwave coupling cycle: (1) 75 °C, 15 s (2) 90 °C, 110 s. For peptides that contained CAmCBT, following the synthesis of peptides on the Liberty Blue, the linker was coupled by hand in a two-step process. First, Fmoc-Aluc (15 mg, 1.2 equiv) was coupled to the N-terminus using 1-[bis­(dimethylamino)-methylene]-1H-1,2,3-triazolo­[4,5-*b*]­pyridinium 3-oxide hexafluorophosphate (HATU) (152 mg, 4 equiv) and DIEA (72 μL, 4 equiv) in DMF (1 mL) and reacted for 16 h at room temperature. Following this, the chloroacetyl moiety was added. Chloroacetyl chloride (8 μL, 4 equiv) was added to the resin at 4 °C containing DIEA (72 μL, 4 equiv) and incubated for 3 h at room temperature. The chloroacetyl chloride coupling was repeated once before cleavage. The peptides were cleaved from the resin by agitating for 3h in 3 mL of 92.5:2.5:2.5:2.5 TFA/H2O/dioxa-1,8-octane-dithiol (DODT)/triisopropyl silane (TIPS). The products were then filtered, and peptides were precipitated out of the filtrate using cold ether. The precipitates were collected by centrifugation, washed again with cold ether, and then lyophilized. The crude products were then dissolved in 1 mL of DMF containing 4 equiv of DIPEA and cyclized for 2 h at 42 °C. The peptides were purified by reversed-phase HPLC (acetonitrile in water with 0.1% formic acid) using either a Discovery BIO wide pore C18–5 column (25 cm × 10 mm, Millipore-Sigma #568230-U) or a Shimpack GIS C18 (20 × 250 mm2, 10 μm, Shimadzu). Following purification, fractions were analyzed using high-resolution mass spectrometry. Corresponding masses for synthesized peptides are listed in Table S1. Peptide binders identified from the 10-mer selection were synthesized by solid-phase peptide synthesis with site-specific incorporation of the unnatural amino acid N-(((9H-fluoren-9-yl)­methoxy)­carbonyl)-S-(3-(allyloxy)-3-oxopropyl)-l-cysteine (Figure S50) at the N-terminus and an Aloc-protected lysine, (Fmoc-Lys­(Aloc)–OH) residue at the C-terminus (synthetic scheme and characterization provided below). Following completion of linear chain assembly, the peptide–resin was suspended in dichloromethane (3 mL), treated with Pd­(PPh_3_)_4_ (15 mg), and sparged with nitrogen for 1 min to remove dissolved oxygen. Phenylsilane (125 μL) was then added as an allyl scavenger, and Aloc deprotection was carried out for 15 min at room temperature with gentle agitation. The deprotection step was repeated once to ensure complete removal of the Aloc group, after which the resin was extensively washed with dichloromethane and DMF. On-resin macrocyclization was performed by activation of the carboxylate using HOBt (22 mg) and PyBOP (65 mg) in the presence of *N*-methylmorpholine (28 μL) in DMF (4 mL). The reaction was allowed to proceed overnight at room temperature and was repeated for a second night to drive the cyclization to completion. Following cyclization, the peptides were cleaved from the resin by agitation for 3 h in a cleavage cocktail composed of 92.5:2.5:2.5:2.5 TFA/H_2_O/dioxa-1,8-octane-dithiol/triisopropylsilane. The cleavage solution was filtered to remove the resin, and the peptides were precipitated from the filtrate using cold diethyl ether. The resulting precipitates were collected by centrifugation, washed with additional cold ether, and lyophilized. Crude peptides were purified by reversed-phase high-performance liquid chromatography using a gradient of acetonitrile in water containing 0.1% formic acid, employing either a Discovery BIO wide-pore C18 column (25 cm × 10 mm, 5 μm) or a Shimpack GIS C18 column (20 × 250 mm^2^, 10 μm). Purified fractions were analyzed and confirmed by high-resolution mass spectrometry.

### Monoclonal Phage Expression

Plasmids encoding cyclic peptides RhoA1–RhoA5 were cotransformed into *E. coli* Top10F’ cells harboring M13KO7g3TAA and pEVOL-ClodF-AcrK. Single colonies were selected and inoculated into 2 mL 2×YT medium and cultured overnight at 37 °C with shaking. The overnight culture was subsequently transferred into 200 mL fresh 2×YT medium and grown to mid log phase. Expression of monoclonal phage particles was induced by supplementation with 1 mM IPTG, 2 mg/mL arabinose, 5 mM nicotinamide, and 5 mM AcrK. Following induction, phage particles were purified from the culture supernatant and used directly for downstream phage ELISA assays.

### Phage ELISA

G17V-biotin (200 ng per well) was diluted in ELISA buffer (DPBS, pH 7.5, 1 mM TCEP, 0.25 mM PMSF, 50 μM GDP, 5 mM MgCl_2_) and immobilized onto ELISA plate wells (100 μL/well) by overnight incubation at 4 °C. Wells were then blocked with 3% BSA in ELISA buffer (200 μL/well) at room temperature for 2 h. Following blocking, wells were washed twice with ELISA buffer, and serial dilutions of the purified monoclonal phage were added (100 μL/well) and incubated at room temperature for 2 h to allow binding. Unbound phages were removed by washing six times with washing buffer (ELISA buffer containing 0.05% Tween-20) on a shaker at room temperature, 2 min per wash, ensuring complete removal of wash solution after each cycle. Bound phages were detected using HRP-conjugated anti-M13 antibody, followed by addition of 100 μL TMB substrate per well. The reaction was allowed to develop at room temperature for 10 min, generating a blue chromogenic product, and absorbance was measured at 650 nm using a microplate reader.

## Results and Discussions

Rho family GTPases have long been considered among the most refractory intracellular proteins for chemical targeting because their globular architecture offers almost no deep binding pockets beyond the nucleotide-binding site, while the high cellular concentration of GTP/GDP and the tight binding affinity of GTPases for nucleotides render the site essentially undruggable.^29–31^ Attempts to inhibit activation pathways, for instance by blocking interactions between RhoA and its guanine-nucleotide exchange factors (GEFs) have yielded small-molecule inhibitors such as Rhosin,^32,33^ which binds near Trp58 on RhoA and inhibits GEF-mediated activation with submicromolar affinity. However, these inhibitors remain rare, often lack sufficient potency or cellular efficacy,^34^ and none to date has targeted pathogenic mutant forms of RhoA directly or reached the milestone of FDA approval. There remains a critical need for alternative molecular modalities capable of engaging shallow or flat protein surfaces, especially those of mutant GTPases, with high affinity and specificity. Macrocyclic peptides have emerged as a powerful solution in this regard. Their constrained ring structure reduces the entropic penalty upon binding, enhances proteolytic resistance, and affords a larger, adaptable contact surface than typical small molecules,^20,35–44^ enabling them to recognize and block protein–protein interactions (PPIs). Recent successes targeting challenging intracellular proteins including members of the Ras GTPase family further demonstrate the feasibility of this strategy. Cyclic peptide and macrocycle campaigns have been validated against similarly challenging targets for instance, mutant K-Ras (G12D), where cyclic peptides discovered via mRNA-display bind preferentially to the GTP bound state and disrupt effector interactions.^45^

Motivated by these successes, we constructed a genetically encoded macrocyclic peptide library tailored for phage display. Our design leverages an on-phage cyclization chemistry previously developed in our lab,^46^ an N-terminal cysteine residue undergoes a proximity-driven Michael addition onto a genetically encoded acryloyl-lysine (AcrK), yielding a stable thioether macrocycle. This configuration ensures structural rigidity while preserving the genotype vital for library selection and allows sampling of conformationally constrained ring scaffolds capable of engaging shallow or transient epitopes. The framework aligns with broader practices in peptide macrocycle drug discovery, which emphasize conformation preorganization, stable cyclization, and high throughput display platforms for library screening. This approach enables site-specific cyclization to occur directly on the phage under aqueous conditions, while remaining fully compatible with established phage display protocols, and yields conformationally constrained macrocyclic peptides with defined topology. While this design provides structural rigidity and precise control over macrocycle formation, the incorporation of ncAAs inherently limits overall library diversity due to constraints in encoding and incorporation efficiency.

### Phage Biopanning Using the AcrK-Mediated Cyclic Library

RhoA G17V containing an N-terminal GST-Tag was recombinantly expressed in *E. coli* together with BirA, a biotin ligase, for site-specific biotinylation at the AviTag (Figure S1). The GST tag was cleaved using HRV3C protease prior to phage biopanning. Selections were carried out using an AcrK-cyclized 10-mer peptide library displayed on M13 phage to identify binders to RhoA G17V . Two different selection strategies were performed to aid in the identification of hit peptides over nonspecific binders (Figure 2a). In one method, streptavidin beads were used to immobilize the protein in each round, while the other consisted in alternating the immobilization chemistry using NHS coupling and streptavidin beads between rounds. Prior to each positive selection step, phage libraries were incubated with unloaded beads for negative selection to eliminate clones exhibiting nonspecific bead binding. Increasing stringency was applied across rounds through extended washing, increased detergent concentration, and decreased target protein loading. After four rounds of biopanning with each technique, the enriched phages from the NHS selection (R3S2, Figure 2a) and the two streptavidin elutions (R4S1 and R4S2, Figure 2a) were analyzed via next generation sequencing. All peptide sequences identified and their abundances can be found in Table S1 of the supplementary data. Diversity analysis of the eluted phages indicated substantial enrichment of sequences after four rounds of selection (Figure 2b). For the streptavidin-only selection, 3 sequences made up nearly 90% of all peptides identified through the NGS analysis, whereas the alternative selection strategy resulted in slightly higher diversity (4 peptides comprising of 65% of the sequences). To give a better understanding of the enrichment of peptide sequences and diversity of the final pools, we performed UMAP analysis to cluster the top 100 peptides of each pool according to sequence composition and physical properties. In this selection, there was limited formation of peptide “islands” in the UMAP plot of the three pools (Figure 2c). Additionally, there was essentially no overlap between the pools from the different selection strategies, with only one peptide being common to all three, making up less than 0.01% of all the sequences (Figure 2d). We reasoned that these results likely indicated that the enrichment observed in the streptavidin-only selection was likely due to nonspecific binders or parasitic clones. There was significant overlap between the NHS and Streptavidin pools in the alternative selection strategy, with all the top clones in round 4 being shared between the two samples (Figure 2e). As such, the most abundant sequence in the streptavidin-only selection (RhoA1) and four of the most abundant from the NHS-elution (RhoA2-5) were selected for initial testing (Figure 2f). Peptide-encoding phagemids were transformed into *E. coli* Top10F′ carrying M13KO7-g3TAA and pEVOL-CloDF-AcKRS, cultured from 5 mL overnight cultures into 200 mL, and induced with IPTG, arabinose, nicotinamide, and AcrK to generate monoclonal phage displaying the cyclized peptides. Phage ELISA demonstrated that among the enriched clones, RhoA5 and RhoA3 exhibited strong and selective binding to immobilized RhoA G17V , whereas RhoA2 showed intermediate binding with the mutant (Figure S2). All of these hit peptides were only found in the alternative resin selection strategy. RhoA2 (CFSLFEWDDDGAcrK), RhoA3 (CWNWLENSVFGAcrK), and RhoA5 (CWRVFIWGQGPAcrK) were selected for chemical synthesis and further characterization.

**Figure 2:**
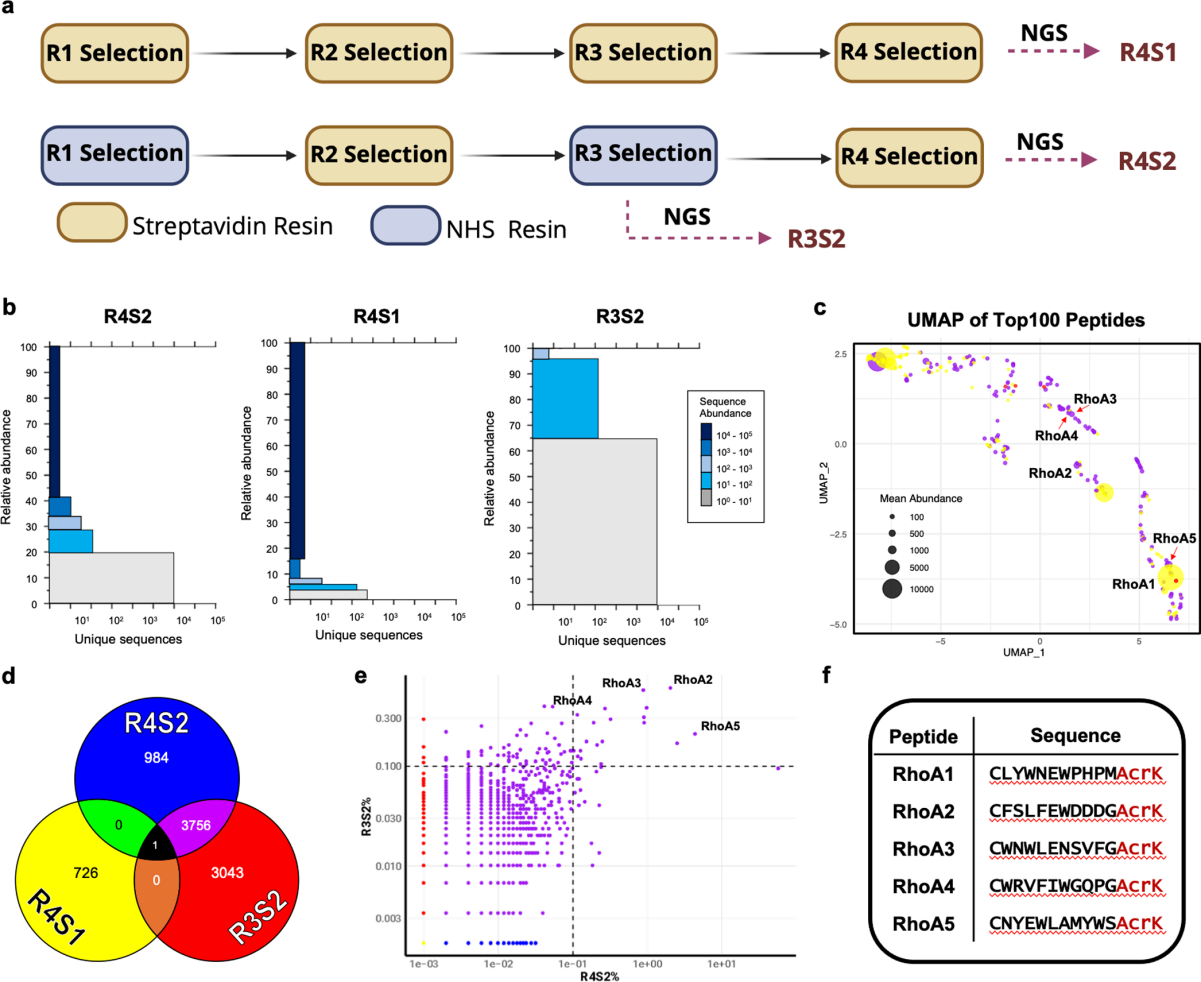
(a) Multiple selection strategies were applied to the 10-mer cyclic library to minimize nonspecific binding toward the resin scaffolds during the selection against RhoA-G17V. (b) Illumina next-generation sequencing of enriched peptide hits from different selection strategies indicated successful enrichment after four rounds of selection. (c) UMAP analysis of the top100 peptides by clustering based on hydrophobicity, peptide sequence, aliphatic composition, and charge. Colors correspond to the overlapping sequence pools found in panel d. (d) Venn diagram of the unique sequences found in each enriched selection pool. (e) Scatter plot of the abundances of sequences found in R4S2 or R3S2 selection pools. Colors for each point correspond to the venn diagram plot. (f) Peptide sequences that were ultimately tested for activity toward RhoA using an ELISA assay.

### Phage Biopanning Using 12mer Cyclic Library

To potentially overcome the limited chemical diversity and moderate enrichment of a consensus sequence observed with the initial 10-mer macrocyclic library, we also employed a larger 12-mer cyclic peptide library to target the oncogenic RhoA G17V variant. This library leveraged a previously developed regioselective CAmCBT organic linker that reacts with an N-terminal cysteine via a cyanobenzothiazole condensation reaction and an internal cysteine through nucleophilic substitution to a chloroacetamide.^45^ The library consisted of 12 randomized amino acids flanked by an N-terminal and an internal cysteine, cyclized postexpression using the CAmCBT linker. To address the limited enrichment observed with the 10-mer library, a 12-mer library was selected to better accommodate the binding requirements of the RhoA G17V surface, which presents a relatively shallow and extended interface. The increased peptide length enables broader surface engagement and improved contact formation. In addition, the CAmCBT-based cyclization was specifically chosen for its ability to generate macrocycles with distinct geometry and flexibility thereby enabling access to binding modes not readily sampled by the initial library. Expression in *Escherichia coli* yielded over 1 × 10^12^ phages, which were subsequently cyclized prior to biopanning, ensuring library diversity and chemical integrity of the macrocycles.

To maximize the probability of isolating high-affinity ligands, the library was subjected to three complementary parallel biopanning strategies, each employing alternating immobilization chemistries to minimize selection bias (Figure 3a). After four rounds of enrichment, outputs were analyzed by next-generation sequencing, and top peptides where identified through a variety of analyses of the sequencing pools. Sequences identified from these selections and their abundances can be found in Table S2. Unlike in the initial selection strategy, there was considerable overlap between the different samples, potentially indicating the successful enrichment of peptides that interact with RhoA regardless of resin type (Figure 3b). 3853 unique peptide sequences were common to all selections, making up 16.9% of the sequencing pool. As expected, overlap was increased between the selections that used glutathione for immobilization (GSH or GSH/NHS) compared to their overlap with the streptavidin-NHS selection (Strep/NHS). Whereas 8822 sequences were uniquely shared between the two selections containing glutathione immobilization steps, only 2814 and 2432 sequences were uniquely shared between the Strep/NHS strategy and GSH or GSH/NHS, respectively (Figure 3b). This likely indicates that some of the peptides identified in the GSH pools interact with the interface or glutathione resin, rather than RhoA itself. Enrichment profiles of the sequencing pools indicated a substantial number of peptides with high abundance for each sample, albeit lower in abundance than the prior selection strategy (Figure 3c). We believe the high stringency of alternating the immobilization scaffold resulted in decreased enrichment of top sequences, but nonetheless each selection resulted in significantly enriched peptides to characterize. UMAP analysis of the top peptides resulted in clustering of two major islands, with the most abundant peptides being localized in one cluster (Figure 3d). Through comparative scatter plots of peptide abundance profiles, we noted all peptides with high abundances (>0.1% in final round) were found in all three samples (Figure 3e), further indicating a successful selection. Among all sequences, peptide Z1 (CDSWFFWDEHTDDC) consistently ranked first across all three selection strategies, reflecting strong enrichment and potential high-affinity interaction with RhoA G17V. The additional peptide sequences identified for characterization are shown in Figure 3f. Z2 (CGYLDNWWEVGYSC), Z3 (CTLLDPWPWADSEC), and Z4 (CEFDMFLWGEEEAC), and Z6 (CQLLDEWWPESDEC) were highly enriched in all selection pools, whereas Z5 (CYSRIHLWVGVVSC), predominantly in GSH selection strategy (Figure 3f).

**Figure 3:**
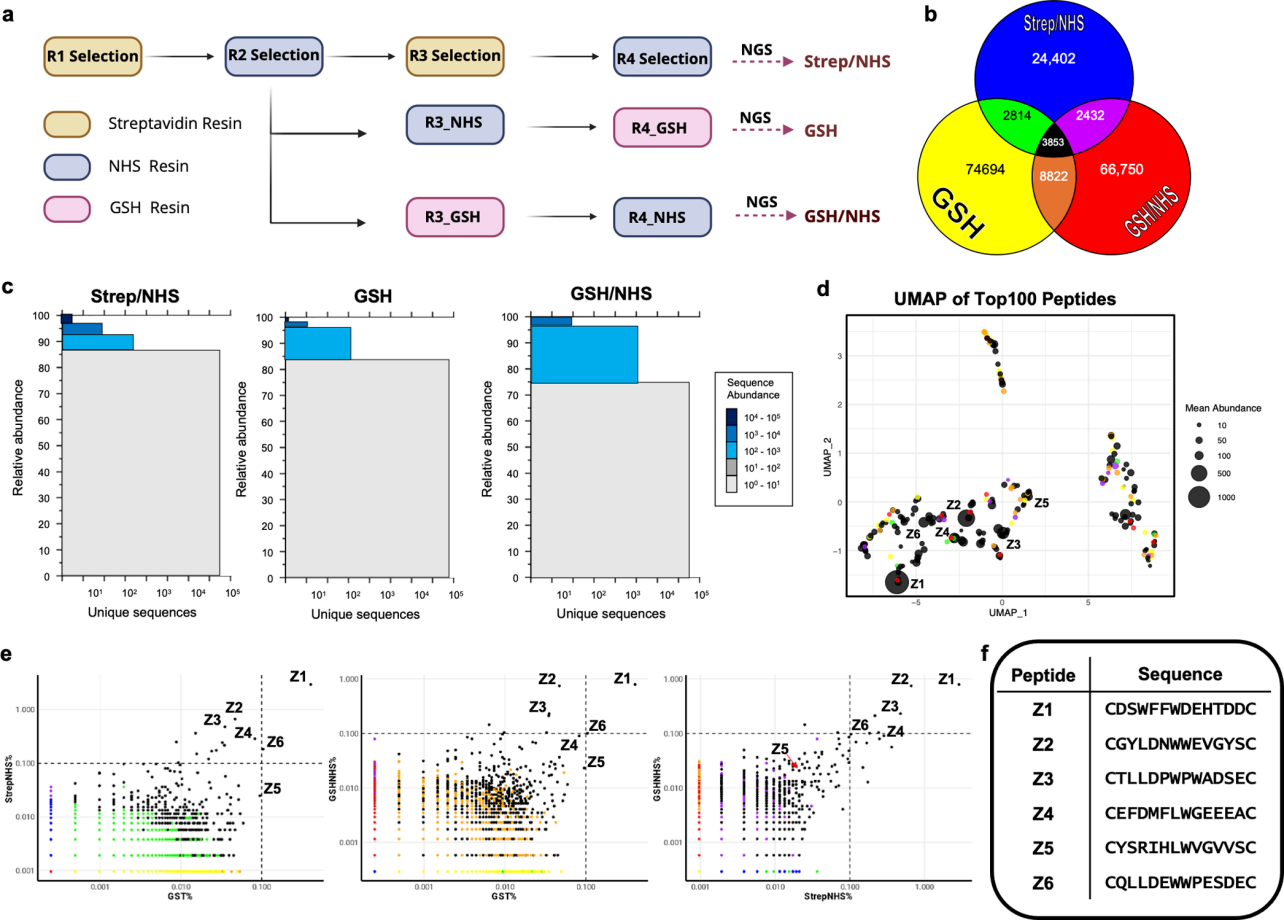
(a) Multiple selection strategies were applied to the 12-mer cyclic library to minimize nonspecific binding toward the resin scaffolds during the selection against RhoA-G17V. Multiple selection strategies were applied to the 12-mer cyclic library to minimize nonspecific binding. (b) Venn diagram of the unique sequences found in each enriched selection pool. (c) Illumina next-generation sequencing of enriched peptide hits from different selection strategies indicated successful enrichment after four rounds of selection. (d) UMAP analysis of the top100 peptides by clustering based on hydrophobicity, peptide sequence, aliphatic composition, and charge. (e) Scatter plot of the abundances of sequences found in three different selection pools. Colors for each point correspond to the venn diagram plot. (f) Top peptide hits identified from the 12mer library for characterization.

### Characterization of Identified Peptides for Interactions with RhoA G17V

Having used phage display to identify possible hit peptide ligands for RhoA G17V from two different macrocyclic peptide libraries, we then looked to further characterize the peptide hits for their affinities toward. All peptides were synthesized by solid-phase peptide synthesis, cyclized, purified and verified by high-resolution mass spectrometry prior to characterization (Figures S3–S16). Their binding affinities toward RhoA G17V were then quantified using biolayer interferometry through immobilization of biotinylated RhoA G17V onto streptavidin biosensors. Consistent with the phage ELISA studies and enrichment analysis, peptides from the CX10AcrK phage library demonstrated only modest affinities, with dissociation constants of 6.32 μM for RhoA2, 27.33 μM for RhoA3, and 10.69 μM for RhoA5. The peptides identified through the CAmCBT selection, on the other hand, demonstrated high enhanced affinities for RhoA. Peptide Z1 exhibited a dissociation constant (K_D_) of 136 nM (Figure 4), establishing it as the first reported submicromolar macrocyclic peptide binder for the RhoA G17V mutant. To further assess the specificity of peptide Z1, its binding was evaluated against wild-type RhoA and representative non-Rho small GTPases, KRAS and RAB7A. Z1 exhibited 4.7-fold higher affinity for the RhoA G17V mutant compared to wild-type RhoA, indicating preferential recognition of the mutant form. In addition, the peptide showed 11.7-fold and 12-fold selectivity over KRAS and RAB7A, respectively. Despite belonging to distinct subfamilies, KRAS and RAB7A share a conserved GTP-binding fold with RhoA, including similar nucleotide-binding motifs and switch regions, which may account for the low level of residual binding observed. Together, these results support selective recognition of the RhoA G17V mutant while maintaining limited cross-reactivity with other small GTPases (Figures S17–S19). Z2, Z4, and Z6 displayed moderate binding with K_D_ values of 5.54 μM, 1.73 μM and 3.83 μM, respectively, whereas Z3 and Z5 exhibited no measurable binding. (Figures S20–S21). Importantly, the linear counterpart of Z1 failed to show any detectable binding (Figure S22), underscoring the critical role of macrocyclization in maintaining structural rigidity and preorganized geometry necessary for high-affinity engagement. These observations collectively demonstrate that the CAmCBT-mediated macrocycle confers both conformational stability and target selectivity, facilitating effective interactions with the shallow, highly conserved surface of RhoA G17V.

**Figure 4:**
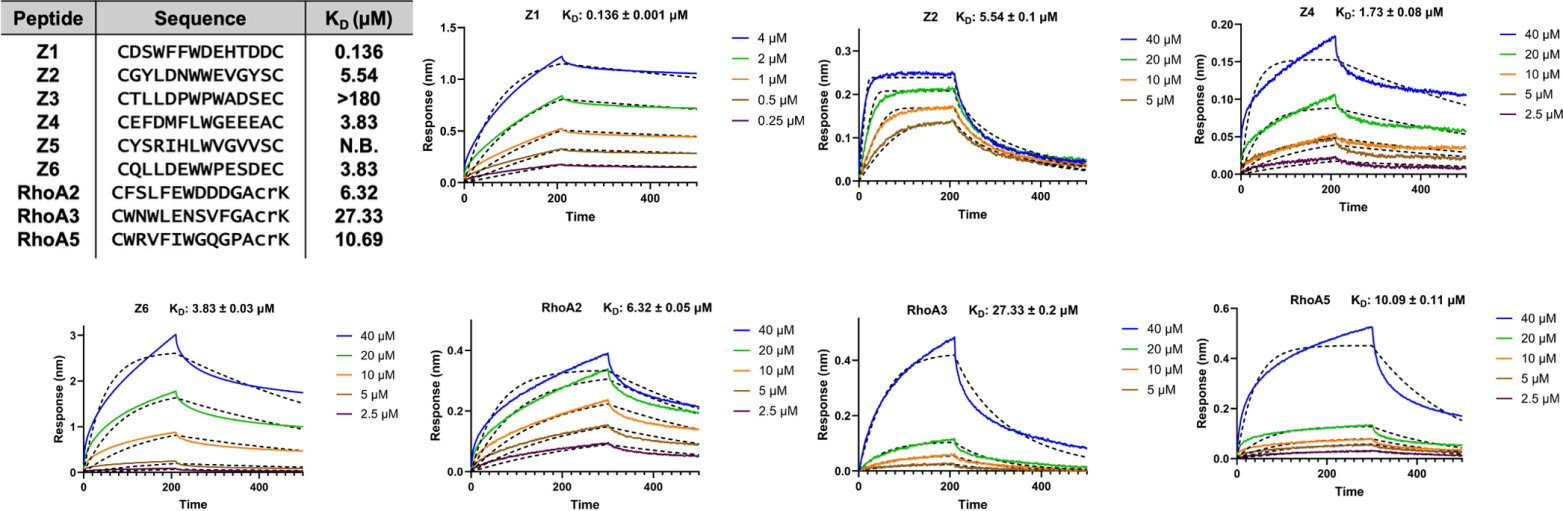
Top peptide hits identified from the 12mer library for characterization. Binding affinities of each peptide hit toward recombinant RhoA G17V were quantified by biolayer interferometry (BLI), with reported K_D_ values presented as mean ± standard deviation (SD) from three independent measurements.

### Structural Characterization of the Peptide-RhoA Complex Using Molecular Docking and Molecular Dynamics

Following the initial validation of peptide Z1 as a potent binder for RhoA G17V, we then looked to further investigate the interaction through computational studies. To shed light on the structural basis of the Z1 peptide within the binding site of oncogenic RhoA G17V mutant, we conducted an initial blind docking step to the G17V protein, followed by a local docking step for conformational optimization (Figure 5a), both performed by GNINA 1.3. To obtain the protein structure of this mutation, ColabFold was utilized to generate five.pdb structures^47^ of RhoA G17V, from which the best model was chosen for the downstream computational work (pLDDT = 92.6, pTM = 0.886) (Figures S23–S25). The initial blind docking was set to generate up to 100 conformations using flexible docking method. These conformations were then subjected to RMSD-based clustering (cutoff at 20 Å on the whole macrocycle). Within one cluster, only the conformation with the most negative binding affinity (kcal/mol) was retained for the optimization step (local docking) to refine the binding mode of the peptide in complex with the protein. The docking grid box position was automatically centered on the position of the representatives, while the grid box size was dynamically scaled to fully encompass both the peptide and surrounding binding residues. Eventually, eight different representatives were selected with binding affinities ranging from −7.45 to −9.00 kcal/mol (Figure 5b). Interestingly, there was a seemingly general correlation observed between the binding affinity and cluster size: the more tightly the representatives could bind to the protein, the more members the cluster could hold. These representatives distributed extensively around the RhoA G17V protein, ensuring the unbiased assessment of where the peptide could potentially bind, and therefore enhancing the efficiency of the molecular dynamics (MD) simulation in the subsequent stage.

**Figure 5:**
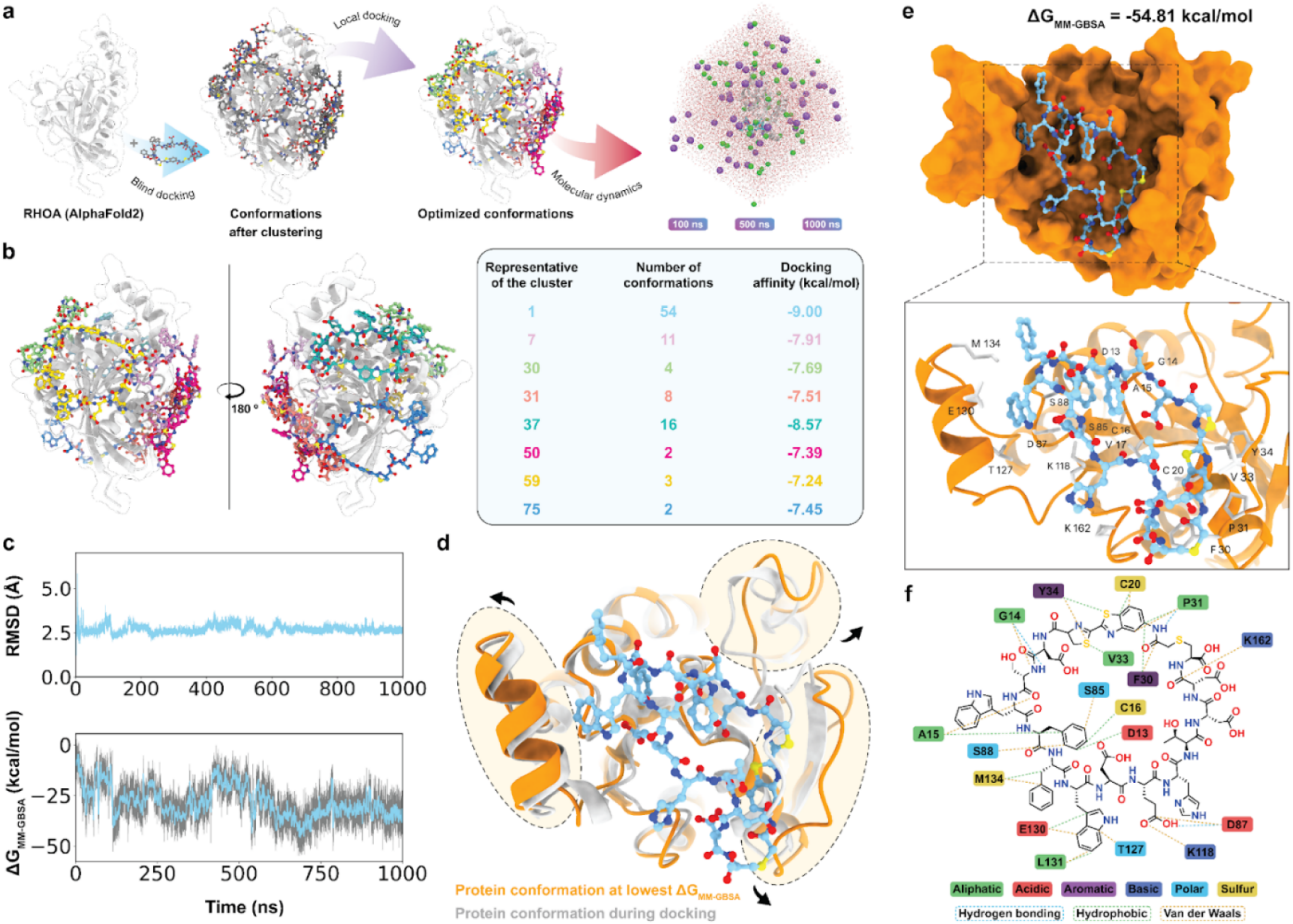
Computational workflow performed on RhoA G17V and the peptide. (a) Workflow of the computational work: blind docking for unbiased binding site determination, local docking for binding mode optimization, and 100 ns MD runs, which could adaptively be extended to 500 and 1000 ns depending on whether the binding equilibrium was ever reached or not. (b) Docking results of eight representative conformations with their positional distribution, docking score and the number of members in each cluster after the clustering step (the colors in the table corresponded to the color of the conformers). (c) 1000 ns MD results of conformation 1 - the most stable complex among eight protein-macrocycle complexes, including RMSD plot of the whole complex and the average free energy of binding (ΔG_MM_-G_BSA_) every 1 ns (sky blue) during 1000 ns MD run against the free energy each frame (gray). (d) Binding site of conformation 1 within RhoA was corresponding to the GDP binding site and the conformational shift of RhoA apo form (light gray) to the bound state (orange) upon the binding process of RhoA to the peptide (sky blue) as observed in the MD trajectory. (e) 3D interaction diagram between the peptide and the residues in the binding site at the MD frame with the most negative free energy of binding ΔG_MM_-G_BSA_ (−54.71 kcal/mol). (f) 2D interaction plot between the peptide and the residues within the binding site at the most negative binding energy of the complex (adapted from 2D diagrams generated by ProLIF).

All the representative conformations were initially proceeded to a 100 ns MD run, where only conformation 1 and conformation 59 showed binding equilibrium as indicated by stable RMSD profiles (Figure S26), and were therefore fed to a subsequent 500 ns MD run. Between them, only conformation 1 could maintain long-term conformational stability inside the binding site of RhoA (Figure S27), and its MD simulation was extended to 1000 ns for in-depth analysis. It adopted a favorable free energy of binding ΔG_MM_-G_BSA_ at −28.46 ± 16.05 kcal/mol on average during the 1000 ns MD simulation and low RMSD_complex_ variation (Figures 5c). Notably, this conformation 1 also exhibited the most negative docking score, and its cluster accounted for more than half of all generated conformations, suggesting a dominant binding mode. In addition, it demonstrated overlap with the native GDP-binding pocket of RhoA and coincided with the mutation site at G17V, reinforcing the biological plausibility and implications of this binding orientation. During the MD trajectory, RhoA experienced a conformational shift to open more space to accommodate the peptide in the binding cleft (Figure 5d).

With the presence of linker, the peptide showed a decent level of rigidity in the binding site of RhoA G17V as shown in the RMSD_peptide_ values of these structures within 1000 ns of the MD simulation (Figure S28). Extracting the frame where the free energy of binding ΔG_MM_-G_BSA_ became the most negative, the peptide mostly interacted with RhoA via the hydrophobic and van der Waals interactions with the residues F4, F5 and W6 in the macrocyclic structure. There was one key hydrogen bonding interaction between the acidic side chain of E8 in the peptide to D87 residue of the protein (Figure 5e, f). As such, these four residues are predicted to play strong roles in the binding affinity. The linker itself also demonstrated various interaction types, highlighting the significant role this specific fragment played in the binding event. During this 1000 ns MD simulation, the isopropyl side chain of V17 could frequently interact with the peptide via hydrophobic interactions, therefore confirming the significance of this mutation specifically in the interaction with the peptide (Figure S29). In wildtype RhoA, glycine occupies this position and lacks a lipophilic side chain, thereby precluding the formation of hydrophobic interactions with the peptide Z1 at this site, explaining why there was no binding in RhoA-Z1 peptide complex. To further confirm the hypothesis, 1000 ns simulation for RhoA-Z1 was performed, in which the protein RhoA-G17V was mutated back to the wildtype using PyRosetta and conformation 1 of Z1 (the most stable conformation during the 1000 ns MD simulation) was reused. Toward the end of the MD simulation, Z1 dislocated significantly out of the original binding site as seen in the superimposition between the initial pose and the final pose of Z1 in complex with RhoA alongside the continuous increase in RMSD_complex_ (Figure S30 a and b). Average free energy of binding ΔG_MM_-G_BSA_ of Z1 in wildtype RhoA was higher than in RhoA-G17V at −24.04 ± 8.51 kcal/mol during the 1000 ns MD simulation and tended to move toward 0 kcal/mol during course of the simulation (Figure S30 a), while 2D interaction fingerprint showed the loss of most interactions after 800 ns (Figure S30 c). These computational results are in agreement with experimental binding data, where Z1 demonstrated 4.7-fold higher affinity for RhoA G17V relative to wild-type RhoA, supporting the role of the valine side chain in stabilizing the peptide–protein interaction.

### Experimental Validation of Key Residues via Alanine Scanning

To better understand and validate the predicted interactions from the computational studies, we then looked to experimentally characterize alanine mutants of the Z1 peptide (Figures S31–S42) for their binding to RhoA G17V through biolayer interferometry (Figure 6b–g). With the terminal cysteines excluded due to macrocyclization, the functional core begins at D1, and the substitutions W3A, F4A, F5A, W6A, E8A, and D12A each caused substantial (>9-fold) reductions in affinity (Figure 6a), indicating that these residues make key contributions to binding within a spatially confined interaction region. The most severe penalties occurred within the contiguous aromatic cluster W3, F4, F5, W6, and this pattern aligns strongly with the molecular dynamics studies that consistently positioned these side chains in a buried hydrophobic pocket overlapping with the GDP-binding region of RhoA G17V. MD simulations demonstrated various van der Waals and hydrophobic contacts between this aromatic block and the surrounding nonpolar residues of RhoA, interactions that collectively formed the dominant stabilizing component of the peptide–protein interface. The marked sensitivity of E8A further supports the predicted binding pose, in the MD-derived complex, E8 forms a directional hydrogen bond and electrostatic contact with RhoA D87, likely serving as a key orientational anchor that complements the hydrophobic core by restricting peptide mobility and maintaining the packing geometry needed for deep pocket insertion. Similarly, the >9-fold loss observed with D12A could be attributed to the electrostatic interaction between D12 and K162 on RhoA G17V, as suggested by the 2D fingerprint interaction during the 1000 ns MD simulation (Figure S29). The agreement between alanine scanning results and computational modeling supports a consistent binding mode for the peptide, with the predicted orientation aligning with outcomes from molecular dynamics simulations. By contrast, mutations causing only mild (2–4-fold) (Figures S43–S48) changes, such as those near the N-terminal and central polar positions, likely affect secondary structural or solvation roles without contributing significantly to the protein-peptide interactions (as seen in Figure 5f). Taken together, the alanine-scanning results and computational analysis define a coherent mechanistic picture in which high-affinity binding relies on synergistic hydrophobic packing by the W/F/W cluster, strategic electrostatic stabilization by E8 and D12, and a macrocyclic scaffold that preserves the geometry required for both interactions, offering strong experimental validation of the predicted binding mode and a clear structural rationale for future peptide optimization.

**Figure 6:**
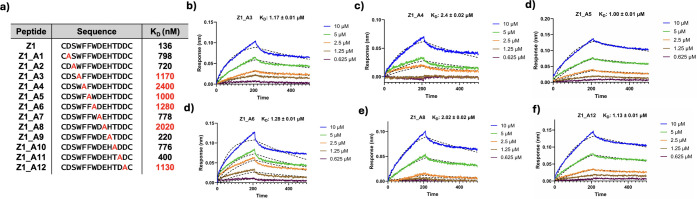
(a) Alanine scanning of macrocyclic peptide variants. (b–g) Binding affinities of each alanine-substituted variant toward recombinant RHOA G17V were quantified by biolayer interferometry (BLI), with reported K_D_ values presented as mean ± standard deviation (SD) from three independent measurements. Residues whose substitution resulted in >9-fold loss of affinity are highlighted in red, indicating positions essential for high-affinity recognition.

## Conclusions

RhoA G17V represents a highly challenging intracellular target due to its rigid GTPase fold, lack of deep druggable pockets, and high-affinity nucleotide binding, which hinder conventional small-molecule approaches. This oncogenic variant drives over 70% of angioimmunoblastic T-cell lymphoma (AITL) cases possibly through tight association with Vav1, promoting its phosphorylation at Tyr174 and hyperactivation of TCR signaling. Our study establishes, for the first time, a systematic strategy to target RhoA G17V using macrocyclic peptides, combining complementary phage display libraries, computational modeling, and alanine-scanning mutagenesis to achieve high-affinity and functionally active binders.

Selections from a 10-mer AcrK-cyclized library yielded moderate-affinity ligands, including RhoA2 (K_D_ = 6.32 μM), RhoA3 (K_D_ = 27.33 μM), and RhoA5 (K_D_ = 10.69 μM). Recognizing the limitations of chemical diversity and cyclization efficiency in the 10-mer library, we deployed a 12-mer CAmCBT-mediated cyclic library with over 10^9^ unique sequences. This library produced several high-affinity binders, including Z1 (K_D_ = 136 nM), Z2 (K_D_ = 5.54 μM), Z4 (K_D_ = 1.73 μM) and Z2 (K_D_ = 3.83 μM). The remarkable enhancement of affinity in Z1 attests to the advantage of expanded library length, regioselective cyclization, and maintenance of structural diversity in capturing high-affinity interactions with shallow protein surfaces. Importantly, the linear counterpart of Z1 exhibited no detectable binding, confirming the critical role of macrocyclization in stabilizing the preorganized conformation necessary for target engagement.

Through an integrative docking and molecular dynamics (MD) workflow, we structurally characterized the binding of a macrocyclic peptide to the oncogenic RhoA G17V mutant. The peptide demonstrated a relatively rigid macrocyclic backbone supported by linker moieties having key residues like F4, F5, W6 and E8 in the peptide. Furthermore, RhoA underwent conformational rearrangements upon peptide binding, adopting an open-bound state that could dynamically fit the macrocyclic peptide. Taken together, these results provide a coherent structural model for the peptide-RhoA G17V complex formation. The findings suggest that the peptide preferentially targets the GDP-binding site, stabilizes through a network of hydrophobic and hydrogen-bonding interactions, and induces local conformational adaptation of RhoA. This study not only establishes a mechanistic foundation for peptide recognition of RhoA G17V but also offers a computational framework for guiding future optimization of peptide-based inhibitors against oncogenic RhoA mutants.

This integrated workflow, leveraging complementary phage display libraries, high-stringency selection, structural modeling, and residue-level validation establishes a generalizable platform for discovering potent cyclic peptide inhibitors against challenging mutant GTPases. The work not only demonstrates the feasibility of targeting a previously undruggable oncogenic mutant but also provides quantitative, structural, and mechanistic insights that will guide future optimization. Moving forward, Z1 and related high-affinity peptides can be further refined for improved cell permeability, metabolic stability, and intracellular delivery, enabling functional modulation of RhoA G17V signaling in cellular contexts. The comprehensive data set from micro and nanomolar binders, computational mapping, and alanine scanning provides a robust foundation for advancing peptide-based therapeutics for AITL and potentially other diseases driven by pathogenic GTPase mutants. Notably, Z1 and related high-affinity macrocyclic peptides can be optimized for improved cell permeability and intracellular delivery for direct modulation of RhoA G17V -driven oncogenic signaling. Importantly, mutant-selective binders also created new opportunities for proximity-induced degradation for RhoA G17V, which set the stage for new therapeutic avenues for AITL and other immunotherapy driven by pathogenic GTPase mutations.

## Supplementary Material



## Notes

Additional experimental details, additional sequencing analysis, characterization of peptides, BLI sensograms and R scripts (PDF)
