# Supplementary material for: Phage Display Driven Identification and Computational Mapping of Macrocyclic Peptides Targeting RhoA G17V
Source: Biochemistry. 2026 Apr 15;65(9):1465–77. doi: 10.1021/acs.biochem.6c00058 (PMC13151067; doi:10.1021/acs.biochem.6c00058)
Supplement: Supplementary file 1 [file bi6c00058_si_001.pdf]

Supplementary Information

Phage Display Driven Identification and Computational Mapping of Macrocyclic Peptides Targeting RhoA G17V

Sebin Abraham[a], Chaoyang Zhu[a], Lai Hoang Son Le[a], Yugendar R. Alugubelli[a], Tatsuki Nonomura[b], Yun Huang[b], J. Trae Hampton[a]*, Yubin Zhou[b]*, and Wenshe Ray Liu[a,b,c,d,e]*

[a]Texas A&M Drug Discovery Center and Department of Chemistry, Texas A&M University, College Station, TX 77843, USA

[b]Institute of Biosciences and Technology and Department of Translational Medical Sciences, College of Medicine, Texas A&M University, Houston, TX 77030, USA

[c]Department of Biochemistry and Biophysics, Texas A&M University, College Station, TX 77843, USA

[d]Department of Cell Biology and Genetics, College of Medicine, Texas A&M University, College Station, TX 77843, USA

[e]Department of Pharmaceutical Sciences, Texas A&M University, College Station, TX 77843, USA

*Correspondence should be addressed to J. Trae Hampton (jhampton1@tamu.edu), Yubin Zhou (yubinzhou@tamu.edu), and Wenshe Ray Liu (wsliu2007@tamu.edu)

Primers List

1) G17V F: TTGGTGACGGCGCGTGCGGGAAGACCTGCCTGCTGAT

2) G17V R: ATCAGCAGGCAGGTCTTCCCGCACGCGCCGTCACCAA

3) Vav1 F: GGAATTCCATATGGAGCCGGTTAGCATG

4) Vav1 R: CCGCTCGAGGGTTTCGTTGTCACGTTT

5) NGS-F1:
5’TCGTCGGCAGCGTCAGATGTGTATAAGAGACAGGCCCAGCCGGCCATG3’

6) NGS-R1:
5’GTCTCGTGGGCTCGGAGATGTGTATAAGAGACAGCGGCCGCTTTCGCCGC3’

7) NGS-i7: 5’CAAGCAGAAGACGGCATACGAGAT[i7] GTCTCGTGGGCTCGG3’

8) NGS-i5: 5’AATGATACGGCGACCACCGAGATCTACAC[i5] TCGTCGGCAGCGTC3’

A)

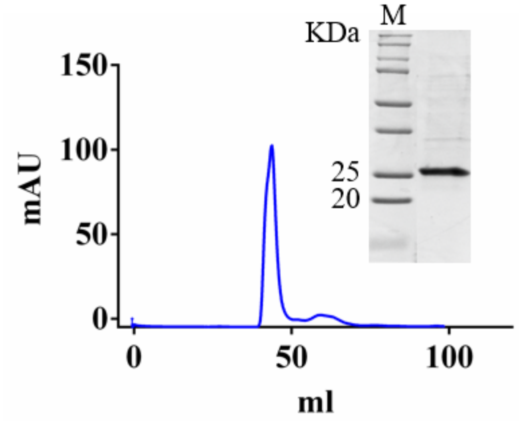

Figure S1: Expression of RhoA G17V Protein (expected size 24.9 KDa after GST Cleavage using HRV 3C Protease)

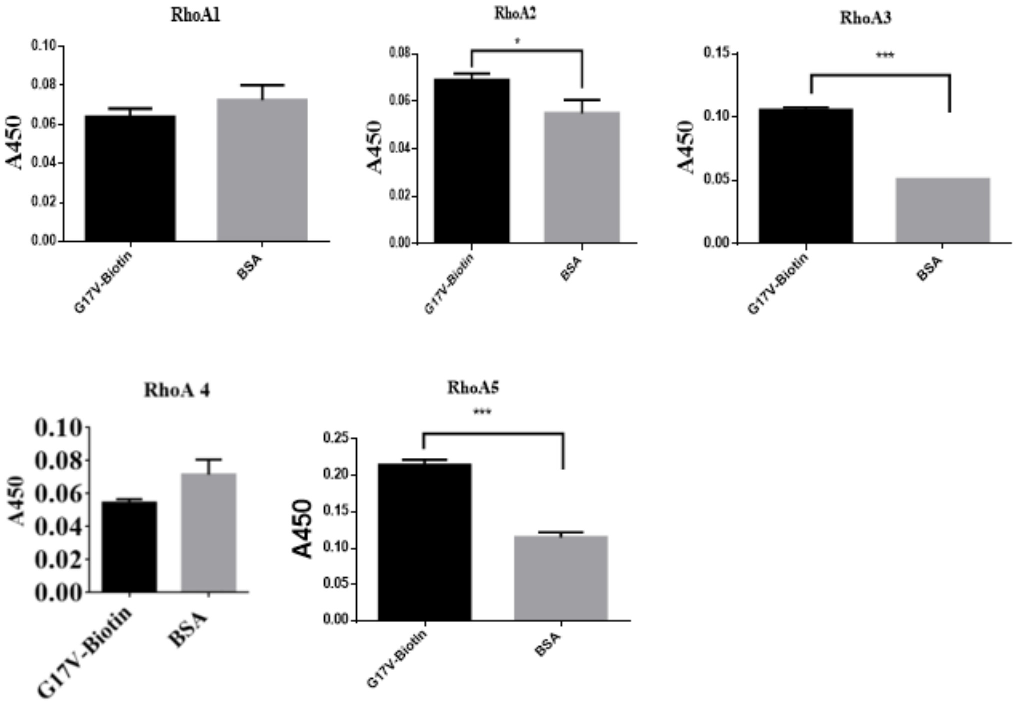

Figure S2: Phage ELSIA of selected cyclic peptides. 200 ng G17V-biotin was immobilized on the ELISA plate wells, and incubated with 10^8 pfu purified monoclonal phage. The HRP conjugated anti-M13 phage was used to detect the binding phage. BSA was used as a negative control.

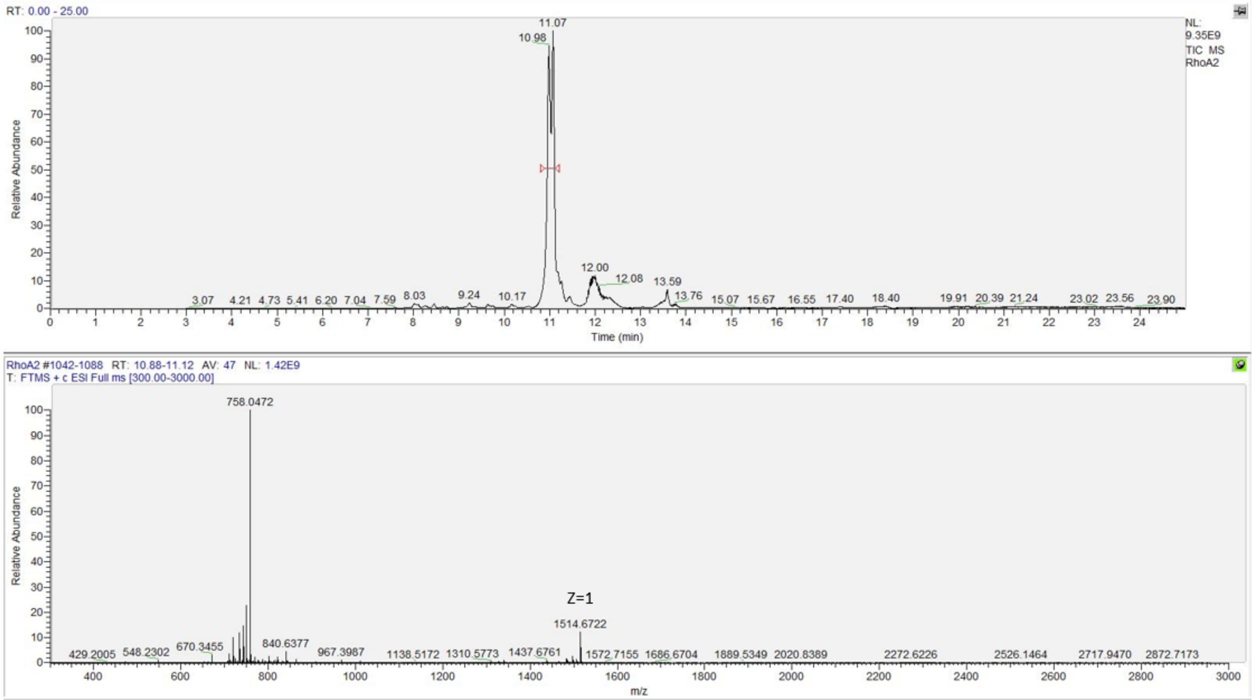

Figure S3: LC-MS Data for RhoA2. The TIC chromatogram is shown on top and the extracted masses from the peak are below.

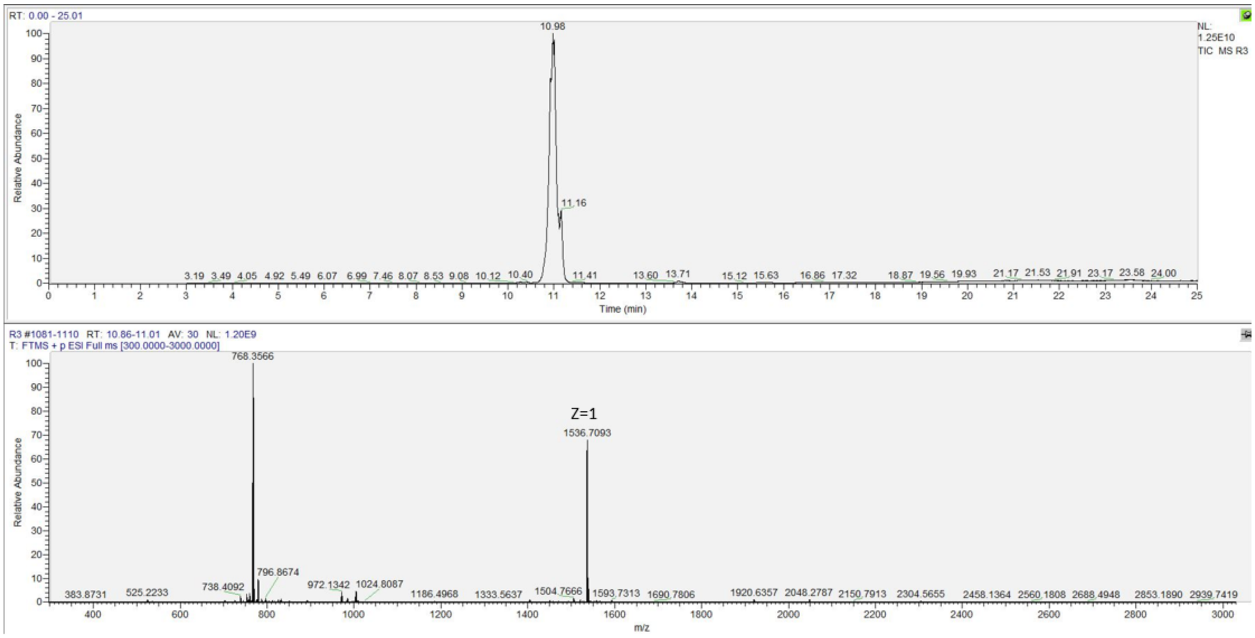

Figure S4: LC-MS Data for RhoA3. The TIC chromatogram is shown on top and the extracted masses from the peak are below.

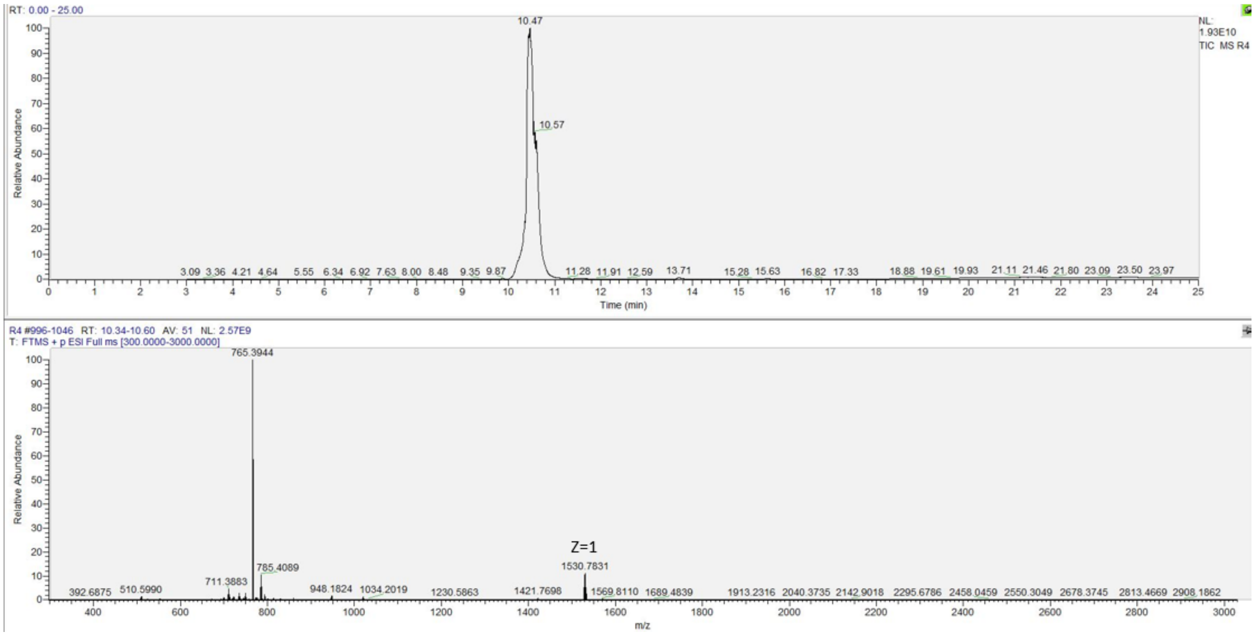

Figure S5: LC-MS Data for RhoA5. The TIC chromatogram is shown on top and the extracted masses from the peak are below

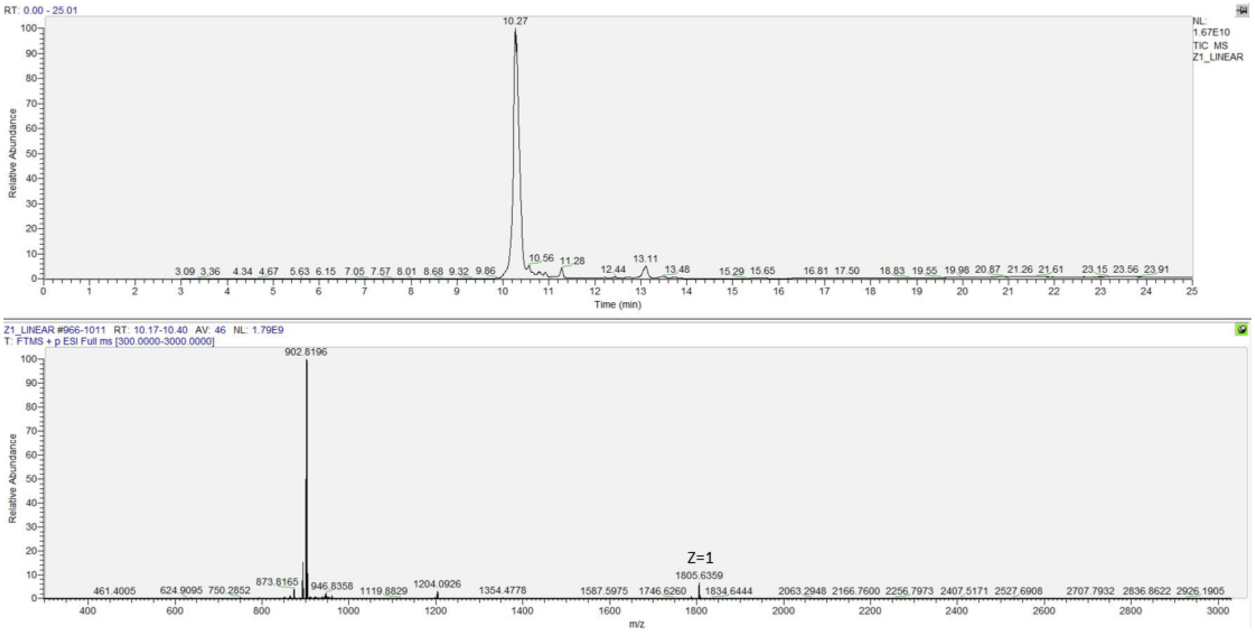

Figure S10: LC-MS Data for Z1-LINEAR. The TIC chromatogram is shown on top and the extracted masses from the peak are below.

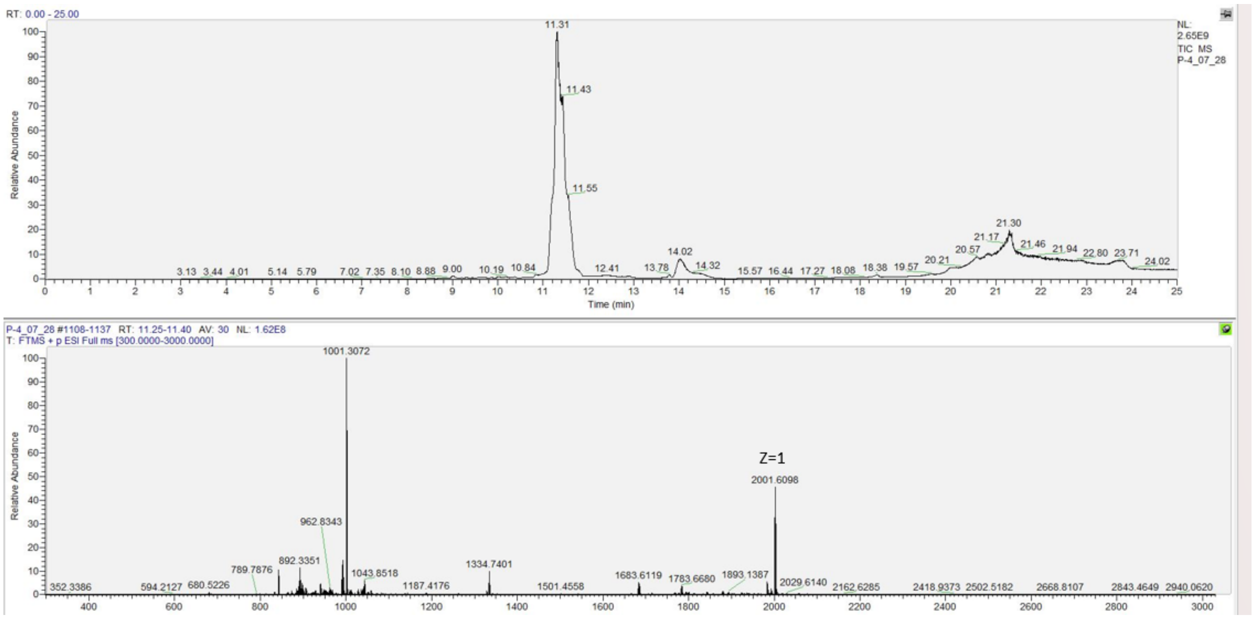

Figure S11: LC-MS Data for Z1-CYCLIC. The TIC chromatogram is shown on top and the extracted masses from the peak are below.

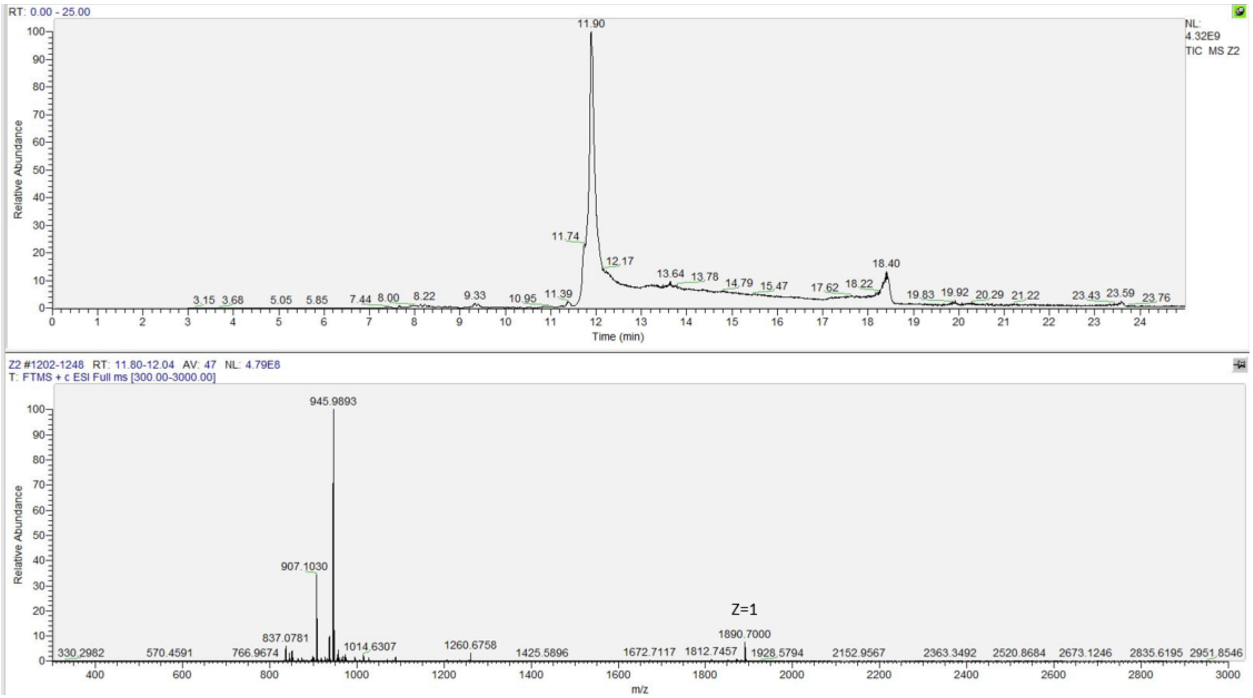

Figure S12: LC-MS Data for Z2. The TIC chromatogram is shown on top and the extracted masses from the peak are below

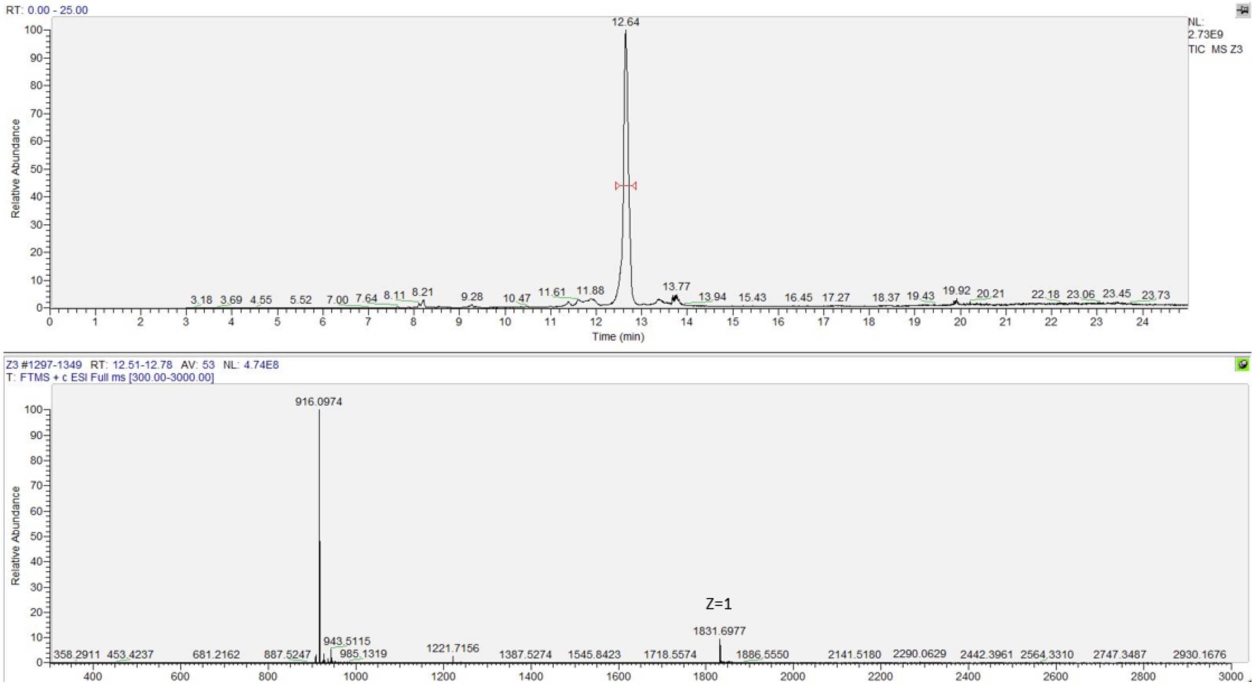

Figure S13: LC-MS Data for Z3 The TIC chromatogram is shown on top and the extracted masses from the peak are below

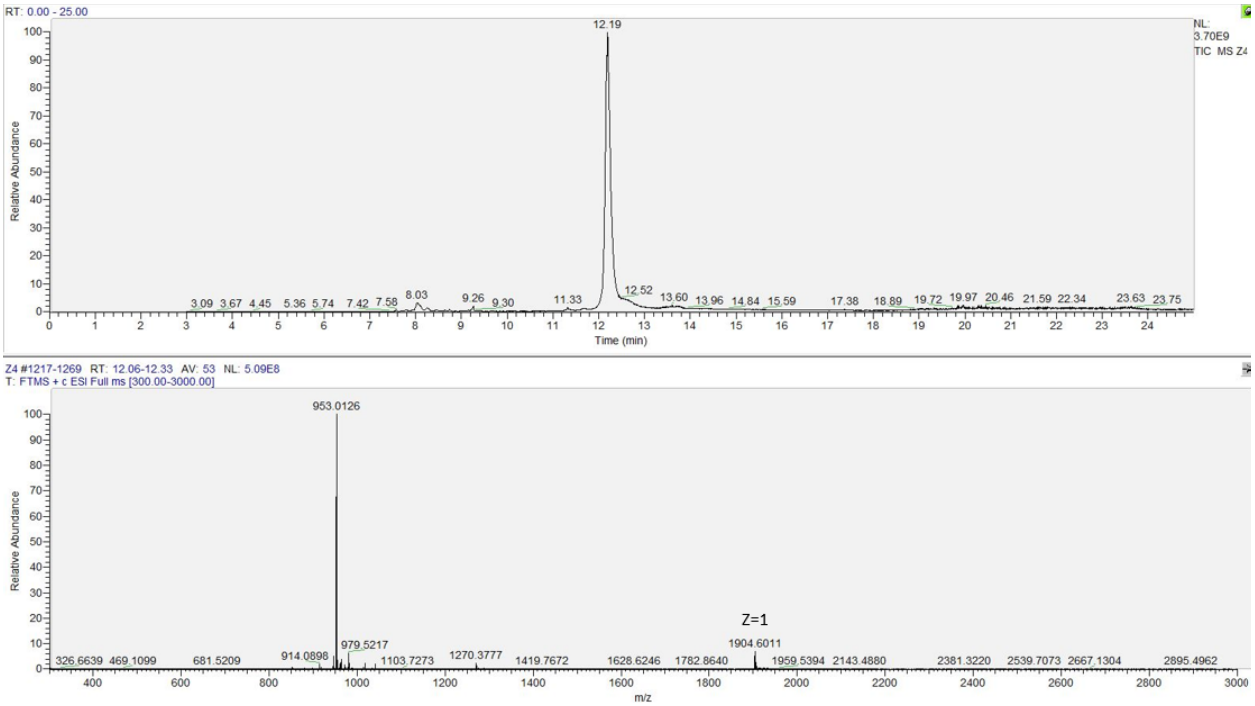

Figure S14: LC-MS Data for Z4 The TIC chromatogram is shown on top and the extracted masses from the peak are below

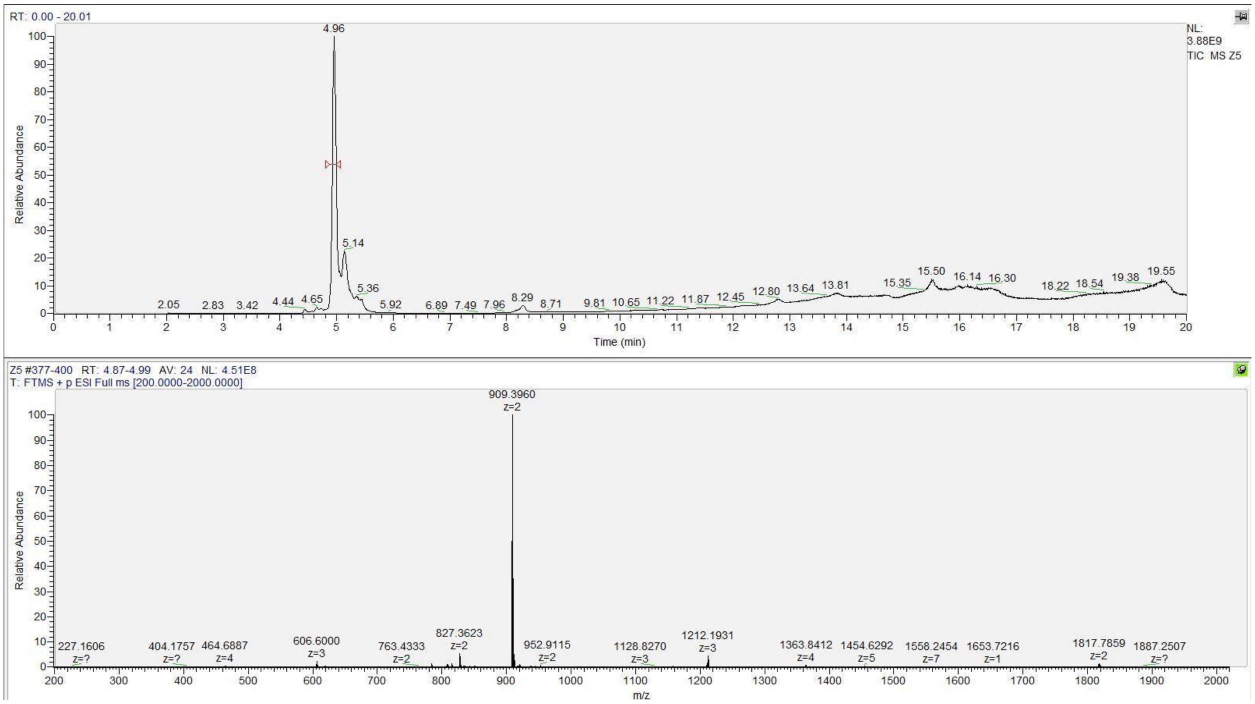

Figure S15: LC-MS Data for Z5 The TIC chromatogram is shown on top and the extracted masses from the peak are below

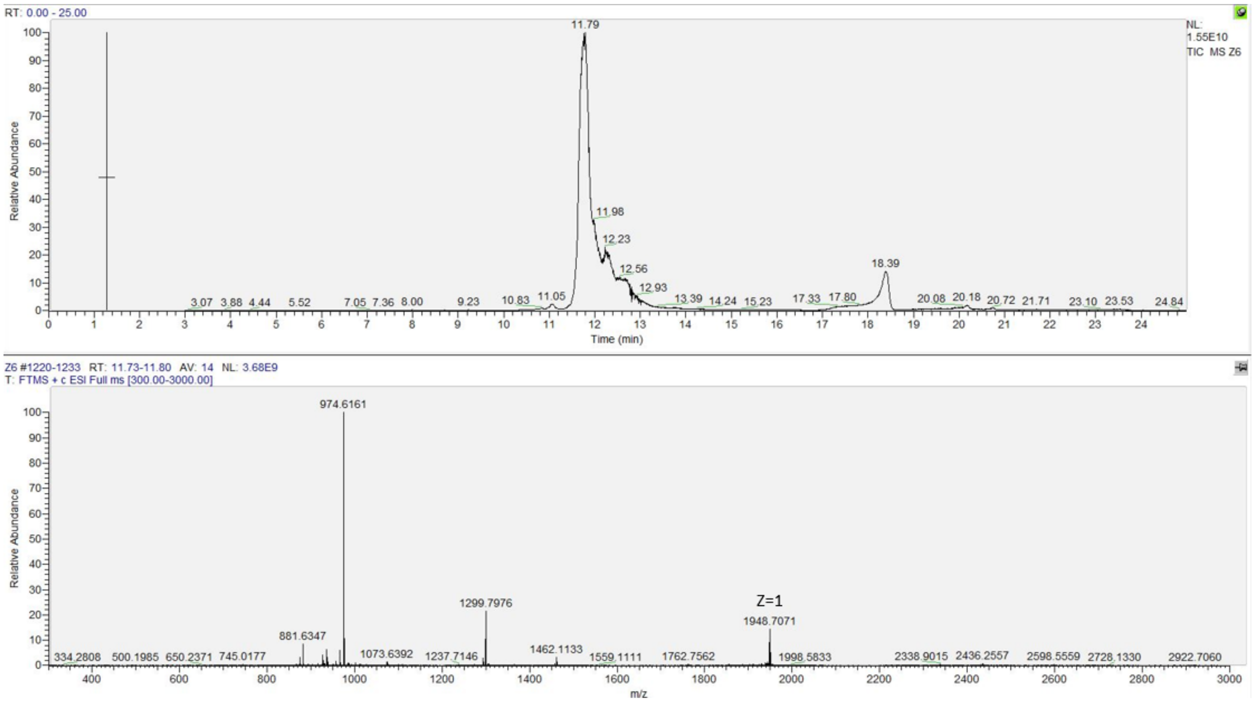

Figure S16: LC-MS Data for Z6 The TIC chromatogram is shown on top and the extracted masses from the peak are below

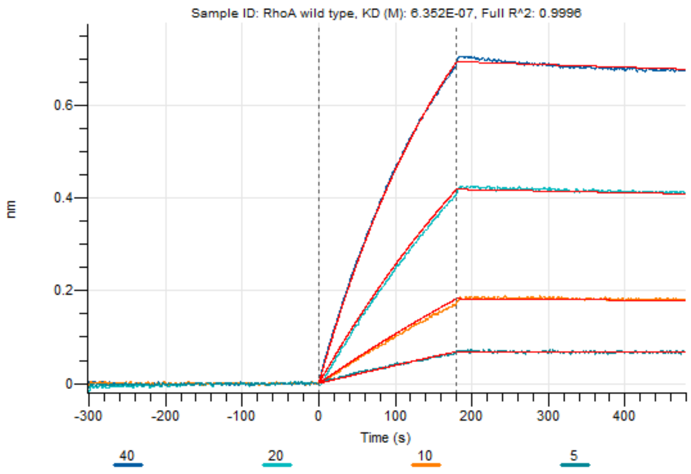

Figure S17: BLI analysis of Z1 demonstrated a 4.7-fold selectivity, with binding affinities of 653 nM for wild-type RhoA and 136 nM for the mutant.

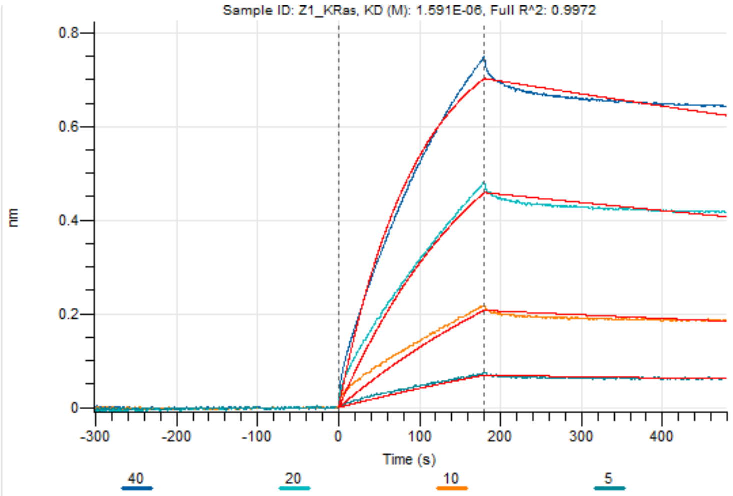

Figure S18: BLI analysis of Z1 demonstrated a 11.7-fold selectivity, with binding affinities of 1591 nM for KRAS and 136 nM for RhoA G17V.

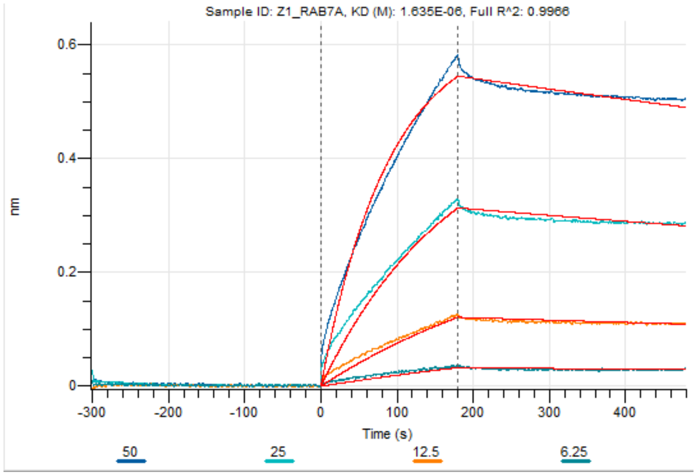

Figure S19: BLI analysis of Z1 demonstrated a 11.7-fold selectivity, with binding affinities of 1635 nM for RAB7A and 136 nM for RhoA G17V.

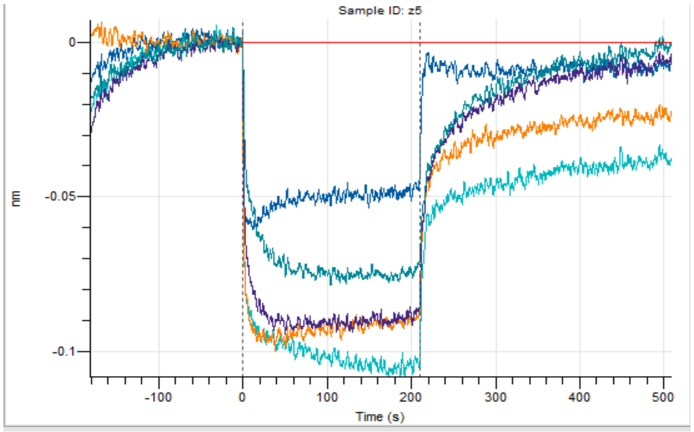

Figure S20: BLI studies of Z5 show no binding against RhoA G17V

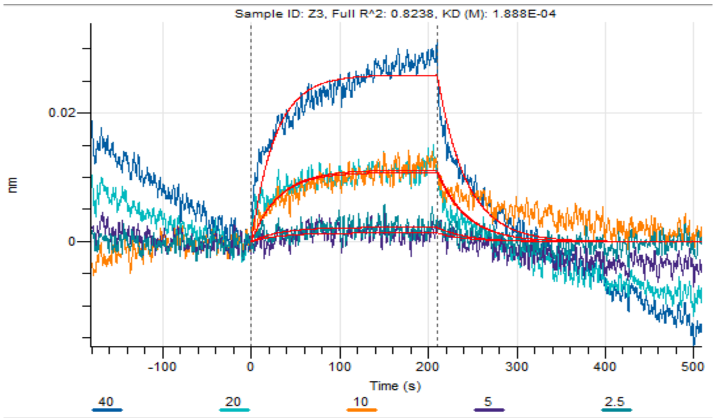

Figure S21: BLI studies of Z3 showing weak binding (> 180μM) against RhoA G17V

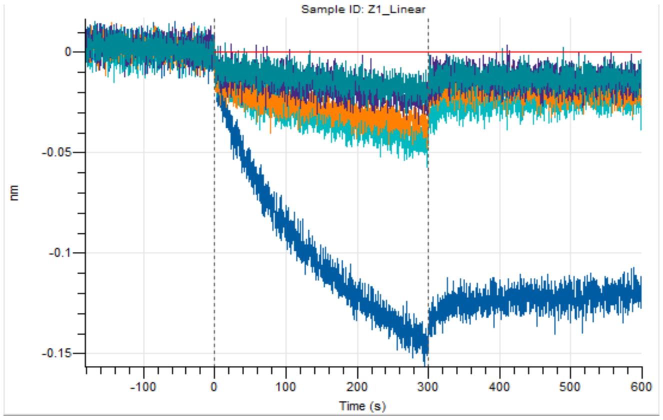

Figure S22: BLI studies of Z1 (Linear) showing no significant binding against RhoA G17V

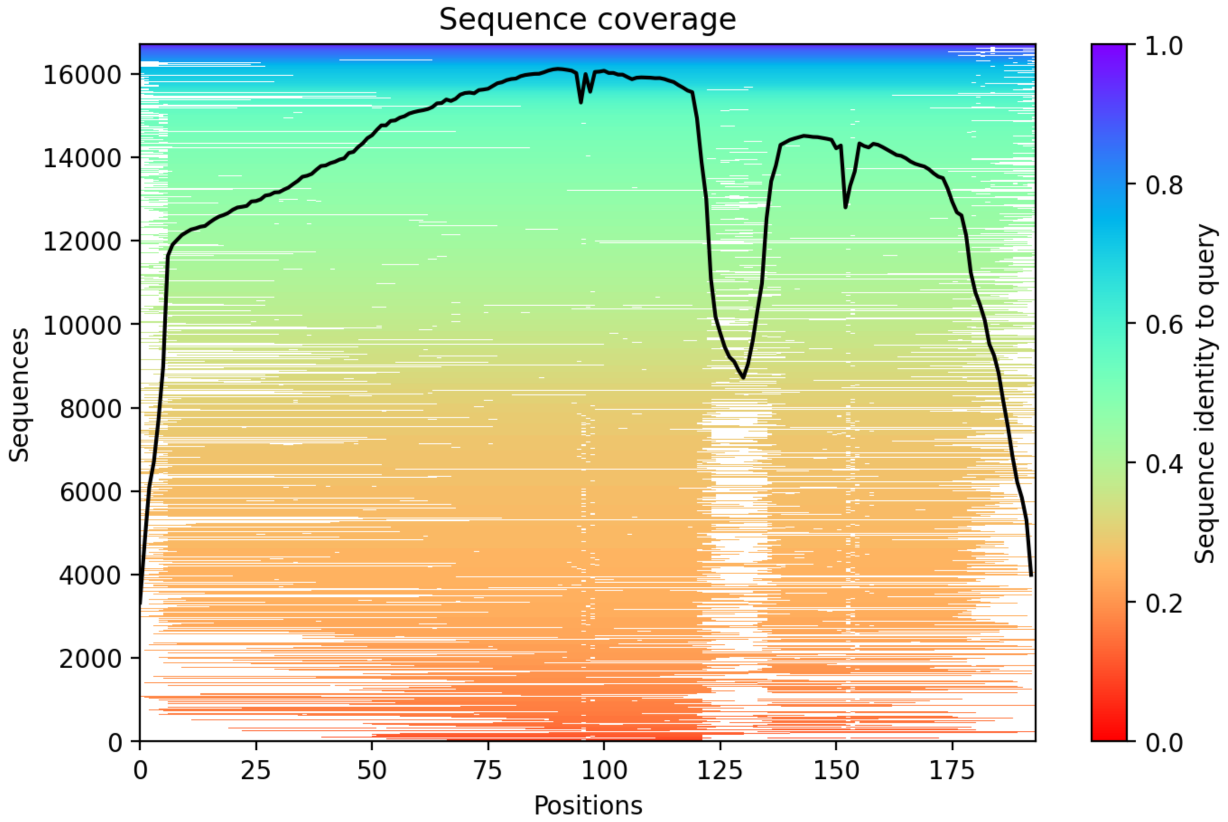

Figure S23: Sequence coverage of RHOA G17V protein that has corresponding sequences found in the Multiple Sequence Alignment (MSA) generated by the MMseqs2 search

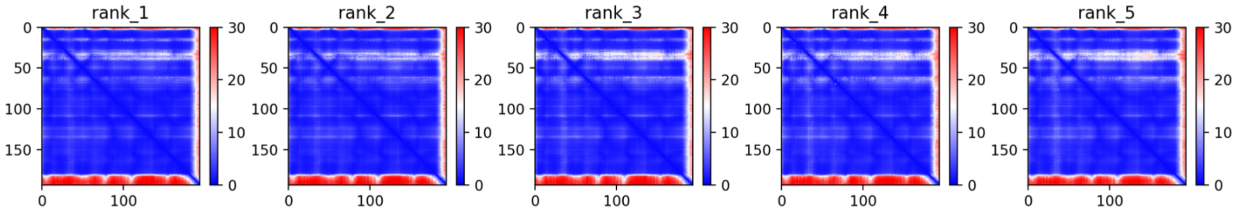

Figure S 24: PAE score of top 5 conformations of RHOA G17V generated by ColabFold

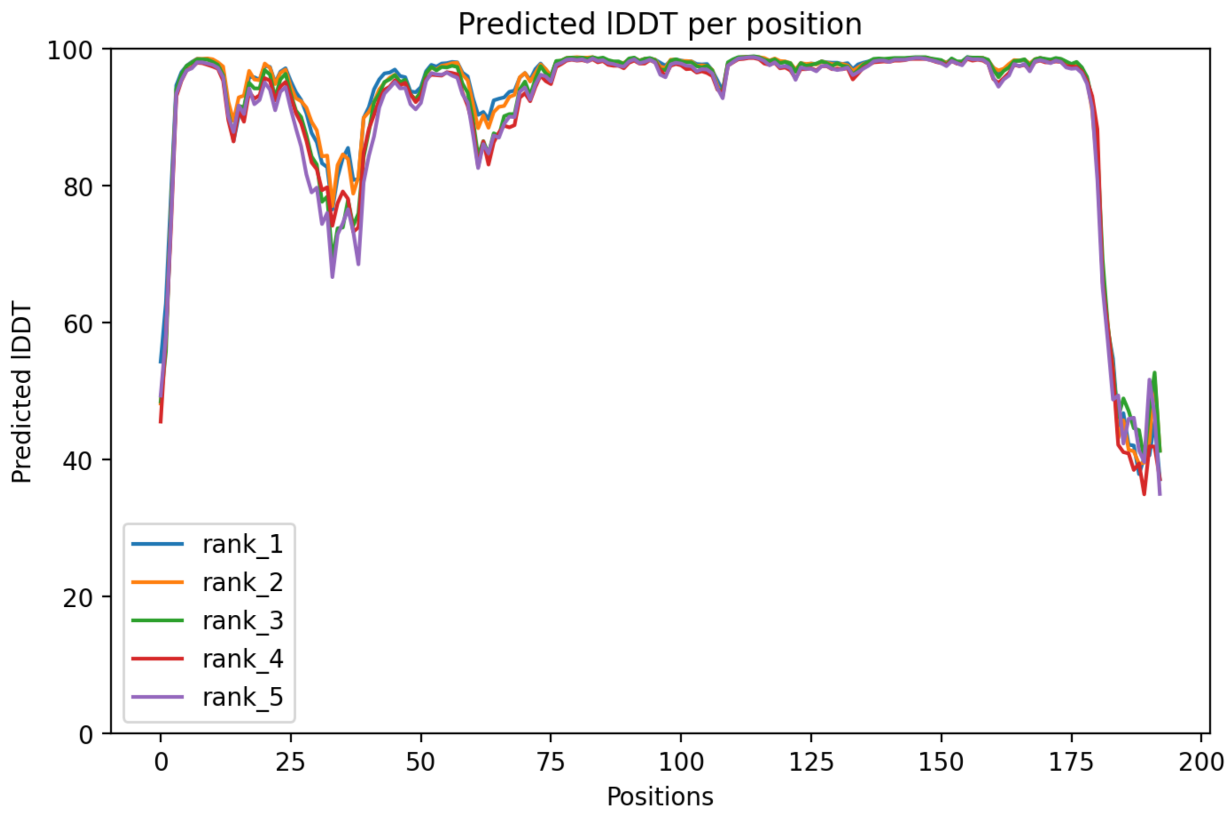

Figure S25: Predicted lDDT per position of top 5 conformations of RHOA G17V generated by ColabFold

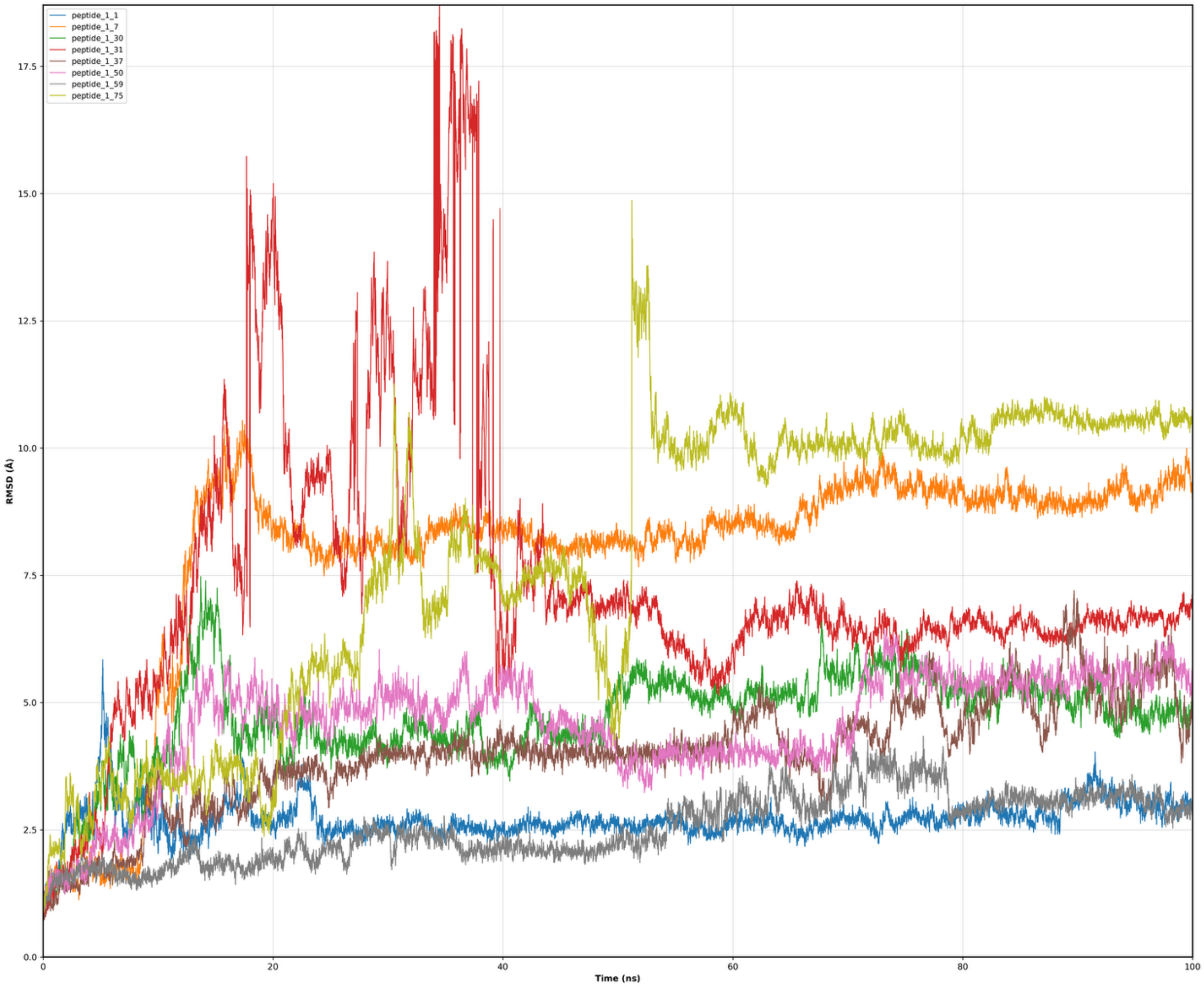

Figure S 26: $RMSD_{complex}$ of all peptide conformations in complex with RHOA G17V during the 100-ns MD simulations

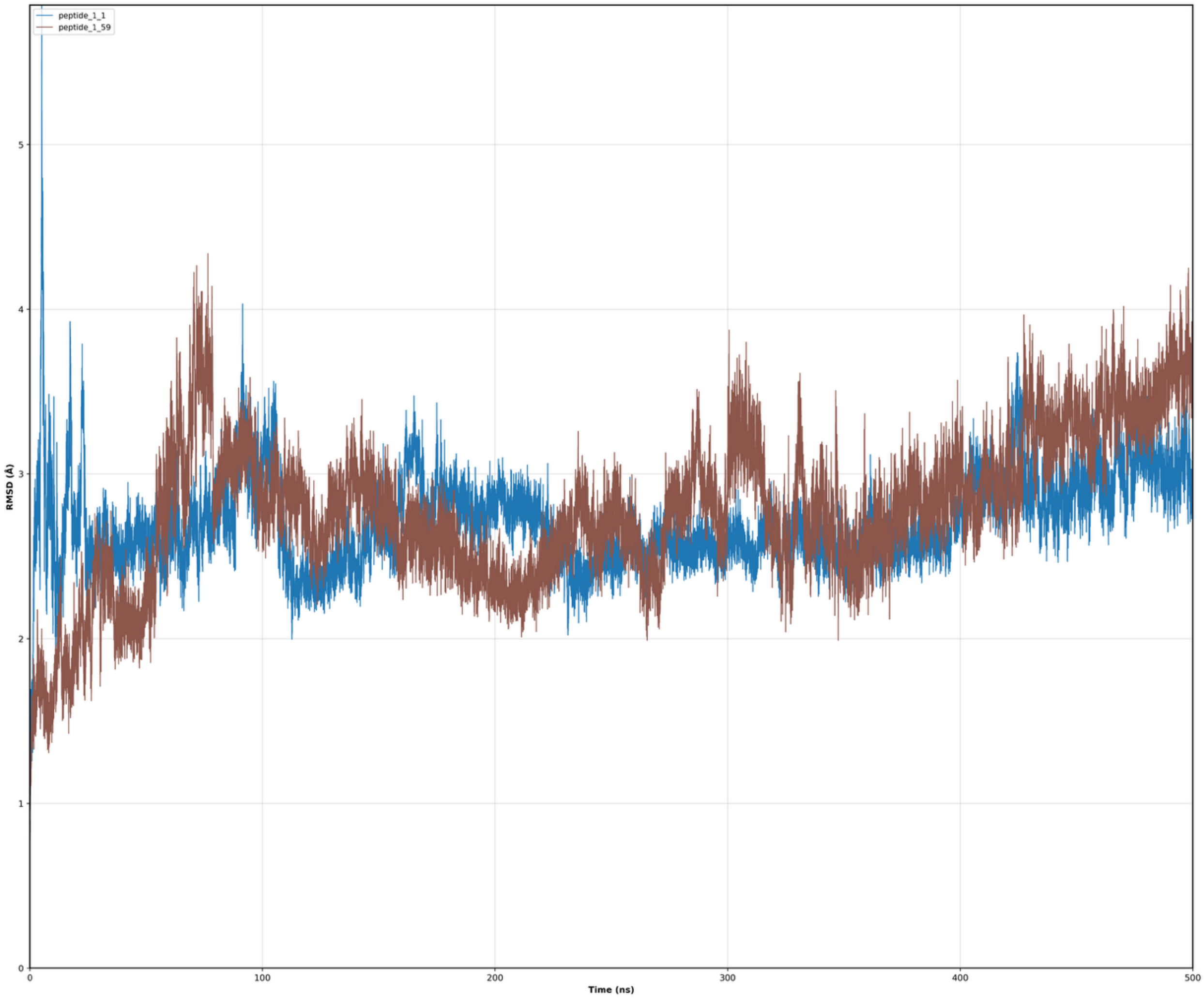

Figure S27: RMSD$_{complex}$ of top two conformations in complex with RHOA G17V during the 500-ns MD simulations

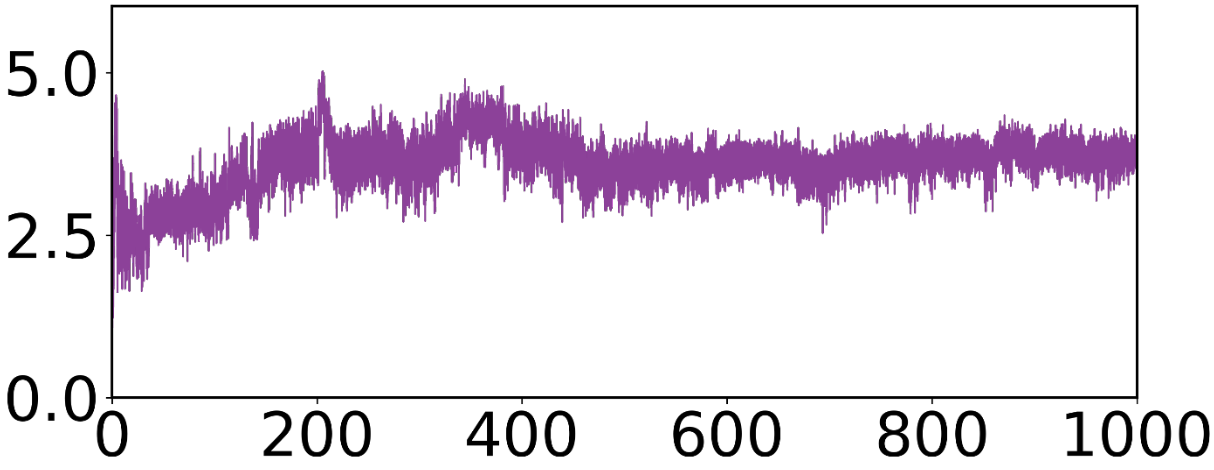

Figure S 28: RMSD$_{peptide}$ of the best peptide conformation in complex with RHOA G17V during the 1000-ns MD simulations, which shows a decent level of backbone rigidity.

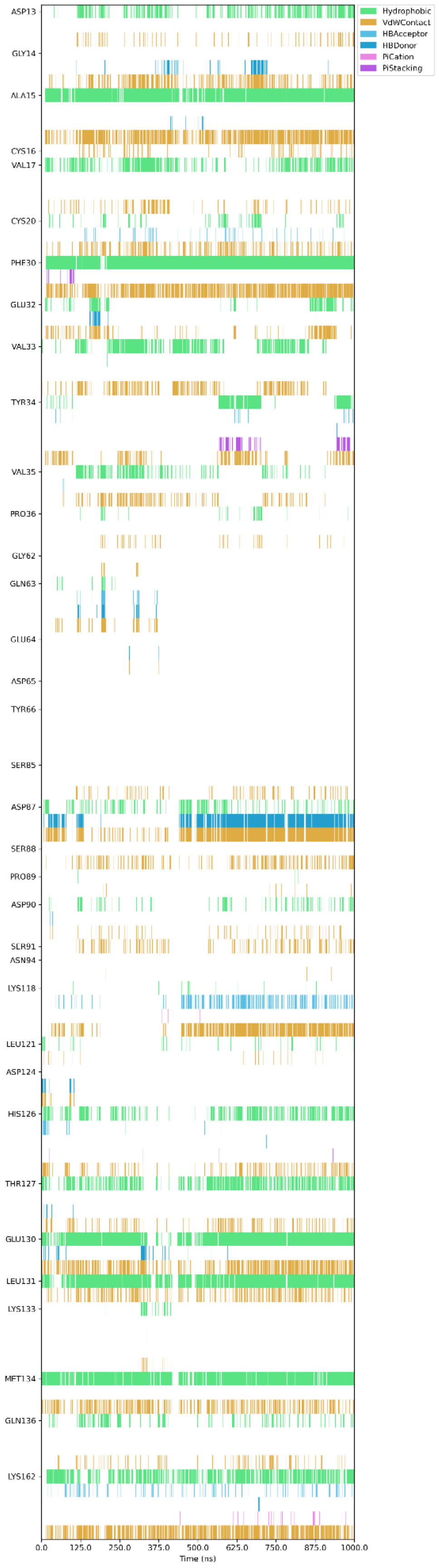
ASP13
GLY14
ALA15
CYS16
VAL17
CYS20
PHE30
GLU32
VAL33
TYR34
VAL35
PRO36
GLY62
GLN63
GLU64
ASP65
TYR66
SER85
ASP87
SER88
PRO89
ASP90
SER91
ASN94
LYS118
LEU121
ASP124
HIS126
THR127
GLU130
LEU131
LYS133
MET134
GLN136
LYS162
Hydrophobic
VdWContact
HBAcceptor
HBDonor
PiCation
PiStacking
0.0
125.0
250.0
375.0
500.0
625.0
750.0
875.0
1000.0
Time (ns)

Figure S 29: 2D fingerprint interaction plot between the best conformation and with RHOA G17V during the 1000-ns MD simulations show high hydrophobic interaction frequency between V17 and the peptide of interest.

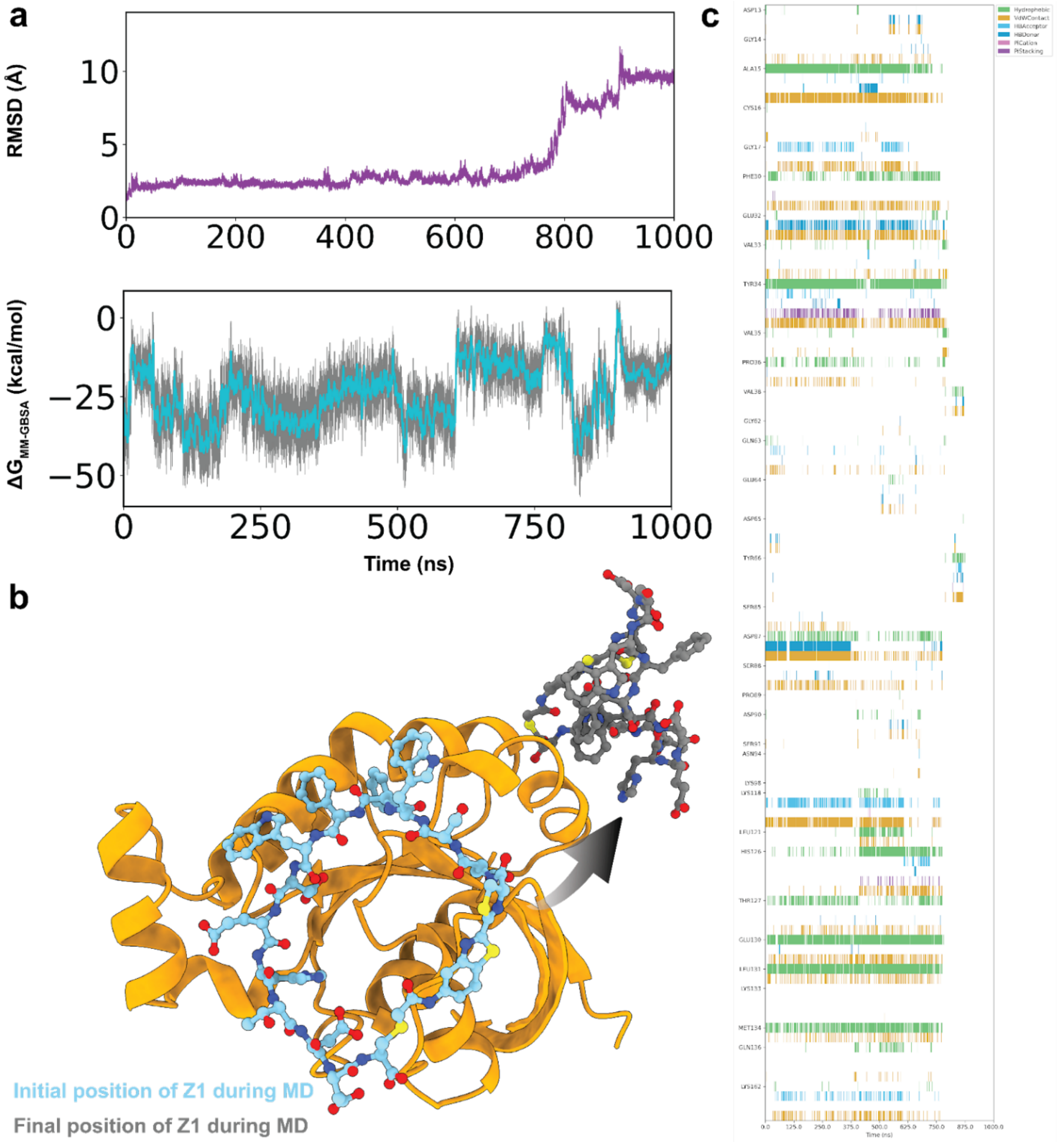

Figure S30: MD results of conformation 1 of Z1 in the binding site of wildtype RhoA. (a) 1000 ns MD result of Z1, including RMSD plot of the whole complex and the average free energy of binding (ΔG_{MM}-G_{BSA}) every 1 ns (sky blue) during 1000 ns MD run against the free energy each frame (gray). (b) Superimposition between the initial state and the final state of the complex showed that Z1 dissociated further away from the binding site during the MD simulation. (c) 2D fingerprint interaction plot between Z1 and with wildtype RhoA during the 1000-ns MD simulations.

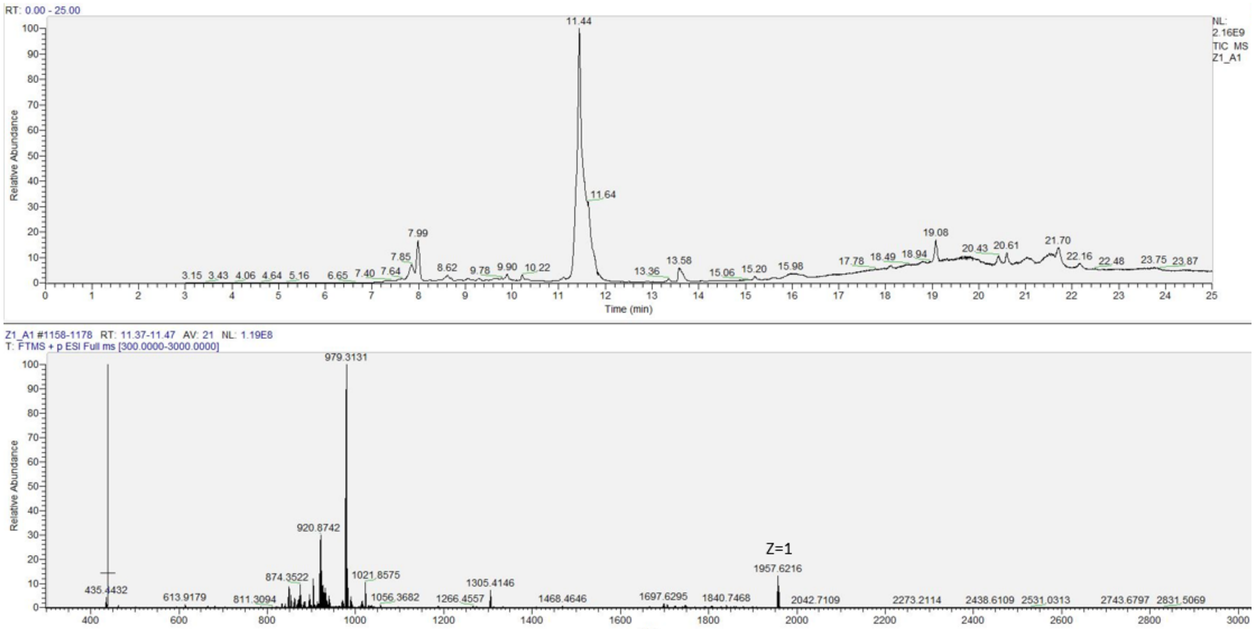

Figure S 31: LC-MS Data for Z1_A1. The TIC chromatogram is shown on top and the extracted masses from the peak are below.

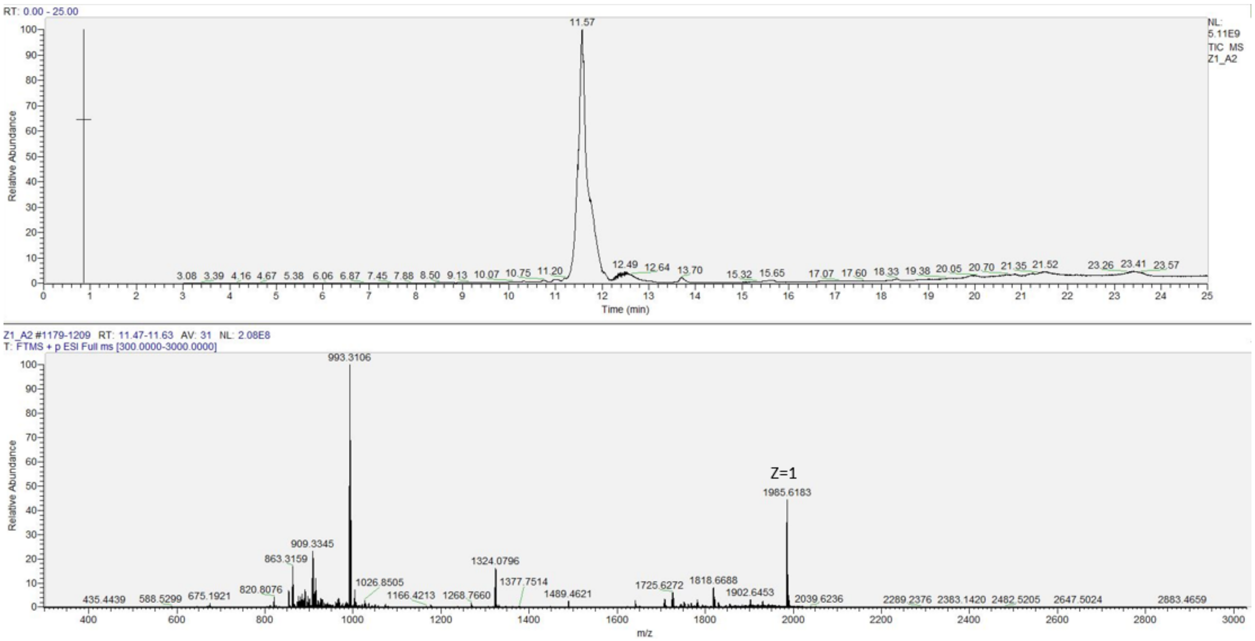

Figure S32: 3LC-MS Data for Z1_A2. The TIC chromatogram is shown on top and the extracted masses from the peak are below.

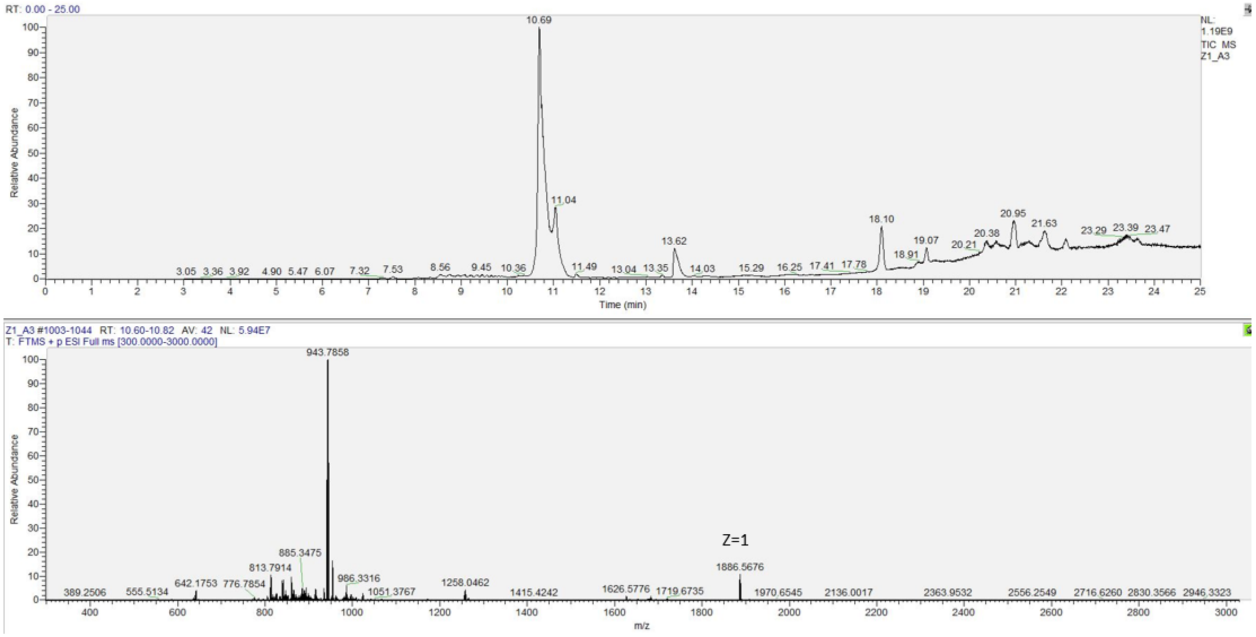

Figure S33: LC-MS Data for Z1_A3. The TIC chromatogram is shown on top and the extracted masses from the peak are below.

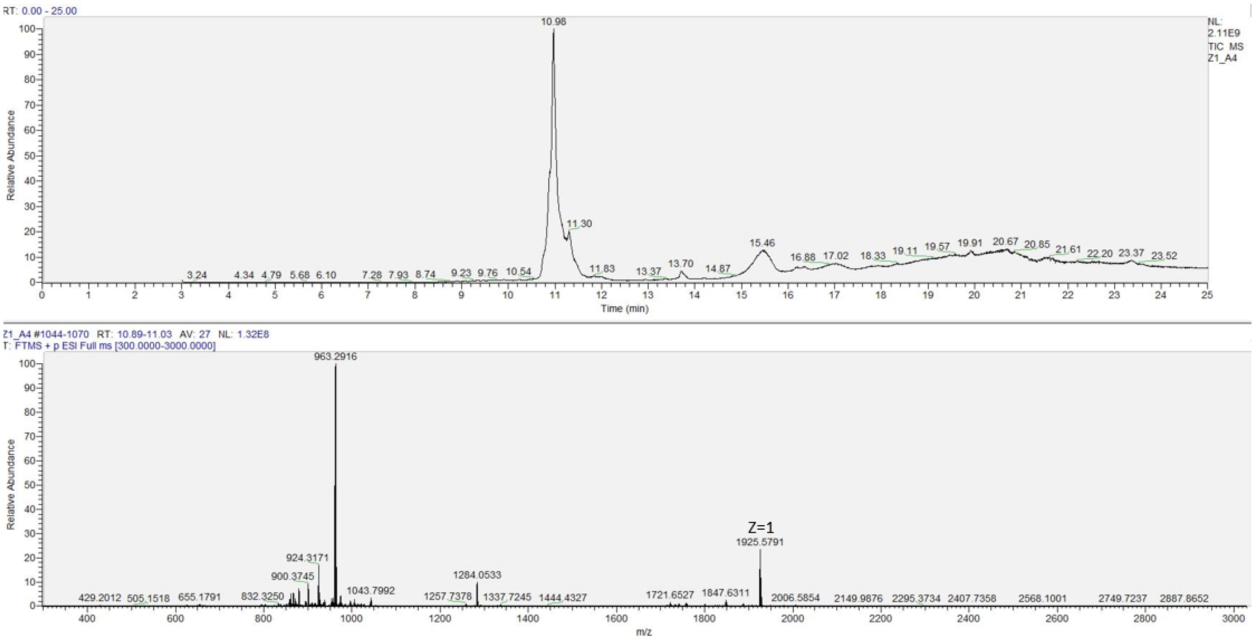

Figure S34: LC-MS Data for Z1_A4. The TIC chromatogram is shown on top and the extracted masses from the peak are below.

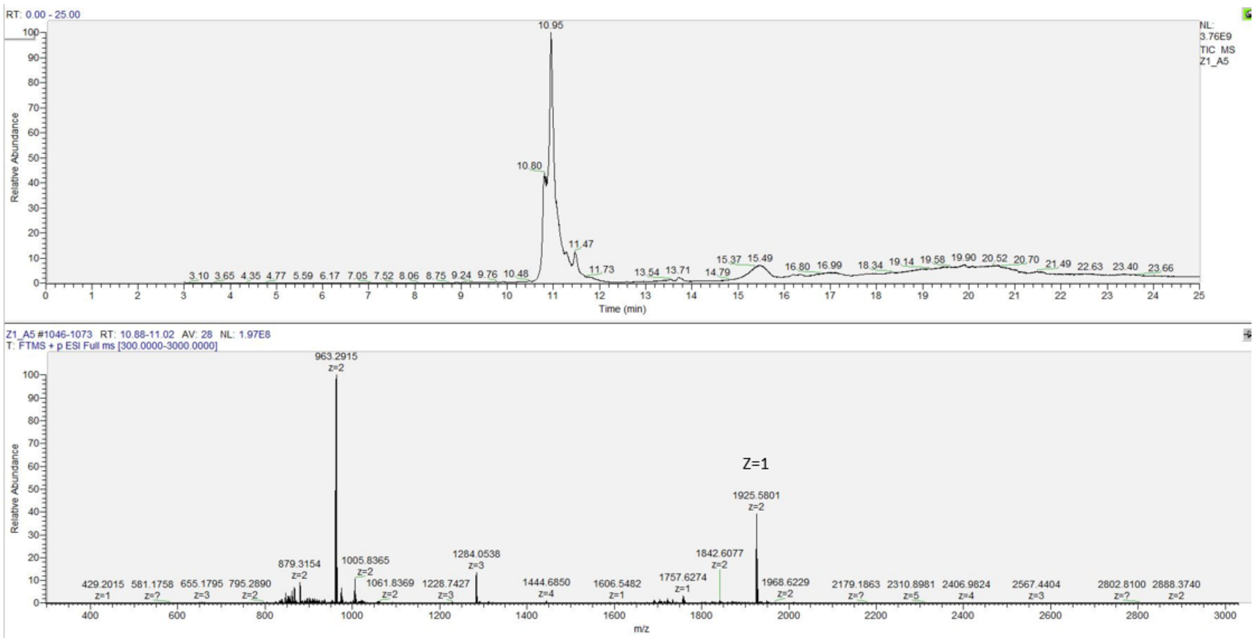

Figure S35: LC-MS Data for Z1_A5. The TIC chromatogram is shown on top and the extracted masses from the peak are below.

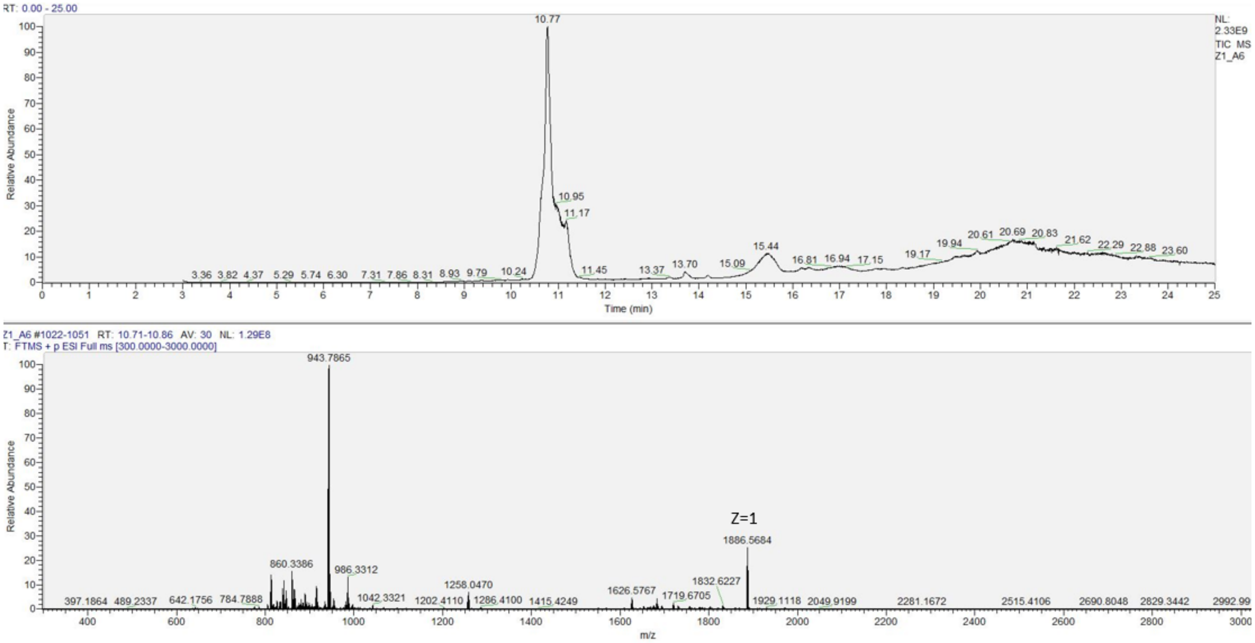

Figure S36: LC-MS Data for Z1_A6. The TIC chromatogram is shown on top and the extracted masses from the peak are below.

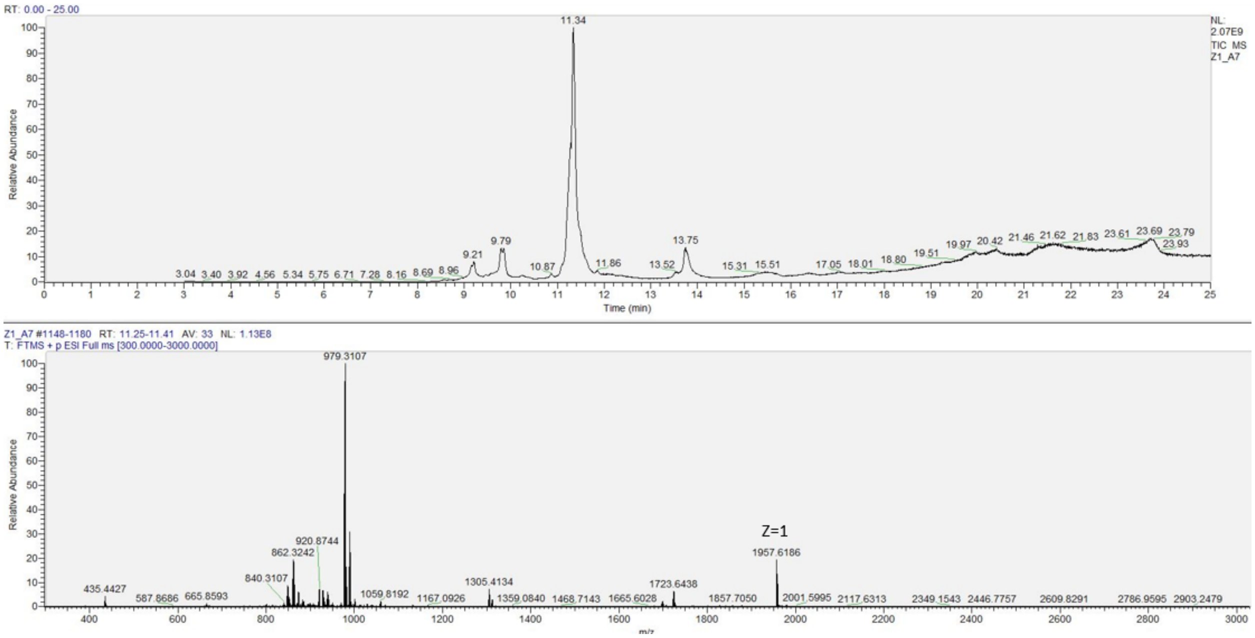

Figure S37: LC-MS Data for Z1_A7. The TIC chromatogram is shown on top and the extracted masses from the peak are below.

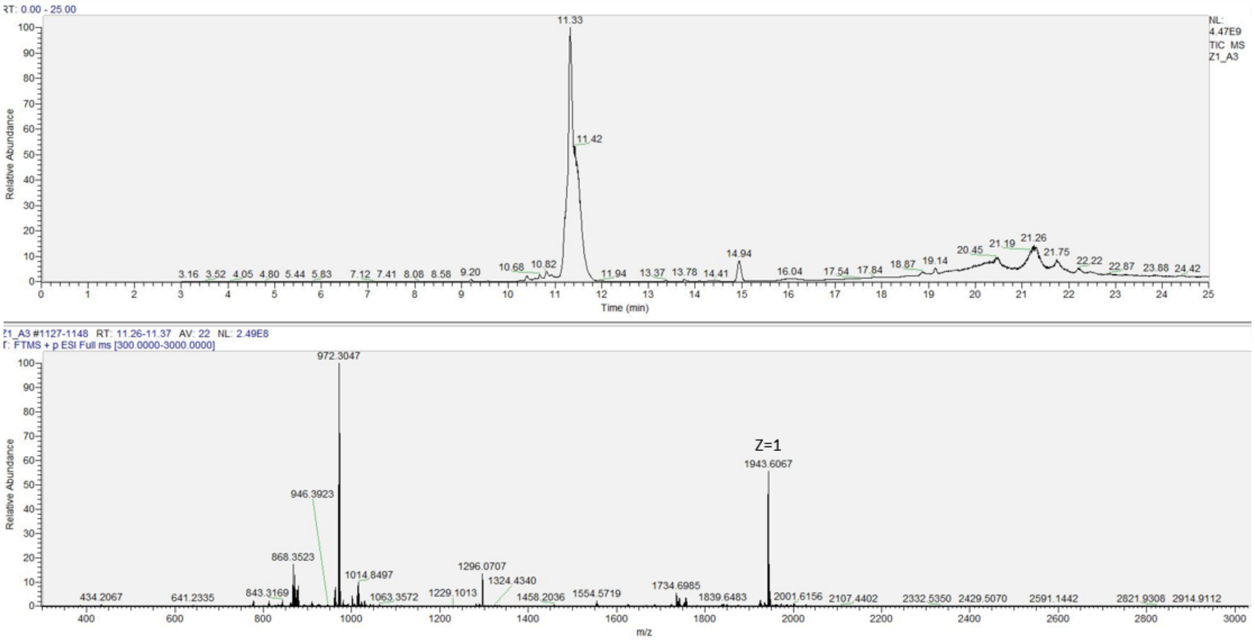

Figure S38: LC-MS Data for Z1_A8. The TIC chromatogram is shown on top and the extracted masses from the peak are below.

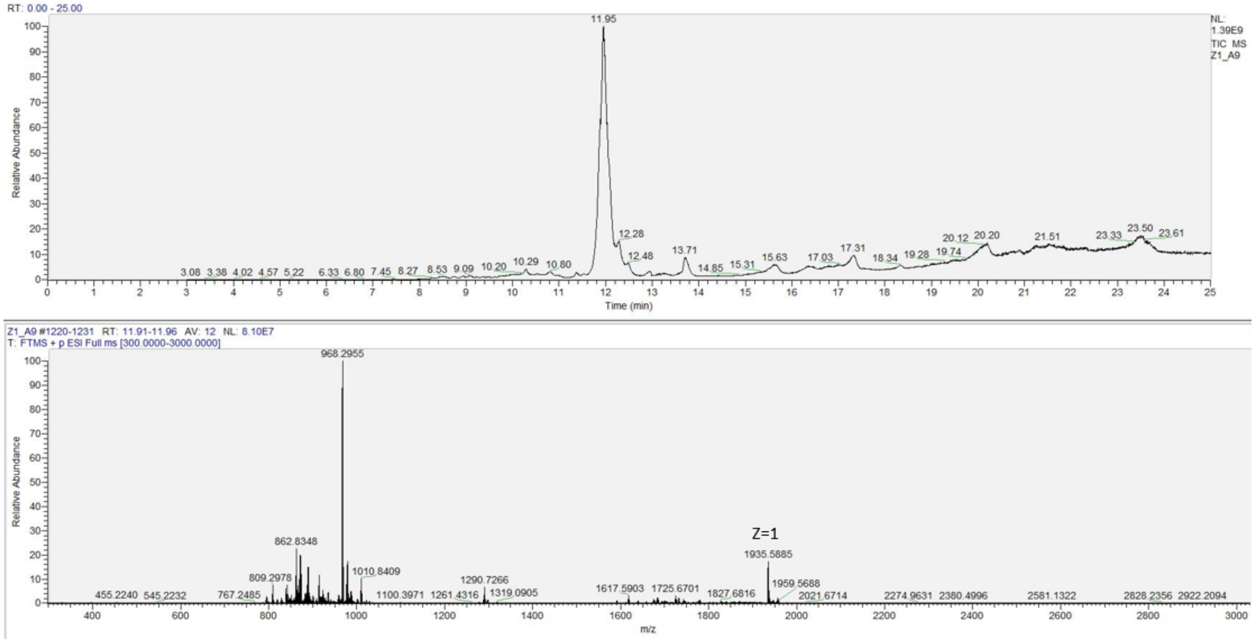

Figure S39: LC-MS Data for Z1_A9. The TIC chromatogram is shown on top and the extracted masses from the peak are below.

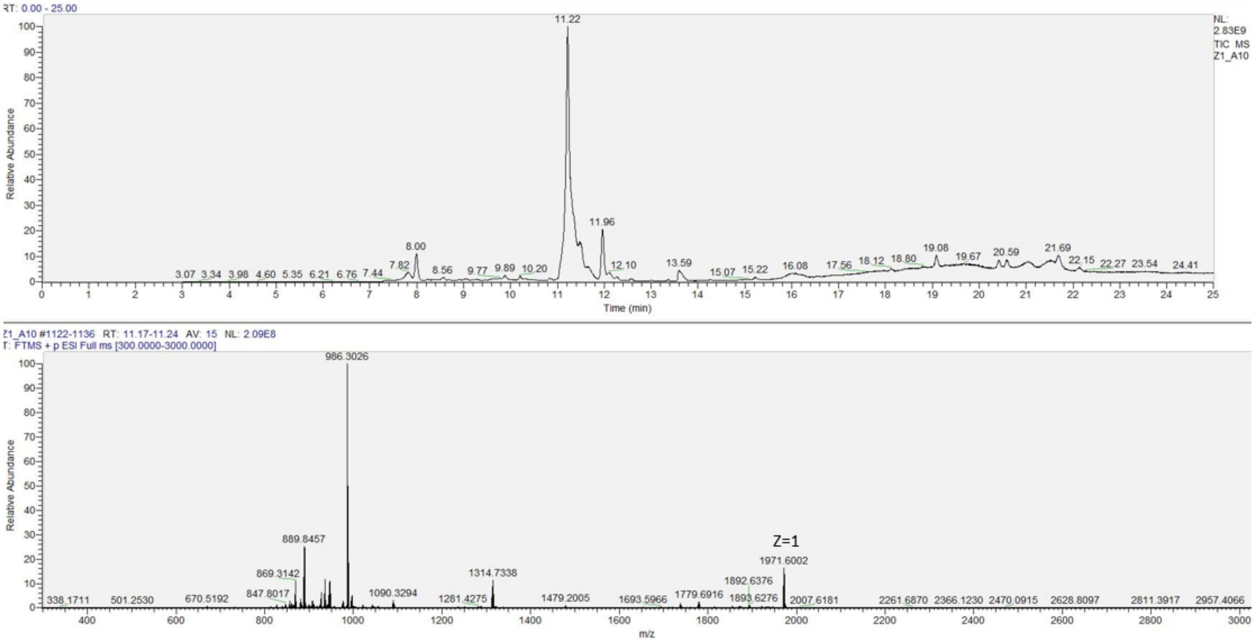

Figure S40: LC-MS Data for Z1_A10. The TIC chromatogram is shown on top and the extracted masses from the peak are below.

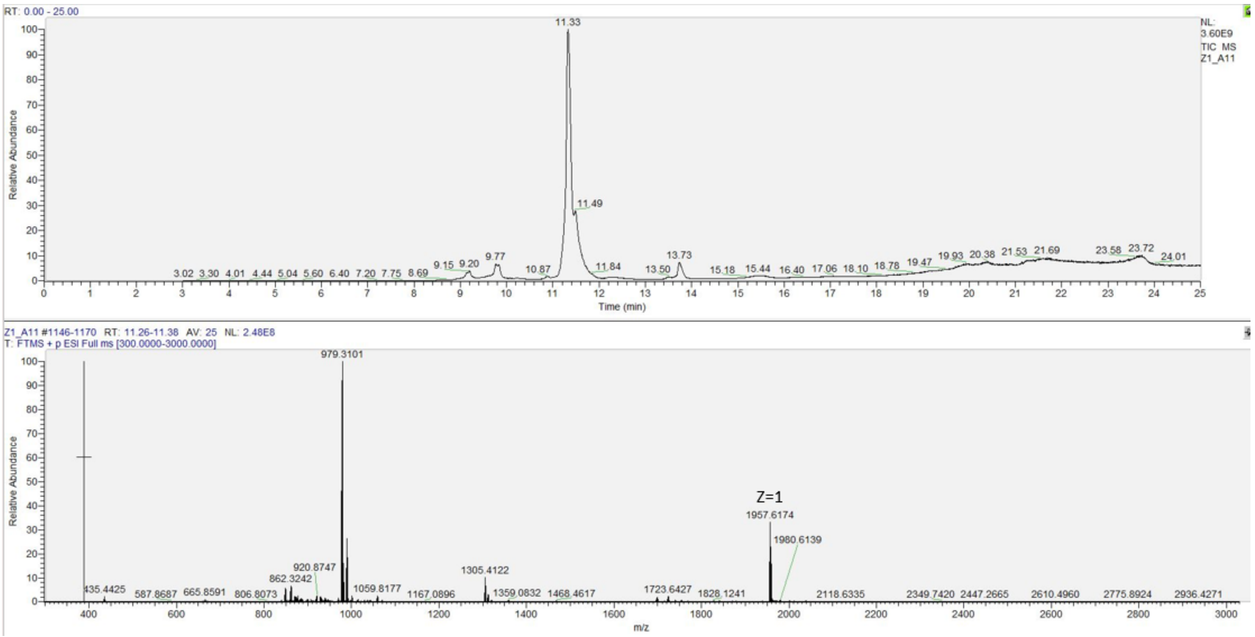

Figure S41: LC-MS Data for Z1_A11. The TIC chromatogram is shown on top and the extracted masses from the peak are below.

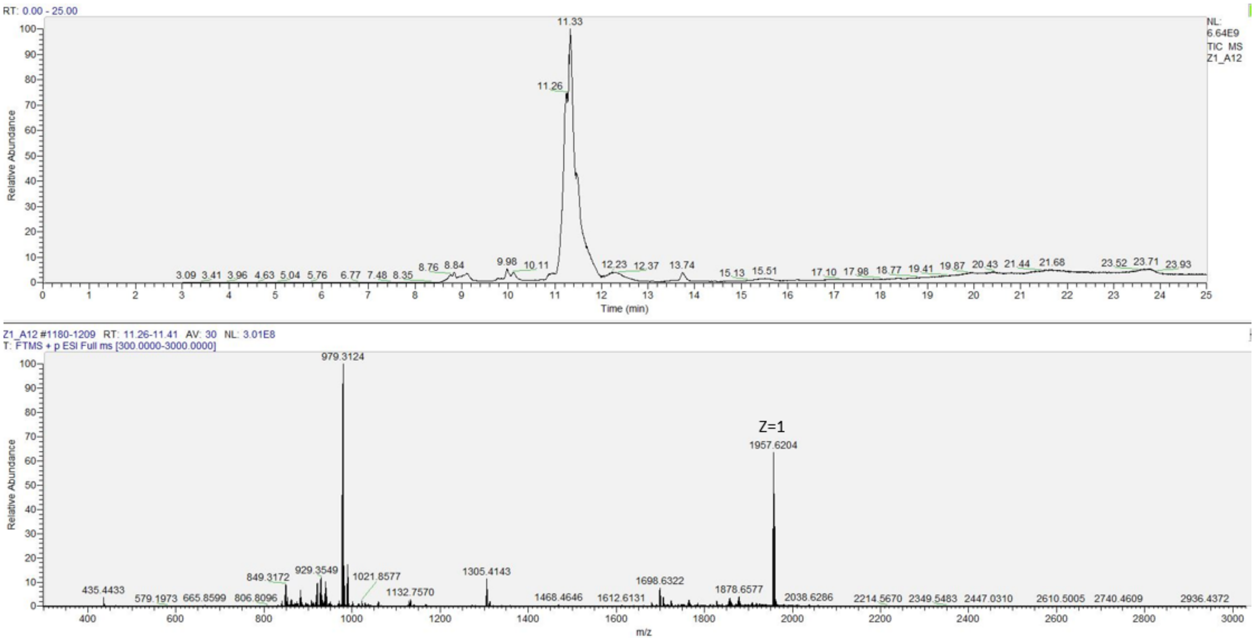

Figure S42: LC-MS Data for Z1_A12. The TIC chromatogram is shown on top and the extracted masses from the peak are below.

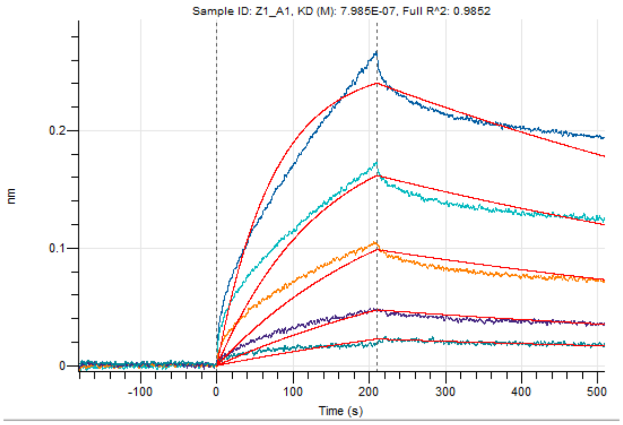

Figure S43: BLI binding kinetics of Z1_A1 against RhoA G17V

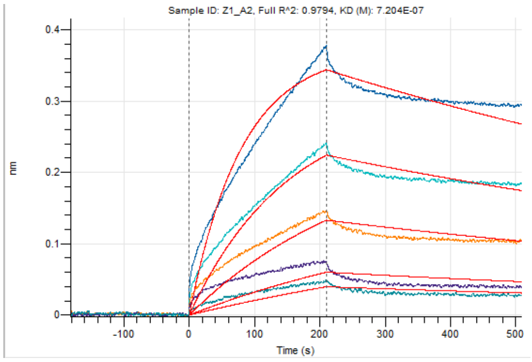

Figure S44: BLI binding kinetics of Z1_A2 against RhoA G17V

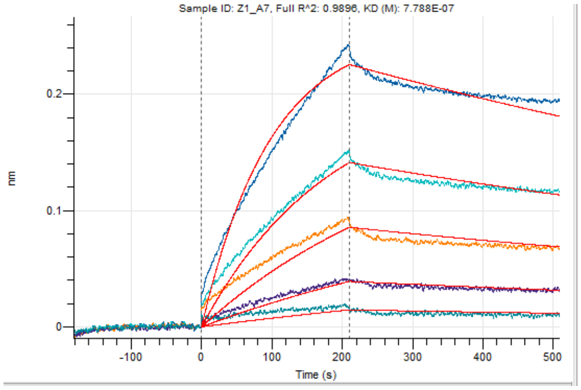

Figure S45: BLI binding kinetics of Z1_A7 against RhoA G17V

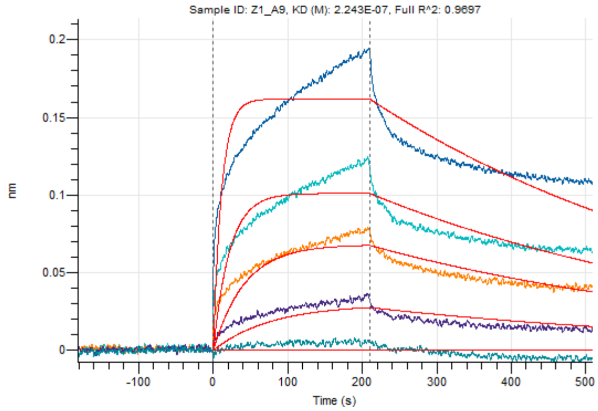

Figure S46: BLI binding kinetics of Z1_A9 against RhoA G17V

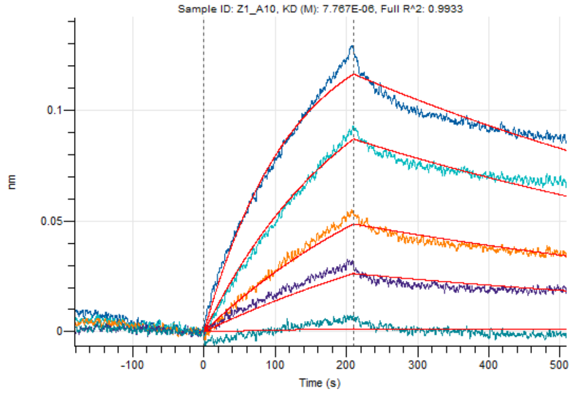

Figure S47: BLI binding kinetics of Z1_A10 against RhoA G17V

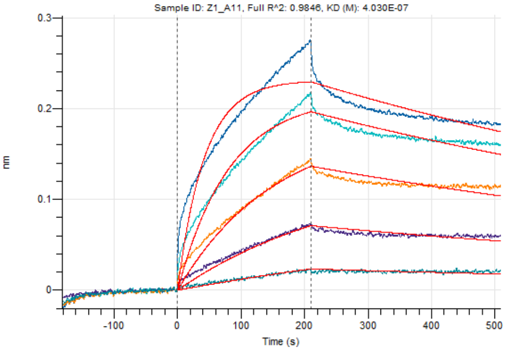

Figure S48: BLI binding kinetics of Z1_A11 against RhoA G17

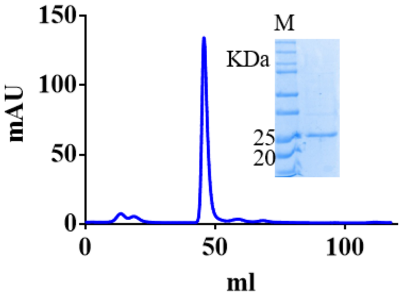

Figure S49: Expression of RhoA wild protein (24.9) KDa

A).

Round 1, 140512 reads

V1	V2	V3	V4	V5	V6	V7	V8	V9	V10	V11	V12	n	percent
C	T	Y	D	A	M	P	G	K	A	V	TAG	1966	1.472935
C	L	H	L	G	G	C	G	V	V	G	TAG	1534	1.149279
C	Q	C	R	D	R	G	G	S	S	P	TAG	1433	1.073609
C	V	W	S	G	N	A	P	R	W	T	TAG	1374	1.029406
C	T	F	R	Q	K	F	G	W	S	S	TAG	1197	0.896797
C	M	G	E	D	A	A	S	L	R	K	TAG	1054	0.789661
C	V	S	A	D	R	TAG	V	L	Y	G	TAG	1003	0.751452
C	C	L	W	S	A	V	R	V	W	P	TAG	968	0.725229
C	V	C	C	I	P	V	A	A	R	V	TAG	947	0.709496
C	S	L	N	G	TAG	S	V	S	K	G	TAG	935	0.700506

Round 4, 149896 reads

	V1	V2	V3	V4	V5	V6	V7	V8	V9	V10	V11	V12	n	percent
RhoA1	C	L	Y	W	N	E	W	P	H	P	M	TAG	59447	40.5151
	C	I	W	C	TAG	W	P	G	S	T	G	TAG	34898	23.78414
	C	H	K	S	S	W	V	W	A	R	H	TAG	27856	18.98479
	C	TAG	W	V	S	C	N	D	L	E	G	TAG	9384	6.395507
	C	F	F	E	E	H	W	H	W	C	G	TAG	1336	0.910528
	C	TAG	W	G	G	A	M	W	D	G	G	TAG	989	0.674036
	C	S	A	C	T	G	H	P	M	G	E	TAG	287	0.1956
	C	L	Y	W	N	TAG	W	P	H	P	M	TAG	230	0.156753
	C	L	Y	C	N	E	W	P	H	P	M	TAG	208	0.141759
	C	I	W	C	TAG	W	P	G	S	T	G	E	178	0.121313

B)

RhoA-NHS 3rd

NGS project: 22468Wns

	V1	V2	V3	V4	V5	V6	V7	V8	V9	V10	V11	V12	n
1	C	F	S	L	F	E	W	D	D	D	G	TAG	191
2	C	W	N	W	L	E	N	S	V	F	G	TAG	177
3	C	D	W	V	A	W	L	P	I	R	W	TAG	122
4	C	W	R	V	F	I	W	G	Q	G	P	TAG	121
5	C	W	N	W	W	S	A	L	T	G	S	TAG	120
6	C	C	S	L	F	W	E	E	D	E	M	TAG	116
7	C	V	W	C	W	F	I	D	N	P	A	TAG	101
8	C	W	S	W	S	E	L	L	G	W	G	TAG	96
9	C	V	W	W	W	P	F	D	H	G	R	TAG	92
10	C	K	F	V	F	K	W	V	R	Y	C	TAG	90
11	C	F	Y	V	W	K	F	Q	S	N	C	TAG	86
12	C	N	W	F	L	G	F	I	D	D	D	TAG	83
13	C	W	F	A	W	R	W	V	G	E	S	TAG	83
14	C	W	W	S	I	V	V	S	G	R	G	TAG	80
15	C	K	K	V	I	W	L	V	C	N	G	TAG	75
16	C	L	K	V	T	W	L	Y	R	H	C	TAG	71
17	C	V	I	G	L	V	V	L	G	K	H	TAG	69

Table S1: Top sequences identified from two different strategies of selection to prevent nonspecific binders A) Hit peptides from selection using Streptavidin beads B) Hit sequences identified using alternate selection using NHS beads

Table 2, Streptavidin >NHS >NHS >GSH beads selection

Peptides	aa	Percentage
1	CDSWFFWDEHTDDC	0.64
2	CQLLDEWWPESDEC	0.17
3	CYSRIHLWVGVVSC	0.13
4	CEFD FLWG EEAC	0.11
5	CLIMYYLVHVDGGC	0.09
6	CTRL EGYPT	0.08
7	CHYR WHLQ VWYC	0.08
8	CYRHYSYFLVYASC	0.08
9	CDRWFDPWDVSPEC	0.07
10	CVLT MELTS VGC	0.07

Table 3, Streptavidin >NHS > GSH >NHS beads selection

Peptides	aa	Percentage
1	CDSWFFWDEHTDDC	1.3
2	CGYLDNWWEVGYSC	1.2
3	CTLLS PWPW ADSEC	0.36
4	CNN DLF NSG DLGC	0.32
5	CMGTDCWWTFEDSC	0.24
6	CELF WF PEEDAC	0.18
7	CMYK LADLES PFMC	0.17
8	CW VPDLD WAHEC	0.16
9	CQLLDEWWPESDEC	0.16
10	CLLDEYWNVSAVAC	0.15

Table 4, Streptavidin >NHS > Streptavidin > NHS beads selection

Peptides	aa	Percentage
1	CDSWFFWDEHTDDC	3.3
2	CGYLDNWWEVGYSC	0.78
3	CTLLS PWPW ADSEC	0.56
4	CDFDFWAETESQTC	0.43
5	CEFDFLWGEEAC	0.32
6	CDL DWW SEM SSC	0.30
7	CELF WF PEEDAC	0.30
8	CLD LFW SGD LGC	0.26
9	CQLLDEWWPESDEC	0.20
10	CET MFWEDREWEC	0.10

Table S2: Top sequences identified from three different strategies of selection to prevent nonspecific binders

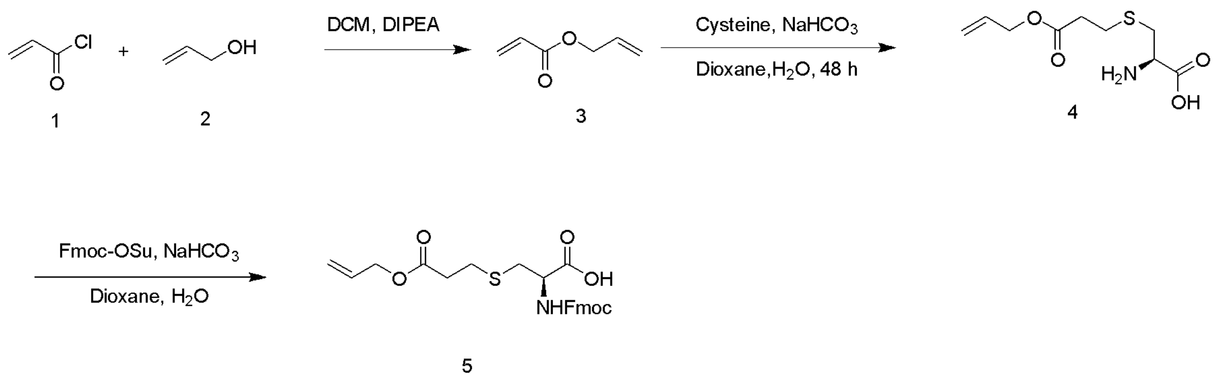

Figure S50: Synthesis of N-(((9H-fluoren-9-yl) methoxy) carbonyl)-S-(3-(allyloxy)-3-oxopropyl)-L-cysteine

Allyl acrylate (3):

A solution of the starting alcohol (1.0 eq) in dry dichloromethane (40 mL) was cooled to 0 °C. DIPEA (3.0 eq) was added, followed by dropwise addition of the corresponding Acryloyl chloride (1.2 eq). The mixture was allowed to warm to room temperature and stirred for 4 h. After completion, the reaction was quenched with aqueous NH_4Cl, extracted with DCM, dried over $MgSO_4$, filtered, and concentrated (Volatile). The crude product was used without further purification.

S-(3-(allyloxy)-3-oxopropyl)-L-cysteine(4):

The cysteine (1.0 eq) was dissolved in water and adjusted to pH 7.5 using $NaHCO_3$. The compound 3 (1.1 eq), dissolved in 1,4-dioxane, was added dropwise at 0 °C. The reaction was warmed to room temperature and stirred for 48 h. The mixture was lyophilized and the crude coupled product was used for next step.

N-(((9H-fluoren-9-yl)methoxy)carbonyl)-S-(3-(allyloxy)-3-oxopropyl)-L-cysteine (5):

Compound 4 (1.0 eq) was dissolved in 10 mL of 1:1 dioxane/H2O, and Fmoc-Osu (1.1 eq) was added and stirred at room temperature for 6 h. Aqueous HCl (1 M, 10 mL) was added to the mixture, and the organic layer was extracted with EtoAc (3 × 5 mL). The combined organic layers were washed with 1 M HCl, water, and brine. Then, the combined aqueous layers were extracted with EtOAc, dried, and evaporated under reduced pressure. The crude product was then purified using flash column chromatography affording compound 5 as a white solid. ^1H NMR (400 MHz, $CDCl_3$) δ 7.78 (d, J = 7.5 Hz, 2H), 7.63 (d, J = 7.6 Hz, 2H), 7.42 (t, J = 7.5 Hz, 2H), 7.33 (t, J = 7.4 Hz, 2H), 5.92 (td, J = 10.7, 5.2 Hz, 1H), 5.82 (d, J = 8.0 Hz, 1H), 5.33 (d, J = 17.2 Hz, 1H), 5.25 (d, J = 10.4 Hz, 1H), 4.69 (s, 1H), 4.62 (d, J = 5.8 Hz, 2H), 4.44 (d, J = 7.2 Hz, 2H), 4.26 (t, J = 6.9 Hz, 1H), 3.10 (d, J = 5.1 Hz, 2H), 2.88 (t, J = 7.1 Hz, 2H), 2.66 (t, J = 7.2 Hz, 2H).

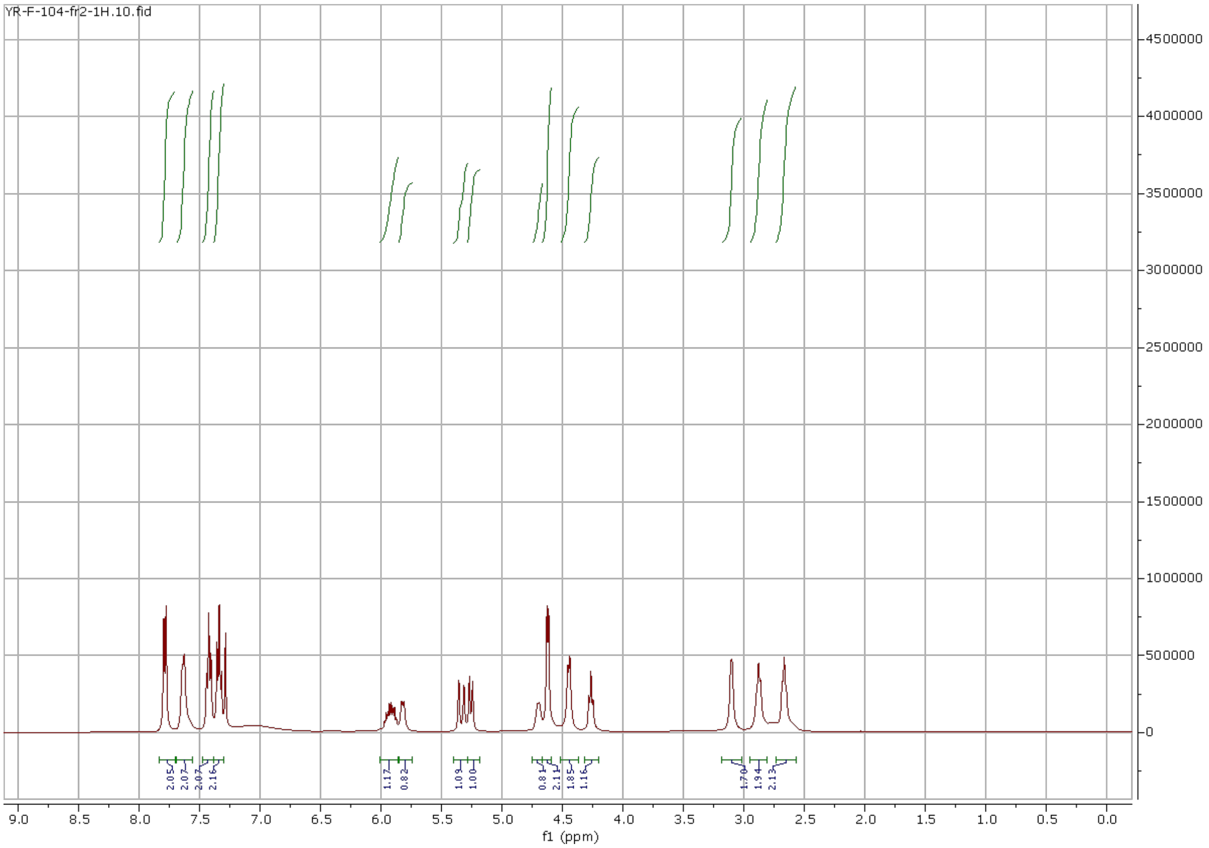

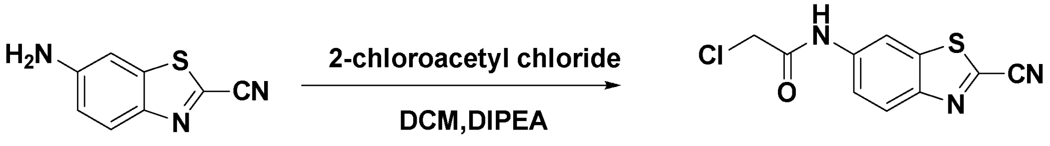

2-Chloro-N-(2-cyanobenzo[d]thiazol-6-yl)acetamide (CAmCBT):

Diisopropylethylamine (0.252 mL, 1.37 mmol) and 2-chloroacetyl chloride (0.06 mL, 0.685 mmol) was added to a solution of 4 (0.120 g, 0.685 mmol) in dry DCM (5 mL) at 4 °C. The reaction was then left to stir at room temperature for 16 h and the solvent removed in vacuo. The residue was then purified by silica gel column chromatography (10-70% ethyl acetate in hexane, 20 min) to afford CAmCBT (0.115 g, 59%) as a yellow solid. ^{1}H NMR (400 MHz, DMSO) δ 10.81 (s, 1H), 8.74 (d, *J* = 2.1 Hz, 1H), 8.23 (d, *J* = 9.0 Hz, 1H), 7.76 (dd, *J* = 9.0, 2.1 Hz, 1H), 4.35 (s, 2H).

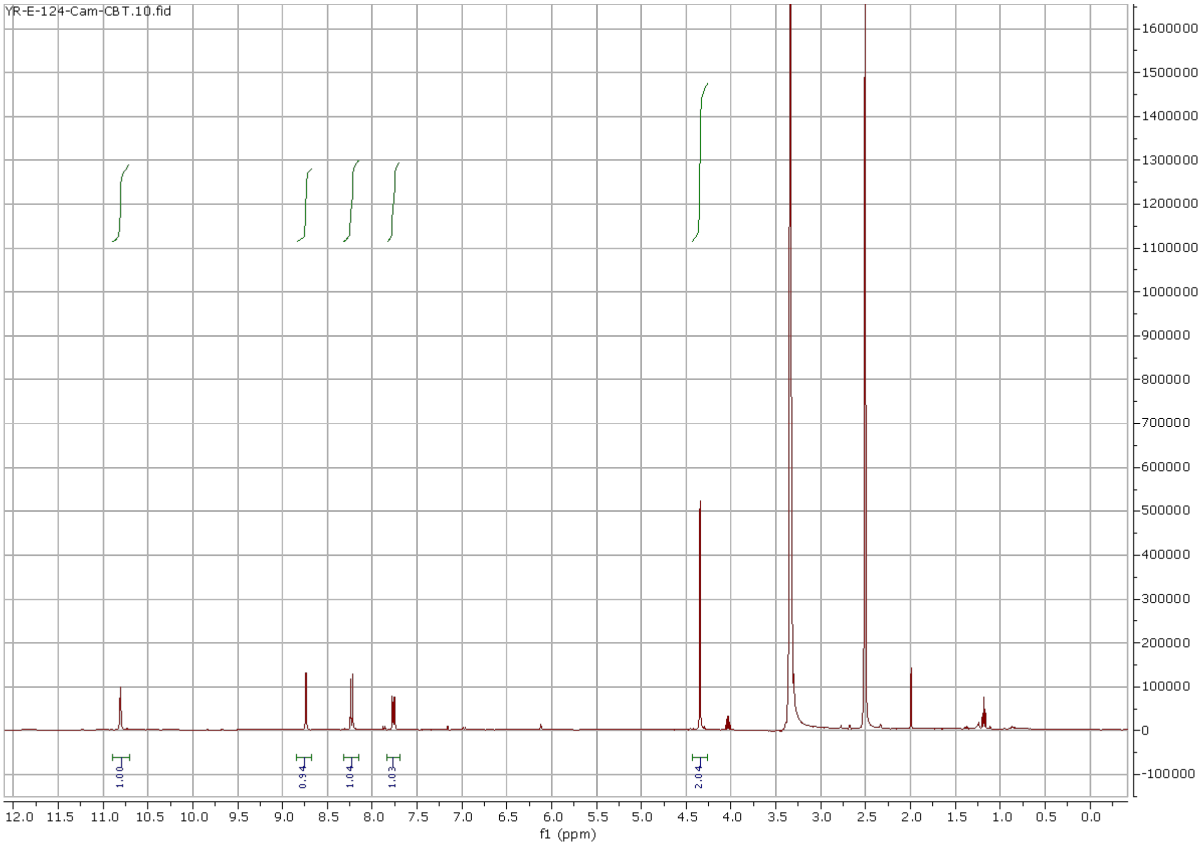

Supplementary R Scripts

1. Amino Acid Analysis (translation of DNA into amino acids and abundance of peptide sequences)

```
library(microseq)
library(RColorBrewer)
library(dplyr)
library(stringr)
library(gplots)
NNK7Ffilt <- readFastq("/22468Wns_RhoA-4th-_S5_L001_R1_001.fastq")
 #path to forward.fastq files
NNK7Rfilt <- readFastq("/22468Wns_N22179/22468Wns_RhoA-4th-
 _S5_L001_R2_001.fastq") #path to reverse .fastq file
#Define the following variables #CX10TAG
libraryseq <- "GCCCAG.{48}GCGGCG.{6}" #change this regex to match
 specific library "GCCCAG.{48}GCGGCG.{6}" for CX10TAG or
 "GCCCAG.{54}GCGGCG.{6}" for CX12C
beginning <- 19 #beginning of library in DNA string
```

```
lib <- 12 #number of codons in the library region (12 for CX10TAG or
 14 for CX12C)
end <- beginning+lib*3
initialcodon <- beginning%/%3*4-1
endcodon <- initialcodon + lib*4
del <- beginning%/%3 #number of codons before library
aa <-
 c("A","C","D","E","F","G","H","I","K","L","M","N","P","Q","R","S","T",
 "V","W","Y","TAG")

#slices out matches that contain start followed by 24 bases to reverse
 primer
NNK7Ffilt21 <- gregexpr(libraryseq,NNK7Ffilt[[2]],extract = TRUE)
NNK7Rrevcomp <- reverseComplement(NNK7Rfilt[[2]],reverse = TRUE)
 #gives reverse complement of reverse reads
NNK7Rcompfilt21 <- gregexpr(libraryseq,NNK7Rrevcomp,extract = TRUE)

#this compares the forward and reverse strands, only allowing for one
 mismatch in the primers, no mismatches allowed in the library region
n <- length(NNK7Ffilt21)
NNK7Fgood <- vector()
for(i in c(1:n)){
  if(NNK7Ffilt21[[i]][1] == NNK7Rcompfilt21[[i]][1]){
    NNK7Fgood[i] <- NNK7Ffilt21[[i]]
  }
  else{
    split <- strsplit(c(NNK7Ffilt21[[i]],NNK7Rcompfilt21[[i]]), split
 = "")
    diff <- which(split[[1]] != split[[2]])
    if(length(diff) < 2 && length(diff) > 0){
      for(x in c(1:length(diff))){
        if(diff[[x]] < beginning || diff[[x]] > end){
          NNK7Fgood[i] <- NNK7Ffilt21[[i]]
        }
        else{
          NNK7Fgood[i] <- ""
        }
      }
    }
    else{
      NNK7Fgood[i] <- ""
    }
  }
}
```

```
NNK7Fgood <- as.data.frame(NNK7Fgood)
NNK7Fgood <- NNK7Fgood[!apply(is.na(NNK7Fgood) | NNK7Fgood == "", 1,
 all),]

#this separates nucleotides into codons
codons <- gsub("(...)", "\\1 \\2", NNK7Fgood)

#this creates dataframe of sequences with reads organized by frequency
seqcount <- as.data.frame(sort(table(codons), decreasing = TRUE))

#this generates a matrix that contains amino acids in library region
l <- length(codons)
AAs <- matrix(0,l,lib)
AA <- gregexpr("\\s(TT[TC])",codons,useBytes = FALSE)
  l <- length(AA)
  for(a in c(1:l)){
    l2 <- length(AA[[a]])
    for(b in c(1:l2)){
      value <- AA[[a]][b]
      if(value > initialcodon && value < endcodon){
        AAs[a,(value%/%4 - (del-1))] <- "F"
      }
    }
  }
AA <- gregexpr("(\\sTT[AG])|(\\sCT[GACT])",codons,useBytes = FALSE)
  l <- length(AA)
  for(a in c(1:l)){
    l2 <- length(AA[[a]])
    for(b in c(1:l2)){
      value <- AA[[a]][b]
      if(value > initialcodon && value < endcodon){
        AAs[a,(value%/%4 - (del-1))] <- "L"
      }
    }
  }
AA <- gregexpr("(\\sTC[GCAT])|(\\sAG[TC])",codons,useBytes = FALSE)
  l <- length(AA)
  for(a in c(1:l)){
    l2 <- length(AA[[a]])
    for(b in c(1:l2)){
      value <- AA[[a]][b]
      if(value > initialcodon && value < endcodon){
        AAs[a,(value%/%4 - (del-1))] <- "S"
      }
```

```
      }
    }
  AA <- gregexpr("\\sTA[TC]",codons,useBytes = FALSE)
    l <- length(AA)
    for(a in c(1:l)){
      l2 <- length(AA[[a]])
      for(b in c(1:l2)){
        value <- AA[[a]][b]
        if(value > initialcodon && value < endcodon){
          AAs[a,(value%/%4 - (del-1))] <- "Y"
        }
      }
    }
  AA <- gregexpr("\\sTAG",codons,useBytes = FALSE)
    l <- length(AA)
    for(a in c(1:l)){
      l2 <- length(AA[[a]])
      for(b in c(1:l2)){
        value <- AA[[a]][b]
        if(value > initialcodon && value < endcodon){
          AAs[a,(value%/%4 - (del-1))] <- "TAG"
        }
      }
    }
  AA <- gregexpr("\\sTAA",codons,useBytes = FALSE)
    l <- length(AA)
    for(a in c(1:l)){
      l2 <- length(AA[[a]])
      for(b in c(1:l2)){
        value <- AA[[a]][b]
        if(value > initialcodon && value < endcodon){
          AAs[a,(value%/%4 - (del-1))] <- NA
        }
      }
    }
  AA <- gregexpr("\\sTG[TC]",codons,useBytes = FALSE)
    l <- length(AA)
    for(a in c(1:l)){
      l2 <- length(AA[[a]])
      for(b in c(1:l2)){
        value <- AA[[a]][b]
        if(value > initialcodon && value < endcodon){
          AAs[a,(value%/%4 - (del-1))] <- "C"
        }
```

```
    }
  }
AA <- gregexpr("\\sTGA",codons,useBytes = FALSE)
  l <- length(AA)
  for(a in c(1:l)){
    l2 <- length(AA[[a]])
    for(b in c(1:l2)){
      value <- AA[[a]][b]
      if(value > initialcodon && value < endcodon){
        AAs[a,(value%/%4 - (del-1))] <- NA
      }
    }
  }
AA <- gregexpr("\\sTGG",codons,useBytes = FALSE)
  l <- length(AA)
  for(a in c(1:l)){
    l2 <- length(AA[[a]])
    for(b in c(1:l2)){
      value <- AA[[a]][b]
      if(value > initialcodon && value < endcodon){
        AAs[a,(value%/%4 - (del-1))] <- "W"
      }
    }
  }
AA <- gregexpr("\\sCC[GCAT]",codons,useBytes = FALSE)
  l <- length(AA)
  for(a in c(1:l)){
    l2 <- length(AA[[a]])
    for(b in c(1:l2)){
      value <- AA[[a]][b]
      if(value > initialcodon && value < endcodon){
        AAs[a,(value%/%4 - (del-1))] <- "P"
      }
    }
  }
AA <- gregexpr("\\sCA[CT]",codons,useBytes = FALSE)
  l <- length(AA)
  for(a in c(1:l)){
    l2 <- length(AA[[a]])
    for(b in c(1:l2)){
      value <- AA[[a]][b]
      if(value > initialcodon && value < endcodon){
        AAs[a,(value%/%4 - (del-1))] <- "H"
      }
```

```
    }
  }
AA <- gregexpr("\\sCA[AG]",codons,useBytes = FALSE)
  l <- length(AA)
  for(a in c(1:l)){
    l2 <- length(AA[[a]])
    for(b in c(1:l2)){
      value <- AA[[a]][b]
      if(value > initialcodon && value < endcodon){
        AAs[a,(value%/%4 - (del-1))] <- "Q"
      }
    }
  }
AA <- gregexpr("(\\sCG[GCAT])|(\\sAG[GA])",codons,useBytes = FALSE)
  l <- length(AA)
  for(a in c(1:l)){
    l2 <- length(AA[[a]])
    for(b in c(1:l2)){
      value <- AA[[a]][b]
      if(value > initialcodon && value < endcodon){
        AAs[a,(value%/%4 - (del-1))] <- "R"
      }
    }
  }
AA <- gregexpr("\\sAT[CAT]",codons,useBytes = FALSE)
  l <- length(AA)
  for(a in c(1:l)){
    l2 <- length(AA[[a]])
    for(b in c(1:l2)){
      value <- AA[[a]][b]
      if(value > initialcodon && value < endcodon){
        AAs[a,(value%/%4 - (del-1))] <- "I"
      }
    }
  }
AA <- gregexpr("\\sATG",codons,useBytes = FALSE)
  l <- length(AA)
  for(a in c(1:l)){
    l2 <- length(AA[[a]])
    for(b in c(1:l2)){
      value <- AA[[a]][b]
      if(value > initialcodon && value < endcodon){
        AAs[a,(value%/%4 - (del-1))] <- "M"
      }
```

```
    }
  }
AA <- gregexpr("\\sAC[GCAT]",codons,useBytes = FALSE)
  l <- length(AA)
  for(a in c(1:l)){
    l2 <- length(AA[[a]])
    for(b in c(1:l2)){
      value <- AA[[a]][b]
      if(value > initialcodon && value < endcodon){
        AAs[a,(value%/%4 - (del-1))] <- "T"
      }
    }
  }
AA <- gregexpr("\\sAA[CT]",codons,useBytes = FALSE)
  l <- length(AA)
  for(a in c(1:l)){
    l2 <- length(AA[[a]])
    for(b in c(1:l2)){
      value <- AA[[a]][b]
      if(value > initialcodon && value < endcodon){
        AAs[a,(value%/%4 - (del-1))] <- "N"
      }
    }
  }
AA <- gregexpr("\\sAA[AG]",codons,useBytes = FALSE)
  l <- length(AA)
  for(a in c(1:l)){
    l2 <- length(AA[[a]])
    for(b in c(1:l2)){
      value <- AA[[a]][b]
      if(value > initialcodon && value < endcodon){
        AAs[a,(value%/%4 - (del-1))] <- "K"
      }
    }
  }
AA <- gregexpr("\\sGT[GACT]",codons,useBytes = FALSE)
  l <- length(AA)
  for(a in c(1:l)){
    l2 <- length(AA[[a]])
    for(b in c(1:l2)){
      value <- AA[[a]][b]
      if(value > initialcodon && value < endcodon){
        AAs[a,(value%/%4 - (del-1))] <- "V"
      }
```

```
    }
  }
AA <- gregexpr("\\sGC[GACT]",codons,useBytes = FALSE)
  l <- length(AA)
  for(a in c(1:l)){
    l2 <- length(AA[[a]])
    for(b in c(1:l2)){
      value <- AA[[a]][b]
      if(value > initialcodon && value < endcodon){
        AAs[a,(value%/%4 - (del-1))] <- "A"
      }
    }
  }
AA <- gregexpr("\\sGA[TC]",codons,useBytes = FALSE)
  l <- length(AA)
  for(a in c(1:l)){
    l2 <- length(AA[[a]])
    for(b in c(1:l2)){
      value <- AA[[a]][b]
      if(value > initialcodon && value < endcodon){
        AAs[a,(value%/%4 - (del-1))] <- "D"
      }
    }
  }
AA <- gregexpr("\\sGA[AG]",codons,useBytes = FALSE)
  l <- length(AA)
  for(a in c(1:l)){
    l2 <- length(AA[[a]])
    for(b in c(1:l2)){
      value <- AA[[a]][b]
      if(value > initialcodon && value < endcodon){
        AAs[a,(value%/%4 - (del-1))] <- "E"
      }
    }
  }
AA <- gregexpr("\\sGG[GACT]",codons,useBytes = FALSE)
  l <- length(AA)
  for(a in c(1:l)){
    l2 <- length(AA[[a]])
    for(b in c(1:l2)){
      value <- AA[[a]][b]
      if(value > initialcodon && value < endcodon){
        AAs[a,(value%/%4 - (del-1))] <- "G"
      }
```

```
    }
  }
AAs <- as.data.frame(AAs)
#this gives unique amino acid sequences
UniqueAAs <- AAs %>% group_by_all() %>% count()
UniqueAAs <- UniqueAAs[order(-UniqueAAs$n),]
UniqueAAs <- UniqueAAs[apply(UniqueAAs,1,function(row) all(row !=
 0)),]
UniqueAAs <- na.omit(UniqueAAs)
UniqueAAsR4S2 <- UniqueAAs #change name to the desired sample

#writes csv files for uniqueAAs and bias heatmaps change path to make
 file
path <- "/RhoA"
write.csv(UniqueAAsR1S1, paste(path,"/UniqueAAsR1S1.csv", sep = ""),
 row.names = F)
write.csv(UniqueAAsR1S2, paste(path,"/UniqueAAsR1S2.csv", sep = ""),
 row.names = F)
write.csv(UniqueAAsR4S1, paste(path,"/UniqueAAsR4S1.csv", sep = ""),
 row.names = F)
write.csv(UniqueAAsR4S2, paste(path,"/UniqueAAsR4S2.csv", sep = ""),
 row.names = F)
write.csv(UniqueAAsR3S2, paste(path,"/UniqueAAsR3S2.csv", sep = ""),
row.names = F)
```

2. Comparative analysis of three different sequencing pools

```
library(prodlim)
library(VennDiagram)
library(ggplot2)
library(ggrepel)
library(plotly)
ncAA <- "AcrK" #change according to ncAA used
lib <- 12 #12 for CX10TAG, 14 for CX12C
path <- "/RhoA"

#Reads .csv files of UniqueAAs from Amino Acid Analysis and combines
 them into one dataframe with percentages from each round, Change paths
 and names of dataframes to match whichever samples you are working
 with
R4S2 <- read.csv("/RhoA/UniqueAAsR4S2.csv", header = TRUE)
R4S1 <- read.csv("/RhoA/UniqueAAsR4S1.csv")
```

```
R3S2 <- read.csv("/RhoA/UniqueAAsR3S2.csv")

R4S2[R4S2 == "TAG"] <- ncAA
R4S1[R4S1 == "TAG"] <- ncAA
R3S2[R3S2 == "TAG"] <- ncAA
R4S2 <- data.frame(apply(R4S2[,1:lib], 1, paste, collapse = ""),
 R4S2[,lib+1])
R4S1 <- data.frame(apply(R4S1[,1:lib], 1, paste, collapse = ""),
 R4S1[,lib+1])
R3S2 <- data.frame(apply(R3S2[,1:lib], 1, paste, collapse = ""),
 R3S2[,lib+1])
colnames(R4S2) <- c("Sequence", "nR4S2")
colnames(R4S1) <- c("Sequence", "nR4S1")
colnames(R3S2) <- c("Sequence", "nR3S2")
a <- row.match(R4S2[1], R4S1[1])
b <- row.match(R4S2[1], R3S2[1])
d <- row.match(R4S1[1], R4S2[1])
e <- row.match(R4S1[1], R3S2[1])
g <- row.match(R3S2[1], R4S2[1])
h <- row.match(R3S2[1], R4S1[1])
CombinedData <- R4S2
colnames(CombinedData)[2] <- "nR4S2"
CombinedData$nR4S1 <- R4S1[a,2]
CombinedData$nR3S2 <- R3S2[b,2]
R4S1$nR4S2 <- R4S2[d,2]
R4S1$nR3S2 <- R3S2[e,2]
R3S2$nR4S2 <- R4S2[g,2]
R3S2$nR4S1 <- R4S1[h,2]
CombinedData <- merge(CombinedData, R4S1, all.x = TRUE, all.y = TRUE)
CombinedData <- merge(CombinedData, R3S2, all.x = TRUE, all.y = TRUE)
CombinedData[is.na(CombinedData)] <- 0
CombinedData$percentR4S2 <-
 CombinedData$nR4S2/sum(CombinedData$nR4S2)*100
CombinedData$percentR4S1 <-
 CombinedData$nR4S1/sum(CombinedData$nR4S1)*100
CombinedData$percentR3S2 <-
 CombinedData$nR3S2/sum(CombinedData$nR3S2)*100
CombinedData <- CombinedData[order(-CombinedData$nR3S2),]

#Addition of Log2FoldChange to CombinedPlot, assumes 0.5 sequence
 number for sequences that are not found in a round
Log2Data <- CombinedData[,1:4]
Log2Data[Log2Data == 0] <- 0.5
Log2Data$percentR4S2 <- Log2Data$nR4S2/sum(CombinedData$nR4S2)*100
```

```
Log2Data$percentR4S1 <- Log2Data$nR4S1/sum(CombinedData$nR4S1)*100
Log2Data$percentR3S2 <- Log2Data$nR3S2/sum(CombinedData$nR3S2)*100
Log2Data$Log2R3S2vR4S2 <-
 log2(Log2Data$percentR3S2/Log2Data$percentR4S2)
Log2Data$Log2R3S2vR4S1 <-
 log2(Log2Data$percentR3S2/Log2Data$percentR4S1)
Log2Data$Log2R4S1vR4S2 <-
 log2(Log2Data$percentR4S1/Log2Data$percentR4S2)

#Finding Common Sequences
Common <- CombinedData
Common[Common == 0] <- NA
Common <- na.omit(Common)

totalpercentCommon <- sum(Common[,5:7])/3

#R4S2andR4S1Shared
R4S2andR4S1 <- CombinedData
test <- R4S2andR4S1$nR4S2 == 0 | R4S2andR4S1$nR4S1 == 0
R4S2andR4S1 <- R4S2andR4S1[which(test==FALSE),]

totalpercentR4S2andR4S1 <- sum(R4S2andR4S1[,5:7])/3

#R4S2andR4S1Only
R4S2andR4S1only <- R4S2andR4S1
test <- R4S2andR4S1$nR3S2 == 0
R4S2andR4S1only <- R4S2andR4S1[which(test==TRUE),]

totalpercentR4S2andR4S1only <- sum(R4S2andR4S1only[,5:7])/3

#R4S2andR3S2Shared
R4S2andR3S2 <- CombinedData
test <- R4S2andR3S2$nR4S2 == 0 | R4S2andR3S2$nR3S2 == 0
R4S2andR3S2 <- R4S2andR3S2[which(test==FALSE),]

totalpercentR4S2andR3S2 <- sum(R4S2andR3S2[,5:7])/3

#R4S2andR3S2Only
R4S2andR3S2only <- R4S2andR3S2
test <- R4S2andR3S2$nR4S1 == 0
R4S2andR3S2only <- R4S2andR3S2[which(test==TRUE),]

totalpercentR4S2andR3S2only <- sum(R4S2andR3S2only[,5:7])/3
```

```
#R4S1andR3S2Shared
R4S1andR3S2 <- CombinedData
test <- R4S1andR3S2$nR4S1 == 0 | R4S1andR3S2$nR3S2 == 0
R4S1andR3S2 <- R4S1andR3S2[which(test==FALSE),]

totalpercentR4S1andR3S2 <- sum(R4S1andR3S2[,5:7])/3

#R4S1and217only
R4S1andR3S2only <- R4S1andR3S2
test <- R4S1andR3S2$nR4S2 == 0
R4S1andR3S2only <- R4S1andR3S2only[which(test==TRUE),]

totalpercentR4S1andR3S2only <- sum(R4S1andR3S2only[,5:7])/3

#R4S2Unique
R4S2Unique <- CombinedData
test <- R4S2Unique$nR3S2 == 0 & R4S2Unique$nR4S1 == 0
R4S2Unique <- R4S2Unique[which(test==TRUE),]

totalpercentR4S2Unique <- sum(R4S2Unique[,5:7])/3

#R4S1Unique
R4S1Unique <- CombinedData
test <- R4S1Unique$nR3S2 == 0 & R4S1Unique$nR4S2 == 0
R4S1Unique <- R4S1Unique[which(test==TRUE),]

totalpercentR4S1Unique <- sum(R4S1Unique[,5:7])/3

#R3S2Unique
R3S2Unique <- CombinedData
test <- R3S2Unique$nR4S1 == 0 & R3S2Unique$nR4S2 == 0
R3S2Unique <- R3S2Unique[which(test==TRUE),]

totalpercentR3S2Unique <- sum(R3S2Unique[,5:7])/3

#Calculates total reads for all 3 samples
totalreads <- sum(CombinedData[,2:4])
R4S2total <- sum(CombinedData$nR4S2)
R4S1total <- sum(CombinedData$nR4S1)
R3S2total <- sum(CombinedData$nR3S2)


#Calculates Peptides > 0.1% for all samples
R4S2morethan0.1 <- length(which(CombinedData$percentR4S2>0.1))
```

```
R4S1morethan0.1 <- length(which(CombinedData$percentR4S1>0.1))
R3S2morethan0.1 <- length(which(CombinedData$percentR3S2>0.1))

#Calculates ratios for round3 enrichment plots
R4S2_10k <- length(which(CombinedData$nR4S2>10000))
R4S2_1k <- length(which(CombinedData$nR4S2>999 & CombinedData$nR4S2 <
 10000))
R4S2_100 <- length(which(CombinedData$nR4S2>99 & CombinedData$nR4S2 <
 1000))
R4S2_10 <- length(which(CombinedData$nR4S2>9 & CombinedData$nR4S2 <
 100))
R4S2_1 <- length(which(CombinedData$nR4S2>0.9 & CombinedData$nR4S2 <
 10))
percent10k85 <-
 sum(CombinedData[which(CombinedData$nR4S2>10000),"percentR4S2"])
percent1k85 <- sum(CombinedData[which(CombinedData$nR4S2>999 &
 CombinedData$nR4S2 < 10000),"percentR4S2"])
percent100_85 <- sum(CombinedData[which(CombinedData$nR4S2>99 &
 CombinedData$nR4S2 < 1000),"percentR4S2"])
percent10_85 <- sum(CombinedData[which(CombinedData$nR4S2>9 &
 CombinedData$nR4S2 < 100),"percentR4S2"])
percent1_85 <- sum(CombinedData[which(CombinedData$nR4S2>0.9 &
 CombinedData$nR4S2 < 10),"percentR4S2"])


R4S1_10k <- length(which(CombinedData$nR4S1>10000))
R4S1_1k <- length(which(CombinedData$nR4S1>999 & CombinedData$nR4S1 <
 10000))
R4S1_100 <- length(which(CombinedData$nR4S1>99 & CombinedData$nR4S1 <
 1000))
R4S1_10 <- length(which(CombinedData$nR4S1>9 & CombinedData$nR4S1 <
 100))
R4S1_1 <- length(which(CombinedData$nR4S1>0.9 & CombinedData$nR4S1 <
 10))
percent10k156 <-
 sum(CombinedData[which(CombinedData$nR4S1>10000),"percentR4S1"])
percent1k156 <- sum(CombinedData[which(CombinedData$nR4S1>999 &
 CombinedData$nR4S1 < 10000),"percentR4S1"])
percent100_156 <- sum(CombinedData[which(CombinedData$nR4S1>99 &
 CombinedData$nR4S1 < 1000),"percentR4S1"])
percent10_156 <- sum(CombinedData[which(CombinedData$nR4S1>9 &
 CombinedData$nR4S1 < 100),"percentR4S1"])
percent1_156 <- sum(CombinedData[which(CombinedData$nR4S1>0.9 &
 CombinedData$nR4S1 < 10),"percentR4S1"])
```

```
R3S2_10k <- length(which(CombinedData$nR3S2>10000))
R3S2_1k <- length(which(CombinedData$nR3S2>999 & CombinedData$nR3S2 <
 10000))
R3S2_100 <- length(which(CombinedData$nR3S2>99 & CombinedData$nR3S2 <
 1000))
R3S2_10 <- length(which(CombinedData$nR3S2>9 & CombinedData$nR3S2 <
 100))
R3S2_1 <- length(which(CombinedData$nR3S2>0.9 & CombinedData$nR3S2 <
 10))
percent10k217 <-
 sum(CombinedData[which(CombinedData$nR3S2>10000),"percentR3S2"])
percent1k217 <- sum(CombinedData[which(CombinedData$nR3S2>999 &
 CombinedData$nR3S2 < 10000),"percentR3S2"])
percent100_217 <- sum(CombinedData[which(CombinedData$nR3S2>99 &
 CombinedData$nR3S2 < 1000),"percentR3S2"])
percent10_217 <- sum(CombinedData[which(CombinedData$nR3S2>9 &
 CombinedData$nR3S2 < 100),"percentR3S2"])
percent1_217 <- sum(CombinedData[which(CombinedData$nR3S2>0.9 &
 CombinedData$nR3S2 < 10),"percentR3S2"])


#Creates Venn Diagram of Peptides, see
 https://www.datanovia.com/en/blog/venn-diagram-with-r-or-rstudio-a-
 million-ways/ and https://www.sthda.com/english/wiki/colors-in-r
peptidelist <- list(A = R4S2[,1], B = R4S1[,1], C = R3S2[,1])
display_venn <- function(x, ...){
  library(VennDiagram)
  grid.newpage()
  venn_object <- venn.diagram(x, filename = NULL, ...)
  grid.draw(venn_object)
}
display_venn(
  peptidelist,
  category.names = c("R4S2" , "R4S1" , "R3S2"),
  # Circles
  lwd = 2,
  lty = 'blank',
  fill = c("blue", "yellow", "red"),
  # Numbers
  cex = .9,
  fontface = 4,
  # Set names
```

```
  cat.cex = 1,
  cat.fontface = "bold",
  cat.default.pos = "outer"
)

#Makes Enrichment plot for R4S2
df <- data.frame(bin=c("1-9","10-99","100-999","1000-9999","10000+"))
df$sumpercent <-
 c(percent1_85,percent10_85,percent100_85,percent1k85,percent10k85)
df$unique <- c(R4S2_1,R4S2_10,R4S2_100,R4S2_1k,R4S2_10k)
df$y <- cumsum(df$sumpercent)
R4S2EnrichmentSummary <- df

ggplot(df, aes(ymin = 0, ymax = 100, xmin = 0, xmax = 10000, fill =
 bin)) +
  geom_rect(mapping = aes(xmin = 0, xmax = unique, ymin = y-
 sumpercent, ymax = y)) +
  scale_x_log10() +
  scale_fill_manual(values = c("#CCCCCC", "#ADD8E6", "#6495ED",
 "#4169E1","#000066")) # Light, medium, dark blue

#Makes Enrichment plot for R4S1
df <- data.frame(bin=c("1-9","10-99","100-999","1000-9999","10000+"))
df$sumpercent <-
 c(percent1_156,percent10_156,percent100_156,percent1k156,percent10k156
 )
df$unique <- c(R4S1_1,R4S1_10,R4S1_100,R4S1_1k,R4S1_10k)
df$y <- cumsum(df$sumpercent)
R4S1enrichmentsummary <- df

ggplot(df, aes(ymin = 0, ymax = 100, xmin = 0, xmax = 10000, fill =
 bin)) +
  geom_rect(mapping = aes(xmin = 0, xmax = unique, ymin = y-
 sumpercent, ymax = y)) +
  scale_x_log10() +
  scale_fill_manual(values = c("#CCCCCC", "#ADD8E6", "#6495ED",
 "#4169E1","#000066")) # Light, medium, dark blue

#Makes Enrichment plot for R3S2
df <- data.frame(bin=c("1-9","10-99","100-999","1000-9999","10000+"))
df$sumpercent <-
 c(percent1_217,percent10_217,percent100_217,percent1k217,percent10k217
 )
df$unique <- c(R3S2_1,R3S2_10,R3S2_100,R3S2_1k,R3S2_10k)
```

```
df$y <- cumsum(df$sumpercent)
R3S2EnrichmentSummary <- df

ggplot(df, aes(fill = bin)) +
  geom_rect(mapping = aes(xmin = 0, xmax = unique, ymin = y-
 sumpercent, ymax = y)) +
  scale_x_log10() +
  scale_fill_manual(values = c("#CCCCCC", "#ADD8E6", "#6495ED",
 "#4169E1","#000066")) # Light, medium, dark blue

# Assign colors based on conditions
Log2Data$color <- ifelse(Log2Data$nR4S2 == 0.5 & Log2Data$nR4S1 ==
 0.5, "red",
                    ifelse(Log2Data$nR4S1 == 0.5 & Log2Data$nR3S2 ==
 0.5, "blue",
                           ifelse(Log2Data$nR3S2 == 0.5 &
 Log2Data$nR4S2 == 0.5, "yellow",
                                  ifelse(Log2Data$nR4S2 != 0.5 &
 Log2Data$nR4S1 != 0.5 & Log2Data$nR3S2 == 0.5, "green",
                                         ifelse(Log2Data$nR4S2 != 0.5
 & Log2Data$nR4S1 == 0.5 & Log2Data$nR3S2 != 0.5, "purple",
                                                ifelse(Log2Data$nR4S2
 == 0.5 & Log2Data$nR4S1 != 0.5 & Log2Data$nR3S2 != 0.5, "orange",
 "black")
                                         )))))  # Default color for
 other points
topR4S2colors <- table(Log2Data[which(Log2Data$percentR4S2 >
 0.1),"color"])
topR4S1colors <- table(Log2Data[which(Log2Data$percentR4S1 >
 0.1),"color"])
topR3S2colors <- table(Log2Data[which(Log2Data$percentR3S2 >
 0.1),"color"])

# Generate scatter plot with log-transformed axes, change data and
 axes as needed
p <- ggplot(Log2Data, aes(x = percentR4S2, y = percentR3S2, color =
 color, text = Sequence)) +
  geom_point(size = 1.5, alpha = 1) +
  geom_vline(xintercept = 1e-1, linetype = "dashed", color = "black",
 linewidth = 0.6) +
  geom_hline(yintercept = 1e-1, linetype = "dashed", color = "black",
 linewidth = 0.6) +
  scale_color_identity() +  # Uses predefined colors without a legend
  scale_x_log10() +  # Log transform the X-axis
```

```
  scale_y_log10() +  # Log transform the Y-axis
  labs(x = "R4S2%", y = "R3S2%") +
  theme_minimal() +
  theme(
    axis.line = element_line(linewidth = 1.2, color = "black"),
    axis.text = element_text(size = 12),
    axis.title = element_text(size = 14, face = "bold"),
    panel.grid.major = element_line(color = "gray90"),
    panel.grid.minor = element_blank()
  )
p
ggplotly(p, tooltip = c("percentR4S2","percentR3S2","text"))
 #generates interactive html file for looking at the data, can save as
 html and view later
#write csv files for log2Data and CombinedData, Change path names
 according to sample
write.csv(CombinedData, paste(path, "/CombinedDataCX10.csv", sep =
 ""), row.names = F)
write.csv(Log2Data, paste(path, "/Log2DataCX10.csv", sep = ""),
row.names = F)
```

3. UMAP generation from three different peptide pools

```
#UMAP for peptides
library(Peptides)
library(uwot)
library(ggplot2)
library(plotly)
library(prodlim)
ncAA <- "*" #ncAA used during selection or what it should be displayed
 as in the UMAP
lib <- 12 #Library insert region size (12 for CX10TAG, 14 for CX12C)
path <- "/RhoA" #change path directory accordingly

#Reads .csv files of UniqueAAs from Amino Acid Analysis and
 concatenates the AAs, change dataframe names accordingly
R4S2 <- read.csv("/RhoA/UniqueAAsR4S2.csv", header = TRUE)
R4S1 <- read.csv("/RhoA/UniqueAAsR4S1.csv")
R3S2 <- read.csv("/RhoA/UniqueAAsR3S2.csv")

R4S2[R4S2 == "TAG"] <- ncAA
R4S1[R4S1 == "TAG"] <- ncAA
```

```
R3S2[R3S2 == "TAG"] <- ncAA
R4S2 <- data.frame(apply(R4S2[,1:lib], 1, paste, collapse = ""),
 R4S2[,lib+1])
R4S1 <- data.frame(apply(R4S1[,1:lib], 1, paste, collapse = ""),
 R4S1[,lib+1])
R3S2 <- data.frame(apply(R3S2[,1:lib], 1, paste, collapse = ""),
 R3S2[,lib+1])
colnames(R4S2) <- c("Sequence", "nR4S2")
colnames(R4S1) <- c("Sequence", "nR4S1")
colnames(R3S2) <- c("Sequence", "nR3S2")

#Creates Combined Dataframe with all sequencing pools
a <- row.match(R4S2[1], R4S1[1])
b <- row.match(R4S2[1], R3S2[1])
d <- row.match(R4S1[1], R4S2[1])
e <- row.match(R4S1[1], R3S2[1])
g <- row.match(R3S2[1], R4S2[1])
h <- row.match(R3S2[1], R4S1[1])
CombinedData <- R4S2
colnames(CombinedData)[2] <- "nR4S2"
CombinedData$nR4S1 <- R4S1[a,2]
CombinedData$nR3S2 <- R3S2[b,2]
R4S1$nR4S2 <- R4S2[d,2]
R4S1$nR3S2 <- R3S2[e,2]
R3S2$nR4S2 <- R4S2[g,2]
R3S2$nR4S1 <- R4S1[h,2]
CombinedData <- merge(CombinedData, R4S1, all.x = TRUE, all.y = TRUE)
CombinedData <- merge(CombinedData, R3S2, all.x = TRUE, all.y = TRUE)
CombinedData[is.na(CombinedData)] <- 0
CombinedData$percentR4S2 <-
 CombinedData$nR4S2/sum(CombinedData$nR4S2)*100
CombinedData$percentR4S1 <-
 CombinedData$nR4S1/sum(CombinedData$nR4S1)*100
CombinedData$percentR3S2 <-
 CombinedData$nR3S2/sum(CombinedData$nR3S2)*100
CombinedData <- CombinedData[order(-CombinedData$nR3S2),]

#Addition of Log2FoldChange to CombinedPlot, assumes 0.5 sequence
 number for sequences that are not found in a round
Log2Data <- CombinedData[,1:4]
Log2Data[Log2Data == 0] <- 0.5
Log2Data$percentR4S2 <- Log2Data$nR4S2/sum(CombinedData$nR4S2)*100
Log2Data$percentR4S1 <- Log2Data$nR4S1/sum(CombinedData$nR4S1)*100
Log2Data$percentR3S2 <- Log2Data$nR3S2/sum(CombinedData$nR3S2)*100
```

```
Log2Data$Log2R3S2vR4S2 <-
 log2(Log2Data$percentR3S2/Log2Data$percentR4S2)
Log2Data$Log2R3S2vR4S1 <-
 log2(Log2Data$percentR3S2/Log2Data$percentR4S1)
Log2Data$Log2R4S1vR4S2 <-
 log2(Log2Data$percentR4S1/Log2Data$percentR4S2)

Log2Data$color <- ifelse(Log2Data$nR4S2 == 0.5 & Log2Data$nR4S1 ==
 0.5, "red",
                         ifelse(Log2Data$nR4S1 == 0.5 & Log2Data$nR3S2
 == 0.5, "blue",
                                ifelse(Log2Data$nR3S2 == 0.5 &
 Log2Data$nR4S2 == 0.5, "yellow",
                                       ifelse(Log2Data$nR4S2 != 0.5 &
 Log2Data$nR4S1 != 0.5 & Log2Data$nR3S2 == 0.5, "green",
                                              ifelse(Log2Data$nR4S2 !=
 0.5 & Log2Data$nR4S1 == 0.5 & Log2Data$nR3S2 != 0.5, "purple",

 ifelse(Log2Data$nR4S2 == 0.5 & Log2Data$nR4S1 != 0.5 & Log2Data$nR3S2
 != 0.5, "orange", "black")
                                              )))))  # Default color
 for other points

#Extracts top100 sequences from each and combines into one dataframe
top_R4S2 <- Log2Data[order(Log2Data$percentR4S2, decreasing = TRUE),
 ][1:100, ]
top_R3S2 <- Log2Data[order(Log2Data$percentR3S2, decreasing = TRUE),
 ][1:100, ]
top_R4S1 <- Log2Data[order(Log2Data$percentR4S1, decreasing = TRUE),
 ][1:100, ]
top_combined <- unique(rbind(top_R4S2, top_R4S1, top_R3S2))


#Creates UMAP projection
peps <- top_combined[,1]

features <- data.frame(
  hydrophobicity = sapply(peps, hydrophobicity, scale =
 "KyteDoolittle"),
  charge = sapply(peps, charge, pH = 7),
  mass = sapply(peps, mw),
  aliphatic = sapply(peps, aIndex)
)
features$sequence <- peps
```

```
# UMAP projection
umap_res <- umap(features[, 1:5])
umap_df <- as.data.frame(umap_res)
colnames(umap_df) <- c("UMAP_1", "UMAP_2")
umap_df$sequence <- peps
umap_df$nR4S2 <- top_combined$nR4S2
umap_df$nR4S1 <- top_combined$nR4S1
umap_df$nR3S2 <- top_combined$nR3S2
umap_df$color <- top_combined$color
umap_df$mean <- (rowMeans(umap_df[, c("nR4S2", "nR4S1", "nR3S2")],
 na.rm = TRUE))

# Plot
p <- ggplot(umap_df, aes(x = UMAP_1, y = UMAP_2, text = sequence, size
 = mean, color = color)) +
  scale_color_identity() +
  geom_point(alpha = 0.8) +  #adjust opacity as needed
  scale_size(range = c(1, 12), name = "Mean Abundance", breaks =
 c(100,500,1000,5000,10000)) + #legend and sizing of points
  theme_minimal() +
  labs(title = "UMAP of Peptide Physicochemical Features")
p
ggplotly(p,tooltip = c("sequence","n"))

#Write csv files, change path accordingly
write.csv(umap_df, paste(path, "/UMAPCX10.csv", sep = ""), row.names =
 F)
write.csv(features, paste(path, "/FeaturesCX10.csv", sep = ""),
 row.names = F)
write.csv(top_combined, paste(path, "/Log2Top100_CX10.csv", sep = ""),
 row.names = F)
```
